# Clinical effectiveness of rehabilitation in ambulatory care for patients with persisting symptoms after COVID-19: a systematic review

**DOI:** 10.1186/s12879-023-08374-x

**Published:** 2023-06-21

**Authors:** Hannelore Dillen, Geertruida Bekkering, Sofie Gijsbers, Yannick Vande Weygaerde, Maarten Van Herck, Sarah Haesevoets, David A G Bos, Ann Li, Wim Janssens, Rik Gosselink, Thierry Troosters, Jan Y Verbakel

**Affiliations:** 1 EPI-Centre, Department of Public Health and Primary Care, KU Leuven, 7 Kapucijnenvoer, 3000 Leuven, Belgium; 2 Academic Centre for General Practice, Department of Public Health and Primary Care, KU Leuven, 7 Kapucijnenvoer, 3000 Leuven, Belgium; 3 Centre for Evidence-Based Medicine, 7 Kapucijnenvoer, 3000 Leuven, Belgium; 4 Cochrane Belgium, 7 Kapucijnenvoer, 3000 Leuven, Belgium; 5 post-COVID community, Belgium; 6 Department of Respiratory Medicine, Ghent University Hospital, 10 Corneel Heymanslaan, 9000 Ghent, Belgium; 7 REVAL–Rehabilitation Research Center, Biomedical Research Institute (BIOMED), Faculty of Rehabilitation Sciences, Hasselt University, Agoralaan Building A, 3590 Diepenbeek, Belgium; 8 Department of Research and Development, Ciro, 1 Hornerheide, Horn, 6085 NM The Netherlands; 9 Department of Respiratory Medicine, NUTRIM School of Nutrition and Translational Research in Metabolism, Maastricht University Medical Centre+, 25 P. Debyelaan, Maastricht, 6229 HX The Netherlands; 10 Department of Respiratory Diseases, KU Leuven University Hospitals Leuven, 49 Herestraat, 3000 Leuven, Belgium; 11 Department of Rehabilitation Sciences, Research Group for Rehabilitation in Internal Disorders, KU Leuven, 101 Tervuursevest, 3001 Leuven, Belgium; 12 NIHR Community Healthcare Medtech and IVD Cooperative, Nuffield Department of Primary Care Health Sciences, University of Oxford, Woodstock Road, Oxford, OX2 6GG UK

**Keywords:** COVID-19, Post-acute COVID-19 syndrome, Rehabilitation, Ambulatory care

## Abstract

**Background:**

Lingering symptoms after acute COVID-19 present a major challenge to ambulatory care services. Since there are reservations regarding their optimal management, we aimed to collate all available evidence on the effects of rehabilitation treatments applicable in ambulatory care for these patients.

**Methods:**

On 9 May 2022, we systematically searched articles in COVID-19 collections, Embase, MEDLINE, Cochrane Library, Web of Science, CINAHL, PsycArticles, PEDro, and EuropePMC. References were eligible if they reported on the clinical effectiveness of a rehabilitation therapy applicable in ambulatory care for adult patients with persisting symptoms continuing 4 weeks after the onset of COVID-19. The quality of the studies was evaluated using the CASP cohort study checklist and the Cochrane Risk of Bias Assessment Tool. Summary of Findings tables were constructed and the certainty of evidence was assessed using the GRADE framework.

**Results:**

We included 38 studies comprising 2,790 participants. Physical training and breathing exercises may reduce fatigue, dyspnoea, and chest pain and may improve physical capacity and quality of life, but the evidence is very weak (based on 6 RCTs and 12 cohort studies). The evidence underpinning the effect of nutritional supplements on fatigue, dyspnoea, muscle pain, sensory function, psychological well-being, quality of life, and functional capacity is very poor (based on 4 RCTs). Also, the evidence-base is very weak about the effect of olfactory training on sensory function and quality of life (based on 4 RCTs and 3 cohort studies). Multidisciplinary treatment may have beneficial effects on fatigue, dyspnoea, physical capacity, pulmonary function, quality of life, return to daily life activities, and functional capacity, but the evidence is very weak (based on 5 cohort studies). The certainty of evidence is very low due to study limitations, inconsistency, indirectness, and imprecision.

**Conclusions:**

Physical training, breathing exercises, olfactory training and multidisciplinary treatment can be effective rehabilitation therapies for patients with persisting symptoms after COVID-19, still with high uncertainty regarding these effects. These findings can guide ambulatory care practitioners to treat these patients and should be incorporated in clinical practice guidelines. High-quality studies are needed to confirm our hypotheses and should report on adverse events.

**Supplementary Information:**

The online version contains supplementary material available at 10.1186/s12879-023-08374-x.

## Background

Severe acute respiratory syndrome coronavirus 2 (SARS-CoV-2), the pathogen responsible for coronavirus disease 2019 (COVID-19), has caused considerable morbidity and mortality at an unprecedented global scale [1]. Evidence on the subacute and longer-term effects of COVID-19 is evolving worldwide [1]. Persisting symptoms following COVID-19 can be defined as ‘ongoing symptomatic COVID-19’ (symptoms lasting 4 to 12 weeks) and ‘post-COVID-19 syndrome’ (symptoms beyond 12 weeks) according to the National Institute for Health and Care Excellence (NICE)’s terms [2], ‘post-COVID-19 condition’ as named by the World Health Organisation (WHO) [3], and ‘post-COVID conditions’ as referred to by the United States Center for Disease Control and Prevention’s (CDC) group [4]. It is estimated that at an average follow-up time of 4 months, 45% of COVID-19 survivors exhibit at least one unresolved symptom [5]. The incidence is even higher among previously hospitalised patients, reaching 53% [5]. The most commonly reported symptoms are fatigue, dyspnoea, (muscle) pain, affected sleep, impaired usual activity, and loss of smell and taste [5]. Therefore, this is a complex, multifaceted condition affecting multiple organ systems [5]. Also its pathogenesis is likely multifactorial and it includes prolonged inflammation, immune-mediated vascular dysfunction, thromboembolism, and nervous system dysfunction [6]. Risk factors may include female sex, increasing age, having two or more comorbidities, a more severe acute COVID-19 illness, and a higher number of symptoms during the acute illness [7–9]. Increased levels of D-dimer or C-reactive protein or reduced lymphocyte count during the acute illness may also be prognostic factors [7]. Further research is needed to better define these risk factors, to understand the underlying mechanisms, and to address the neuropsychological components and its impact on this new clinical disease entity [6, 7]. Currently, there are reservations regarding the optimal management of patients with persisting symptoms after COVID-19, as there is insufficient evidence on the mechanisms that underpin this condition [10]. The WHO suggests that rehabilitation for these patients requires a person-centred, comprehensive, and multidisciplinary approach [11]. Interventions for rehabilitation may include education, skills training on self-management strategies, advice on paced return to activities, breathing techniques (including respiratory muscle training), physical exercise therapy, psychological interventions, cognitive training, rehabilitation for communication and swallowing difficulties, and occupational therapy [11]. These interventions should be provided in close collaboration with primary healthcare and several medical specialties [11].

We aimed to collate all available evidence on the effects of rehabilitation treatments, applicable in ambulatory care, for patients with persisting symptoms after COVID-19. This review is part of the development of a guideline on the follow-up and rehabilitation of patients with persisting symptoms after COVID-19 in primary care, which was commissioned by the Belgian government.

## Methods

We performed a systematic review according to the methods as described by Cochrane [12] and reported it according to the Preferred Reporting Items for Systematic Reviews and Meta-Analyses (PRISMA) 2020 Checklist (Additional File 1) [13].

### Search strategy

First, we screened existing COVID-19 libraries, namely Research Aid Networks Long Covid Library [14] and Resources LongCovid (Care) [15] and web-based COVID-19 collections, i.e. Cochrane COVID-19 Study Register [16], Epistemonikos [17], and WHO COVID-19 database [18]. In addition, we searched the following literature databases: Embase, MEDLINE, Cochrane Library, Web of Science Core Collection, CINAHL, PsycArticles, PEDro, and EuropePMC (preprints). The search string was based on two concepts: ‘persisting symptoms after COVID-19’ and ‘rehabilitation’. Full search strategies for each database are included in Additional File 1. We also screened the references included in NICE’s COVID-19 rapid guideline: ‘managing the long-term effects of COVID-19’ [2]. Experts from the Belgian guideline working group were also requested to verify the list of retrieved publications and add publications when missing.

### Inclusion and exclusion criteria

References were eligible if they reported on the clinical effectiveness of a rehabilitation therapy applicable in ambulatory care for patients with persisting symptoms after COVID-19 (i.e., new or ongoing symptoms continuing after 4 weeks from the onset of acute COVID-19). Studies investigating a rehabilitation therapy contiguous to hospitalisation were excluded because this was not the population of interest (i.e., these patients were often more seriously ill and often had specific problems). We included randomised controlled trials (RCTs), non-randomised trials, prospective and retrospective cohort studies, cross-sectional studies, case–control studies, and case series with at least 10 patients. Systematic reviews, case reports, letters, editorials, qualitative studies, conference abstracts, posters, and protocols were excluded. Preprints were not included in the final analysis, but it was checked whether the corresponding articles had already been published. We only considered studies on adults (≥ 18 years) who had experienced symptomatic and confirmed (by polymerase chain reaction, antibodies, or chest CT) COVID-19. We excluded studies on nursing home residents and people with specific comorbidities (e.g., heart failure, diabetes), except if these populations constituted less than 20% of the sample size. There were no restrictions in terms of language, country, race, or gender. We excluded studies investigating the effectiveness of individual molecules and synthetic drugs, except for over the counter nutritional supplements (such as vitamins). If molecules were evaluated on top of a rehabilitation therapy, the study was included.

### Selection and data extraction

Search results were imported into a reference management program (Endnote 20.2 (Bld 15,709), Clarivate Analytics) and duplicate citations were removed [19]. Based on inclusion and exclusion criteria, pairs of two reviewers (11 reviewers in total) independently screened all records by title and abstract, using Rayyan (Rayyan Systems Inc.) [20] and Covidence [21] software (Covidence systematic review software, Veritas Health Innovation, Melbourne, Australia. Available at www.covidence.org.). Next, pairs of two reviewers independently reviewed the full text of all potentially relevant records, using the same selection criteria. Data were extracted by one reviewer (HD) and checked by another reviewer (GB, DP) using standardised data extraction forms in Covidence and Microsoft Excel (Version 2202). Any queries or disagreements in either of the steps above were resolved through discussion or, if necessary, another reviewer. The following data items were extracted: title, authors, publication year, journal, publication status, country, timeframe of patient recruitment, study design, population characteristics (i.e., inclusion and exclusion criteria, setting, sample size, age, sex, follow-up time since acute COVID-19, acute COVID-19 disease severity, hospitalisation or intensive care unit use during acute COVID-19 illness), rehabilitation therapy, comparator(s), primary and secondary endpoints, and the main results. Data were sought for the following outcome measures: fatigue, dyspnoea, muscle pain, chest pain, physical capacity (i.e., physical fitness and muscle performance), pulmonary function, cognitive function, sensory function (i.e., smell and taste), psychological well-being, quality of life, return to normal daily life activities, functional capacity (i.e., ability to perform ‘activities of daily living’), and adverse events. All results that were compatible with each outcome were extracted (i.e., all measures, time points, and analyses), except for pulmonary function for which we only extracted data for the maximal voluntary ventilation (MVV), vital capacity (VC), and maximal inspiratory pressure (MIP) as these were considered as measurable in an ambulatory care setting. If needed, corresponding authors were contacted for additional study information.

### Risk of bias assessment

The methodological quality of the selected studies was evaluated independently by pairs of two reviewers (HD, GB, SG) using the CASP cohort study checklist for cohort studies [22] and the Cochrane Risk of Bias Assessment Tool 2.0 for RCTs [23].

### Data analysis and certainty of evidence assessment

Due to a high heterogeneity between studies, results were summarised narratively. We constructed Summary of Findings (SoF) tables to summarize all available evidence by intervention type (i.e., physical training program, breathing exercises, nutritional supplements, olfactory training, and multidisciplinary treatment) and subsequently by study design. For each outcome measure, we assessed certainty of evidence using the methods of the Grading of Recommendation Assessment, Development, and Evaluation (GRADE) framework [24, 25]. GRADEpro was used to create the SoF tables (GRADEpro Guideline Development Tool. McMaster University and Evidence Prime, 2022. Available from gradepro.org.).

We performed vote counting to determine by outcome domain the number of studies demonstrating beneficial results, no significant improvement, and mixed results (i.e., some measures showed beneficial results, while other measures within the same study did not show a significant improvement).

### Protocol registration

We published and prospectively registered the protocol for this systematic review on PROSPERO (CRD42022330205).

## Results

### Identification of studies

**Fig. 1:**
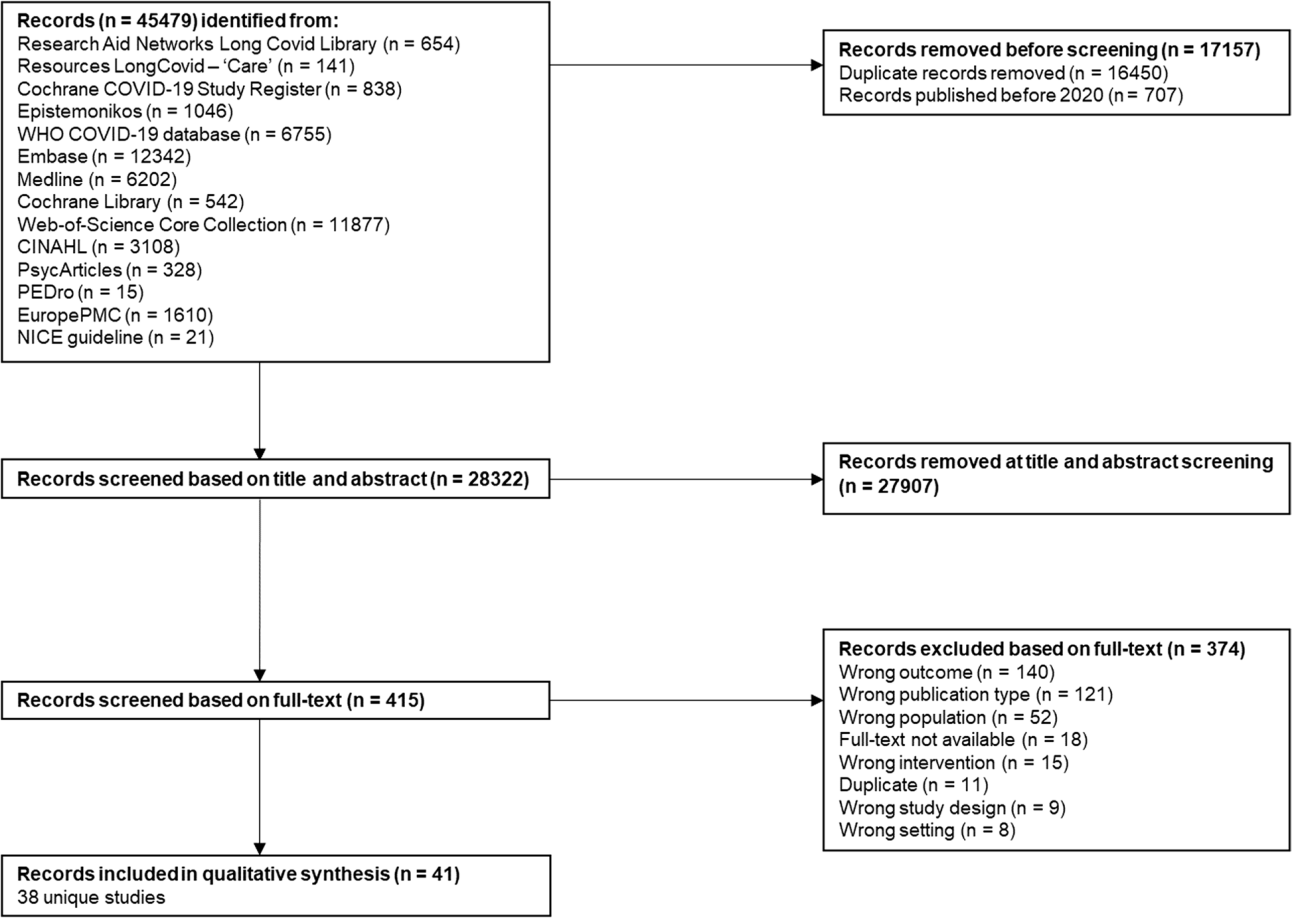
PRISMA flow-chart. Study identification and process for selection of studies included in the review

From the database search on 9 May 2022, 45,479 records were identified, of which 28,322 were screened for title and abstract. Of those, 415 references were screened based on full-text, whereof 38 unique studies were retained for this review (Fig. 1).

Physical training programs were evaluated by 14 studies, breathing exercises by 10 studies, nutritional supplements by four studies, olfactory training by seven studies, and multidisciplinary treatment programs were assessed by five studies. Seven studies evaluated other interventions which do not fall within these categories (i.e., narrative exposure therapy, aromatherapy, hydrogen inhalation, massage techniques, hyperbaric oxygen therapy, enhanced external counterpulsation). Characteristics of included studies are presented in Table 1.

**Table 1:** Characteristics of included studies

Reference	Study design^a^	Sample size^b^	Patient population	Intervention studied
**I. Physical training program only**				
Barbara, 2022 [26]	C	50	Patients with a reduced exercise capacity due to COVID-19	Aerobic exercises and muscle resistance exercises
Betschart, 2021 [27]	C	12	Previously hospitalised COVID-19 patients (average 41 days after COVID-19 diagnosis)	Aerobic training, resistance training, education, and physical activity coaching
Bouteleux, 2021 [28]	C	39	Patients referred to ambulatory respiratory rehabilitation following COVID-19	Aerobic training, strength training, and controlled ventilation techniques
Daynes, 2021 [29]	C	30	Patients with lasting symptoms of COVID-19	Aerobic exercises, strength training, education, and pacing advice
Hameed, 2021 [30]	C	106	Previously hospitalised COVID-19 patients with persistent symptoms, difficulty weaning from supplemental oxygenation or discharged from an acute rehabilitation unit with need to continue psychiatry-led care	Remote and home physiotherapy
Kireyev, 2022 [31]	C	22	Previously hospitalised COVID-19 patients with asthenic syndrome as a manifestation of Post-COVID-19 neurological syndrome	Physical exercises and brain activity stimulators
Martin, 2021 [32]	C	15	Previously hospitalised COVID-19 (severe or critical) patients	Endurance exercises, strength exercises, and encouragement (telerehabilitation)
Nambi, 2021 [33]	RCT^c^	76	Men with post-COVID-19 sarcopenia	Resistance training with high- or low-intensity aerobic training
**II. Breathing exercises only**				
Cahalan, 2022 [34]	C	27	People experiencing respiratory symptoms and/or increased fatigue at least 28 days after their COVID-19 diagnosis	Breathing retraining and singing classes (SingStrong LC)
Liu, 2020 [35]	RCT	76	Elderly patients with a history of COVID-19 (previously hospitalised)	Respiratory muscle training, cough exercise, diaphragmic training, stretching exercises, and home exercises
Philip, 2022 [36]	RCT	150	Patients recovering from COVID-19 with ongoing breathlessness (at least 4 weeks)	Online breathing and wellbeing programme
Srinivasan, 2021 [37]	RCT	48	Patients who visited the post-COVID-19 follow-up clinic	Pursed lip breathing and Bhastrika Pranayama
**III. Physical training program combined with breathing exercises**				
Ahmed, 2021 [38]	C	20	Previously hospitalised COVID-19 patients (average 25 days after COVID-19 diagnosis)	Aerobic training and breathing exercises
Dalbosco-Salas, 2021 [39]	C	150	Previously hospitalised COVID-19 patients with persisting dyspnoea	Aerobic and/or strength exercises, breathing exercises, stretching, and weekly phone calls with a physiotherapist (telerehabilitation)
Kokhan, 2021 [40]	C	74	Previously hospitalised COVID-19 patients (moderate to severe)	Low to medium intensity exercises and breathing exercises
Li, 2021 [41]	RCT	120	Previously hospitalised COVID-19 patients with remaining dyspnoea	Breathing control and thoracic expansion, aerobic exercises, limb muscle exercises (home-based)
Scaturro, 2022 [42]	RCTc	27	Patients having at least one fibromyalgia-like symptom at least 60 days after healing from COVID-19	Physical exercises and respiratory physio-kinesiotherapy (hospital-based)
Stavrou, 2021 [43]	C	26	Previously hospitalised COVID-19 patients (average 2 months after discharge)	Aerobic walking, yoga exercises, and multi-joint strength exercises (home-based)
**IV. Nutritional supplements**				
D’Ascanio, 2021 [44]	RCT	12	Adults with a history of COVID-19 and with persisting anosmia or hyposmia	Olfactory training and daily treatment with palmitoylethanolamide / luteolin
Di Stadio, 2022 [45]	RCT	185	Patients with a history of COVID-19 and anosmia or hyposmia persisting for at least 180 days after subsequent negative COVID-19 nasopharyngeal swab	Olfactory training and daily treatment with palmitoylethanolamide / luteolin
Rathi, 2021 [46]	RCT	200	Patients with a history of COVID-19 and experiencing fatigue and muscle weakness	Systemic enzymes (ImmunoSEB) and probiotics (ProbioSEB)
Scaturro, 2022 [42]	RCT	60	Patients having at least one fibromyalgia-like symptom at least 60 days after healing from COVID-19	Physical exercises, respiratory physio-kinesiotherapy, and L-acetyl-carnitine (hospital-based)
**V. Olfactory training**				
Abdelalim, 2021 [47]	RCT^c^	50	Recovered COVID-19 patients suffering from sudden recent anosmia or hyposmia	Olfactory training
D’Ascanio, 2021 [44]	RCT^c^	12	Adults with a history of COVID-19 and with persisting anosmia or hyposmia	Olfactory training
Denis, 2021 [48]	C	548	Patients with SARS-CoV-2-related olfactory dysfunction of at least one month	Olfactory training and visual stimulation
Di Stadio, 2022 [45]	RCT^c^	55	Patients with a history of COVID-19 and anosmia or hyposmia persisting for at least 180 days after subsequent negative COVID-19 nasopharyngeal swab	Olfactory training
Le Bon, 2021 [49]	C	18	Non-hospitalised adults with loss of smell due to COVID-19	Olfactory training
Pires, 2022 [50]	RCT^c^	80	Patients with complaints of olfactory alteration that persisted for at least 4 weeks after the onset of COVID-19 symptoms	Olfactory training (advanced versus classical)
Vandersteen, 2022 [51]	C	43	Patients contaminated by COVID-19 with persistent olfactory disorders lasting more than 6 weeks	Olfactory training
**VI. Multidisciplinary treatment**				
Albu / García-Molina, 2021 [52, 53]	C	43	Adults with neurological, cognitive and musculoskeletal sequelae and persistent symptoms of COVID-19 infection	Ambulatory rehabilitation program: physiotherapy, cognitive (neuropsychological), and respiratory rehabilitation
Everaerts, 2021 [54]	C	22	Adults previously hospitalised with COVID-19, a reduced limb muscle force or 6-min walking test and a deteriorated functional status	Multidisciplinary respiratory rehabilitation (ambulatory): treadmill, cycle ergometer, arm ergometer, stair / step climbing, and resistance training
Gloeckl, 2021 [55]	C	50	Patients in the post-acute phase of COVID-19	Multidisciplinary inpatient rehabilitation program: endurance and strength training, patient education, respiratory physiotherapy, activities of daily living training, relaxation techniques, occupational therapy, psychological support, and nutritional counselling
Hayden, 2021 [56]	C	53	Adults with persistent symptoms after COVID-19	Inpatient pulmonary rehabilitation program: physical training, respiratory and general physiotherapy, patient information, routine medical diagnostics, close medical supervision, psychological support, nutritional counselling, and occupational therapy
Nopp, 2022 [57]	C	64	Adults with persistent or progressive symptoms after COVID-19	Multi-professional individualised rehabilitation (ambulatory): endurance, strength, and inspiratory muscle training
**VII. Other interventions**				
Babliuk, 2022 [58]	C	60	Patients with symptoms of postcovid syndrome (history of COVID-19 approximately 12 weeks ago), who were referred to a physical rehabilitation department	Massage of the neck area, galvanization, low-frequency magnetic therapy, and electrosleep procedure
Fan, 2021 [59]	RCT	111	Patients previously hospitalised with COVID-19 and a score of PTSD Checklist-Civilian version of at least 50	Narrative exposure therapy, personalised psychological treatment, and 6-month online follow-up
Hawkins, 2022 [60]	RCT	44	Patients recovered from COVID-19 for 5 or more months and experiencing fatigue at a level that was not present prior to COVID-19	Aromatherapy blend of thyme, orange peel, clove bud, and frankincense
Heald, 2022 [61]	C	20	Patients with fatigue continuing for more than 12 weeks after an acute COVID-19 infection (Long COVID)	Treatments sessions with practitioners (effleurage of the neck / back / chest; soft tissue stretching, gentle cranial osteopathic techniques) and home-based self-massage routine
Robbins, 2021 [62]	C	10	Patients suffering from severe, longstanding post-COVID-19 syndrome (new fatigue continuing for more than 12 weeks)	Hyperbaric oxygen therapy
Sathyamoorthy, 2022 [63]	C	16	Patients with a history of COVID-19 and referred for the management of Long COVID-related symptoms	Enhanced external counterpulsation
Shogenova, 2021 [64]	RCT	60	Patients with a history of COVID-19 and 2 major and not less than 6 minor diagnostic signs of chronic fatigue syndrome	Physiotherapy, adjunctive drug therapy (magnesium, B vitamins, L-carnitine), and hydrogen inhalation

^a^*C* cohort study, *RCT* randomized controlled trial

^b^The number of patients that started the rehabilitation program

^c^We considered this to be a cohort study in the GRADE assessment because only one study arm was eligible for inclusion or because both study arms received a different version of the same intervention

### Risk of bias

Of the 27 cohort studies assessed with the CASP cohort study checklist, 14 had a good overall score (i.e., 7 or 8), 12 had a moderate score (i.e., 4–6), and one study had a low score (i.e., ≤ 3). Of the 13 RCTs assessed with the Cochrane Risk of Bias Assessment Tool 2.0, two had a low risk of bias, three had some concerns, and eight had a high risk of bias. Full details on the risk of bias assessment can be found in Additional file 2.

### Effects of rehabilitation interventions

**Fig. 2:**
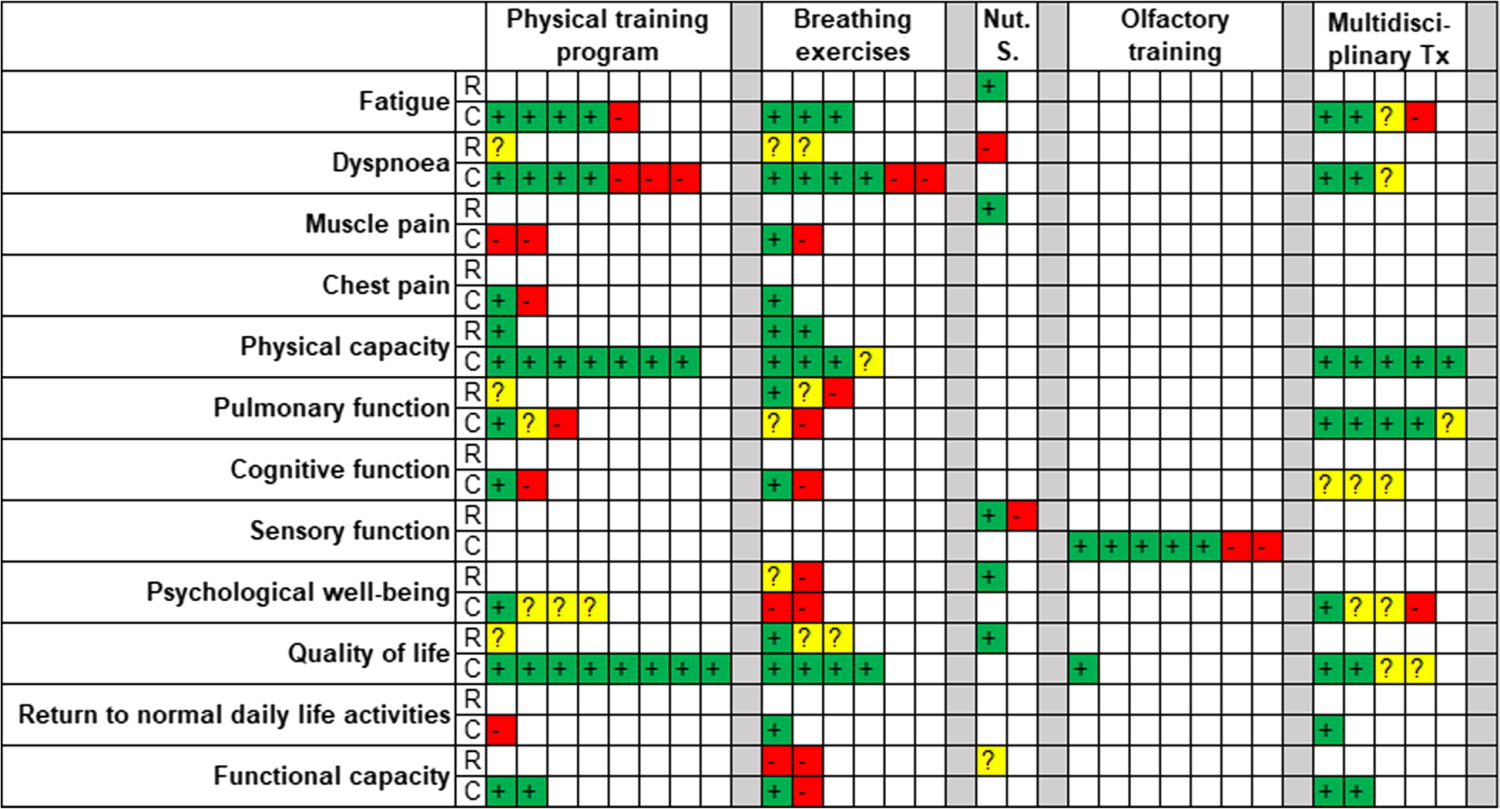
Graphical summary of the results of the included studies. Nut. S. = nutritional supplements, Tx = treatment, + (green) = beneficial results, ? (yellow) = mixed results, - (red) = no significant improvement, R = randomized controlled trial, C = cohort study

Figure 2 graphically summarises the results of the included studies. Full details and SoF tables can be found in Additional File 1.

#### Physical training program

Three RCTs [33, 41, 42] and 11 cohort studies [26–32, 38–40, 43] evaluated physical training as part of a rehabilitation program in patients with persisting complaints after COVID-19. Beneficial effects have been reported for the following outcomes (however, certainty of evidence is very low): dyspnoea, physical capacity, pulmonary function, quality of life, fatigue, chest pain, cognitive function, psychological well-being, and functional capacity. An improvement could not be demonstrated for muscle pain and return to work (very low certainty of evidence). One RCT [33] found that, in men with post-COVID-19 sarcopenia, low intensity training resulted in an increased handgrip strength and a better quality of life compared to high intensity training. Two studies [32, 41] reported adverse events, and when detected, none were related to the intervention.

#### Breathing exercises

Five RCTs [35–37, 41, 42] and five cohort studies [34, 38–40, 43] evaluated breathing exercises as part of a rehabilitation program in patients with prolonged symptoms after COVID-19. Beneficial effects (all with very low certainty of evidence) have been reported for the following outcomes: dyspnoea, physical capacity, pulmonary function, psychological well-being, quality of life, functional capacity, fatigue, muscle pain, chest pain, cognitive function, and return to normal daily life activities. Two studies reported adverse events such as hospitalisations [41] and dizziness [36]. One study reported that participation could trigger exacerbation of symptoms such as fatigue [36].

#### Nutritional supplements

Four RCTs assessed the effects of nutritional supplements in patients with persisting symptoms after COVID-19. The following supplements were evaluated: palmitoylethanolamide and luteolin (two studies [44, 45]), systemic enzymes (ImmunoSEB) and probiotics (one study [46]), and acetyl-carnitine (one study [42]).

Beneficial effects have been reported for the following outcomes (all with very low certainty of evidence): fatigue (systemic enzymes and probiotics), dyspnoea (acetyl-carnitine), muscle pain (acetyl-carnitine), sensory function (palmitoylethanolamide and luteolin), psychological well-being (acetyl-carnitine), quality of life (acetyl-carnitine), and functional capacity (acetyl-carnitine, mixed results). Two studies [45, 46] reported on adverse events, of which none were detected.

#### Olfactory training

Four RCTs [44, 45, 47, 50] and three cohort studies [48, 49, 51] evaluated olfactory training in patients with persisting olfactory complaints after COVID-19. For the RCTs, we only used control group data, so these were all considered as observational studies. Five studies [44, 47, 48, 50, 51] reported beneficial results on olfactory function, while two studies [45, 49] reported no difference (very low certainty of evidence). One study [51] reported beneficial effects on quality of life (very low certainty of evidence). One RCT [50] suggested that advanced olfactory training (i.e., increasing the number of essences) does not show superiority over classical olfactory training. Two studies reported adverse events: one study [50] observed that 18 out of 80 participants had side effects with olfactory training, while the other study [45] did not detect any adverse effect.

#### Multidisciplinary treatment

Five cohort studies [52–57] evaluated the effects of multidisciplinary treatment in patients with persisting complaints after COVID-19. All five studies included physiotherapy, four included psychological support, four included nutritional counselling and three included occupational therapy. Beneficial effects have been reported for the following outcomes (all with very low certainty of evidence): fatigue, dyspnoea, physical capacity, pulmonary function, cognitive function, psychological well-being, quality of life, return to work, and functional capacity. Two studies [52, 53, 56] reported adverse events and none were detected.

#### Other interventions

Three RCTs and four cohort studies investigated other interventions in patients with persisting symptoms after COVID-19. Narrative exposure therapy can have a positive effect on post-traumatic stress symptoms (one RCT [59], very low certainty of evidence). Aromatherapy can improve energy levels among women who are experiencing fatigue after recovering from COVID-19 (one RCT [60], low certainty of evidence). Hydrogen inhalation may increase the tolerance to physical activity (one RCT [64], very low certainty of evidence). Massage techniques may reduce fatigue and may improve cognitive function and psychological well-being (two cohort studies [58, 61], very low certainty of evidence). Hyperbaric oxygen therapy can have beneficial effects on fatigue (one cohort study [62], very low certainty of evidence). Enhanced external counterpulsation may reduce fatigue and brain fog and may improve physical capacity, psychological well-being, and functional capacity (one cohort study [63], very low certainty of evidence). Adverse events were reported for the following interventions: aromatherapy (one participant experienced headache; no other adverse events), hydrogen inhalation (none were detected), and hyperbaric oxygen therapy (none were detected).

## Discussion

### Main findings

Physical training programs and breathing exercises may reduce fatigue, dyspnoea, and chest pain and may improve physical capacity and quality of life, but the supporting evidence is very weak. Their effects on muscle pain, pulmonary function, cognitive function, psychological well-being, return to normal daily life activities, and functional capacity are still unclear. The evidence underpinning the effect of nutritional supplements on fatigue, dyspnoea, muscle pain, sensory function, psychological well-being, quality of life, and functional capacity is considered to be very poor. Also, the evidence is very uncertain about the effect of olfactory training on sensory function and quality of life. Multidisciplinary treatment may have beneficial effects on fatigue, dyspnoea, physical capacity, pulmonary function, quality of life, return to normal daily life activities, and functional capacity, but the evidence is very uncertain. Its effect on cognitive function and psychological well-being is still unclear. The certainty of evidence is very low due to study limitations (the majority of studies have a cohort design), inconsistency (e.g., some studies found positive results while others showed negative or mixed results), indirectness (e.g., physical training programs and breathing exercises were often evaluated simultaneously), and imprecision (low number of participants). For instance, some rehabilitation programs consisted of a combination of exercises (e.g., aerobic and strength training, with or without breathing techniques), so we are unable to derive from these data the most effective type of training.

### Strengths and limitations

A key strength of this systematic review is a rigorous methodology, including an extensive search and the use of the GRADE framework [24, 25] to assess the certainty of evidence, which was not performed in similar reviews [65–69]. Also, various ambulatory care professionals (i.e., general practitioners, physiotherapists, occupational therapists, psychologists, and dieticians) as well as patients with persisting symptoms after COVID-19 were involved in the literature search, which allowed them, from the perspective of their discipline and/or experience, to verify the list of retrieved publications and to add missing ones. Further, the use of existing COVID-19 libraries limited the chance that relevant literature was not identified.

However, this review has some limitations. Given persistent symptoms after COVID-19 is a relatively new condition, the majority of included studies are single-group cohort studies with a short follow-up period, which had an impact on the certainty of evidence. Additionally, the range of outcomes for the same intervention as well as the heterogeneity in interventions might limit the generalizability of our findings. We were also not able to summarize the results quantitatively. Moreover, this review did not consider the effect of the interventions on some other important symptoms that are commonly reported by these patients, such as general pain or discomfort, affected sleep, impaired walking, joint pain, and cough, with heterogeneity in number and severity experienced [5]. Last, few studies included in this review reported on the side effects or adverse events with the therapies applied. Reports on post exertional malaise or post exertional symptom exacerbation after (exercise) training have been made available and this may require tapering down or even stopping of the training program [70–72]. Further research is needed on the place of other interventions (such as occupational therapy) in this context [73].

### Comparison to existing literature

Previous reviews on rehabilitation interventions for patients with persisting symptoms after COVID-19 focussed on pulmonary rehabilitation [65] or only included RCTs [66]. Other reviews covered all or other (i.e., earlier) stages of rehabilitation of COVID-19 patients [67–69]. Their evidence suggests that, in line with our results, physical training and breathing exercises can be useful in patients with persisting symptoms after COVID-19. A review by Décary et al. summarized the current literature on care models and pathways for post COVID-19 condition and advises that rehabilitation care for these patients should include multiple professionals at different levels [74], which is in accordance with our results on the effects of multidisciplinary treatment. Since the closing of this review at least two RCTs were published. One RCT reported significant improvements in physical fitness, fatigue, quality of life, and symptoms of depression for using an 8-week supervised exercise training program compared to the WHO self-management brochure [75] in patients with mild initial COVID-19 [76]. The other RCT investigated the effect of respiratory muscle training and confirmed its effects on improvements of dyspnoea [77]. These newer studies provide further support to our findings.

### Implications for clinical practice and further research

These findings can guide ambulatory care practitioners for treating patients with persisting symptoms after COVID-19. This evidence should therefore be incorporated in clinical practice guidelines for the care of these patients, as already partially implemented in England [2], Germany [78], the Netherlands [79] and Belgium [80].

Given the paucity of evidence, high-quality and rigorous studies are needed to confirm our hypotheses. Future studies preferably have a controlled design and should include a sufficient number of participants. Also, adverse events of rehabilitation programs in ambulatory care must be studied more. Moreover, evidence is particularly scarce for the following interventions: advice on how to self-manage symptoms and on return to activities, occupational therapy, rehabilitation for communication and swallowing difficulties, cognitive training, and psychological interventions including coping and post-traumatic stress management strategies. Besides, standardized structured questionnaires specific to qualitative olfactory dysfunction should be routinely used, since the Sniffin’ Sticks test, which was mainly used in the olfactory training studies included in this review [44, 45, 47–51], focuses on quantitative loss of olfactory function and therefore can underestimate the prevalence of persistent COVID-related parosmia [81]. Finally, research into the prevalence, risk factors, and pathophysiology of persisting symptoms after COVID-19 will give more insight into which rehabilitation interventions may be most beneficial in this population, along with a tailored treatment according to the subtype of the condition.

## Conclusions

Physical training programs, breathing exercises, olfactory training, and multidisciplinary treatment can be effective rehabilitation therapies for patients with persisting symptoms after COVID-19. They have already shown marked effects on fatigue, dyspnoea, physical capacity, and quality of life. However, the certainty of evidence is very low, which means there is still a lot of uncertainty about these effects.

## Supplementary Information

**Additional file 1.**

**Additional file 2.**

## Data Availability

The datasets used and/or analysed during the current study are available from the corresponding author on reasonable request.

## Abbreviations

COVID-19: Coronavirus disease 2019
GRADE: Grading of Recommendation Assessment, Development, and Evaluation
RCT: Randomized controlled trial
SoF: Summary of Findings
WHO: World Health Organisation
