# Supplementary material for: Clinical effectiveness of rehabilitation in ambulatory care for patients with persisting symptoms after COVID-19: a systematic review
Source: BMC Infect Dis. 2023 Jun 21;23:419. doi: 10.1186/s12879-023-08374-x (PMC10283248; doi:10.1186/s12879-023-08374-x)
Supplement: Supplementary file 1 — Additional file 1. [file 12879_2023_8374_MOESM1_ESM.docx]

**Clinical effectiveness of rehabilitation in ambulatory care for patients with persisting symptoms after COVID-19: a systematic review (Additional File 1)**

Table of contents:

[Preferred Reporting Items for Systematic Reviews and Meta-Analyses (PRISMA) 2020 Checklist 2](#_Toc124763572)

[PRISMA 2020 for Abstracts Checklist 7](#_Toc124763573)

[Search strings for all databases 9](#_Toc124763574)

[Research Aid Networks Long Covid Library 9](#_Toc124763575)

[Resources LongCovid 9](#_Toc124763576)

[Cochrane COVID-19 Study Register 9](#_Toc124763577)

[Epistemonikos 11](#_Toc124763578)

[World Health Organisation COVID-19 database 11](#_Toc124763579)

[Embase 12](#_Toc124763580)

[MEDLINE 18](#_Toc124763581)

[Cochrane Library 25](#_Toc124763582)

[Web of Science Core Collection 32](#_Toc124763583)

[Cumulative Index to Nursing and Allied Health Literature (CINAHL) 39](#_Toc124763584)

[PsycArticles 61](#_Toc124763585)

[PEDro 62](#_Toc124763586)

[EuropePMC 62](#_Toc124763587)

[Summary of Findings tables 63](#_Toc124763588)

[Physical training program 63](#_Toc124763589)

[Breathing exercises 66](#_Toc124763590)

[Nutritional supplements 71](#_Toc124763591)

[Olfactory training 73](#_Toc124763592)

[Multidisciplinary treatment 74](#_Toc124763593)

[Other interventions 77](#_Toc124763594)

[List of abbreviations 81](#_Toc124763595)

# Preferred Reporting Items for Systematic Reviews and Meta-Analyses (PRISMA) 2020 Checklist

| **Section and Topic** | **Item #** | **Checklist item** | **Location where item is reported** |
| --- | --- | --- | --- |
| **TITLE** | | |  |
| Title | 1 | Identify the report as a systematic review. | Manuscript page 1 |
| **ABSTRACT** | | |  |
| Abstract | 2 | See the PRISMA 2020 for Abstracts checklist. | Manuscript page 3-4 |
| **INTRODUCTION** | | |  |
| Rationale | 3 | Describe the rationale for the review in the context of existing knowledge. | Manuscript page 6-7 |
| Objectives | 4 | Provide an explicit statement of the objective(s) or question(s) the review addresses. | Manuscript page 7 |
| **METHODS** | | |  |
| Eligibility criteria | 5 | Specify the inclusion and exclusion criteria for the review and how studies were grouped for the syntheses. | Manuscript page 8 |
| Information sources | 6 | Specify all databases, registers, websites, organisations, reference lists and other sources searched or consulted to identify studies. Specify the date when each source was last searched or consulted. | Manuscript page 7 |
| Search strategy | 7 | Present the full search strategies for all databases, registers and websites, including any filters and limits used. | Additional File 1 page 9-62 |
| Selection process | 8 | Specify the methods used to decide whether a study met the inclusion criteria of the review, including how many reviewers screened each record and each report retrieved, whether they worked independently, and if applicable, details of automation tools used in the process. | Manuscript page 8-9 |
| Data collection process | 9 | Specify the methods used to collect data from reports, including how many reviewers collected data from each report, whether they worked independently, any processes for obtaining or confirming data from study investigators, and if applicable, details of automation tools used in the process. | Manuscript page 9 |
| Data items | 10a | List and define all outcomes for which data were sought. Specify whether all results that were compatible with each outcome domain in each study were sought (e.g. for all measures, time points, analyses), and if not, the methods used to decide which results to collect. | Manuscript page 9 |
|  | 10b | List and define all other variables for which data were sought (e.g. participant and intervention characteristics, funding sources). Describe any assumptions made about any missing or unclear information. | Manuscript page 9 |
| Study risk of bias assessment | 11 | Specify the methods used to assess risk of bias in the included studies, including details of the tool(s) used, how many reviewers assessed each study and whether they worked independently, and if applicable, details of automation tools used in the process. | Manuscript page 9 |
| Effect measures | 12 | Specify for each outcome the effect measure(s) (e.g. risk ratio, mean difference) used in the synthesis or presentation of results. | Manuscript page 10 |
| Synthesis methods | 13a | Describe the processes used to decide which studies were eligible for each synthesis (e.g. tabulating the study intervention characteristics and comparing against the planned groups for each synthesis (item #5)). | Manuscript page 10 |
|  | 13b | Describe any methods required to prepare the data for presentation or synthesis, such as handling of missing summary statistics, or data conversions. | Manuscript page 10 |
|  | 13c | Describe any methods used to tabulate or visually display results of individual studies and syntheses. | Manuscript page 10 |
|  | 13d | Describe any methods used to synthesize results and provide a rationale for the choice(s). If meta-analysis was performed, describe the model(s), method(s) to identify the presence and extent of statistical heterogeneity, and software package(s) used. | Manuscript page 10 |
|  | 13e | Describe any methods used to explore possible causes of heterogeneity among study results (e.g. subgroup analysis, meta-regression). | Not applicable |
|  | 13f | Describe any sensitivity analyses conducted to assess robustness of the synthesized results. | Not applicable |
| Reporting bias assessment | 14 | Describe any methods used to assess risk of bias due to missing results in a synthesis (arising from reporting biases). | Not applicable |
| Certainty assessment | 15 | Describe any methods used to assess certainty (or confidence) in the body of evidence for an outcome. | Manuscript page 10 |
| **RESULTS** | | |  |
| Study selection | 16a | Describe the results of the search and selection process, from the number of records identified in the search to the number of studies included in the review, ideally using a flow diagram. | Manuscript page 10 |
|  | 16b | Cite studies that might appear to meet the inclusion criteria, but which were excluded, and explain why they were excluded. | Manuscript page 10 |
| Study characteristics | 17 | Cite each included study and present its characteristics. | Manuscript page 11-13 |
| Risk of bias in studies | 18 | Present assessments of risk of bias for each included study. | Manuscript page 13; Additional File 2 |
| Results of individual studies | 19 | For all outcomes, present, for each study: (a) summary statistics for each group (where appropriate) and (b) an effect estimate and its precision (e.g. confidence/credible interval), ideally using structured tables or plots. | Not applicable |
| Results of syntheses | 20a | For each synthesis, briefly summarise the characteristics and risk of bias among contributing studies. | Manuscript page 13-16; Additional File 1 page 63-81 |
|  | 20b | Present results of all statistical syntheses conducted. If meta-analysis was done, present for each the summary estimate and its precision (e.g. confidence/credible interval) and measures of statistical heterogeneity. If comparing groups, describe the direction of the effect. | Additional File 1 page 63-81 |
|  | 20c | Present results of all investigations of possible causes of heterogeneity among study results. | Not applicable |
|  | 20d | Present results of all sensitivity analyses conducted to assess the robustness of the synthesized results. | Not applicable |
| Reporting biases | 21 | Present assessments of risk of bias due to missing results (arising from reporting biases) for each synthesis assessed. | Not applicable |
| Certainty of evidence | 22 | Present assessments of certainty (or confidence) in the body of evidence for each outcome assessed. | Manuscript page 13-16; Additional File 1 page 63-81 |
| **DISCUSSION** | | |  |
| Discussion | 23a | Provide a general interpretation of the results in the context of other evidence. | Manuscript page 16-19 |
|  | 23b | Discuss any limitations of the evidence included in the review. | Manuscript page 17-18 |
|  | 23c | Discuss any limitations of the review processes used. | Manuscript page 17-18 |
|  | 23d | Discuss implications of the results for practice, policy, and future research. | Manuscript page 19 |
| **OTHER INFORMATION** | | |  |
| Registration and protocol | 24a | Provide registration information for the review, including register name and registration number, or state that the review was not registered. | Manuscript page 10 |
|  | 24b | Indicate where the review protocol can be accessed, or state that a protocol was not prepared. | Manuscript page 10 |
|  | 24c | Describe and explain any amendments to information provided at registration or in the protocol. | Not applicable |
| Support | 25 | Describe sources of financial or non-financial support for the review, and the role of the funders or sponsors in the review. | Manuscript page 20-21 |
| Competing interests | 26 | Declare any competing interests of review authors. | Manuscript page 20 |
| Availability of data, code and other materials | 27 | Report which of the following are publicly available and where they can be found: template data collection forms; data extracted from included studies; data used for all analyses; analytic code; any other materials used in the review. | Not applicable |

From: Page MJ, McKenzie JE, Bossuyt PM, Boutron I, Hoffmann TC, Mulrow CD, et al. The PRISMA 2020 statement: an updated guideline for reporting systematic reviews. BMJ 2021;372:n71. doi: 10.1136/bmj.n71

For more information, visit: <http://www.prisma-statement.org/>

# PRISMA 2020 for Abstracts Checklist

| **Section and Topic** | **Item #** | **Checklist item** | **Reported (Yes/No)** |
| --- | --- | --- | --- |
| **TITLE** | | |  |
| Title | 1 | Identify the report as a systematic review. | Yes |
| **BACKGROUND** | | |  |
| Objectives | 2 | Provide an explicit statement of the main objective(s) or question(s) the review addresses. | Yes |
| **METHODS** | | |  |
| Eligibility criteria | 3 | Specify the inclusion and exclusion criteria for the review. | Yes |
| Information sources | 4 | Specify the information sources (e.g. databases, registers) used to identify studies and the date when each was last searched. | Yes |
| Risk of bias | 5 | Specify the methods used to assess risk of bias in the included studies. | Yes |
| Synthesis of results | 6 | Specify the methods used to present and synthesise results. | Yes |
| **RESULTS** | | |  |
| Included studies | 7 | Give the total number of included studies and participants and summarise relevant characteristics of studies. | Yes |
| Synthesis of results | 8 | Present results for main outcomes, preferably indicating the number of included studies and participants for each. If meta-analysis was done, report the summary estimate and confidence/credible interval. If comparing groups, indicate the direction of the effect (i.e. which group is favoured). | Yes |
| **DISCUSSION** | | |  |
| Limitations of evidence | 9 | Provide a brief summary of the limitations of the evidence included in the review (e.g. study risk of bias, inconsistency and imprecision). | Yes |
| Interpretation | 10 | Provide a general interpretation of the results and important implications. | Yes |
| **OTHER** | | |  |
| Funding | 11 | Specify the primary source of funding for the review. | Yes |
| Registration | 12 | Provide the register name and registration number. | Yes |

From: Page MJ, McKenzie JE, Bossuyt PM, Boutron I, Hoffmann TC, Mulrow CD, et al. The PRISMA 2020 statement: an updated guideline for reporting systematic reviews. BMJ 2021;372:n71. doi: 10.1136/bmj.n71

For more information, visit: http://www.prisma-statement.org/

# Search strings for all databases

## Research Aid Networks Long Covid Library

| URL | https://www.zotero.org/groups/4411227/research_aid_networks_long_covid_library/items/9ITDFM6W |
| --- | --- |
| Search date | 09/05/2022 |
| How the results were selected | Downloaded the records as a RIS file and imported them into Endnote. |
| No. of results | 654 |

## Resources LongCovid

| URL | https://docs.google.com/spreadsheets/d/1jy354stmCE30zYoE5Ou3lz0O1hZSbvuLfvxcUGoBroQ/htmlview?fbclid=IwAR1RFFF7YnmeNzGQIH9uf3RcgWzmHkRARzj_oIfu4F00qGiHLe4yH0bP3L4 |
| --- | --- |
| Search date | 09/05/2022 |
| How the results were selected | Only screened the records of the tabblad “Care”. |
| No. of results | 141 |

## Cochrane COVID-19 Study Register

| URL | https://covid-19.cochrane.org/ |
| --- | --- |
| Search date | 09/05/2022 |
| How the results were selected | Ran a series of separate searches and downloaded a RIS file for every search. Only included studies with results. Only included journal articles and preprint articles. |
| Search strategies | Search 1 – 183 results  longcovid* or "long covid*" or longcoronavirus* or longcoronavirinae or "long coronavirinae" or longCov or "long Cov" or longsars or "long sars"  Search 2 – 521 results  long severe acute respiratory syndrome  Search 3 – 183 results  Sequelae  Search 4 – 108 results  nonrecover* or "non recover*" or subacute* or "sub acute*" or postacute* or "post acute*" or postdischarg* or "post discharg*" or postinfect* or "post infect*" or postviral* or "post viral*" or postvirus* or "post virus*"  Search 5 – 130 results  postcovid* or "post covid*" or postcoronavirus* or postcoronavirinae or "post coronavirinae" or postCov or "post Cov" or postsars or "post sars"  Search 6 – 34 results  ongoing symptom or persistent symptom  Search 7 – 2 results  "after care" or aftercare  Search 8 – 189 results  rehabilitation or recuperation or convalescence or recovery AND ongoing  Search 9 – 41 results  "daily activities"  Search 10 – 7 results  "functional status" |
| No. of results | 1398 |
| No. of results after deduplication | 838 |

## Epistemonikos

| URL | https://app.iloveevidence.com/loves/5e6fdb9669c00e4ac072701d/advanced-search |
| --- | --- |
| Search date | 09/05/2022 |
| How the results were selected | Ran a series of separate searches and downloaded a RIS file for every search. Included broad syntheses, systematic reviews and primary studies reporting data. |
| Search strategies | Search 1 – 987 results  longcovid* or "long covid*" or longcoronavirus* or longcoronavirinae or "long coronavirinae" or longCov or "long Cov" or longsars or "long sars" or "long severe acute respiratory syndrome" or postcovid* or "post covid*" or postcoronavirus* or postcoronavirinae or "post coronavirinae" or postCov or "post Cov" or postsars or "post sars" or "functional status" or "after care" or aftercare  Search 2 – 108 results  rehabilitation or recuperation or convalescence or recovery or complication or sequelae AND ongoing  Search 3 – 43 results  "ongoing symptom" or "persistent symptom"  Search 4 – 242 results  postcovid* or "post covid*" or postcoronavirus* or postcoronavirinae or "post coronavirinae" or postCov or "post Cov" or postsars or "post sars" |
| No. of results | 1380 |
| No. of results after deduplication | 1046 |

## World Health Organisation COVID-19 database

| URL | https://search.bvsalud.org/global-literature-on-novel-coronavirus-2019-ncov/ |
| --- | --- |
| Search date | 09/05/2022 |
| How the results were selected | Ran a series of 6 separate searches and downloaded a RIS file for every search. Only searched in the title. Only included articles and preprints. |
| Search strategies | Search 1 – 5850 results  longcovid* or "long covid*" or longcoronavirus* or longcoronavirinae or "long coronavirinae" or longCov or "long Cov" or longsars or "long sars" or "long severe acute respiratory syndrome" or postcovid* or "post covid*" or postcoronavirus* or postcoronavirinae or "post coronavirinae" or postCov or "post Cov" or postsars or "post sars" or "daily activities" or "functional status" or "after care" or aftercare  Search 2 - 1024 results  nonrecover* or "non recover*" or subacute* or "sub acute*" or postacute* or "post acute*" or postdischarg* or "post discharg*" or postinfect* or "post infect*" or postviral* or "post viral*" or postvirus* or "post virus*"    Search 3 – 579 results  sequelae  Search 4 - 10 results  (ongoing or persistent) and symptom  Search 5 - 10 results  (rehabilitation or recuperation or convalescence or recovery or complication) and ongoing  Search 6 - 4796 results  postcovid* or "post covid*" or postcoronavirus* or postcoronavirinae or "post coronavirinae" or postCov or "post Cov" or postsars or "post sars" |
| No. of results | 12269 |
| No. of results after deduplication | 6755 |

## Embase

| Platform | Elsevier  Advanced search  No mapping options |
| --- | --- |
| Search date | 09/05/2022 |
| No. of results | 12342 |

|  |  | No. of results |
| --- | --- | --- |
| #1 | (longcovid* or ‘long covid*’ or longcoronavirus* or ‘longcorona* virus*’ or ‘long coronavirus*’ or ‘long corona* virus*’ or longcoronovirus* or ‘longcorono* virus*’ or ‘long coronovirus*’ or ‘long corono* virus*’ or longcoronavirinae* or ‘longcorona* virinae*’ or ‘long coronavirinae*’ or ‘long corona* virinae*’ or longCov or ‘long Cov’ or longsars* or ‘long sars*’ or ‘long severe acute respiratory syndrome*’ or longncov* or ‘long ncov*’ or longhcov* or ‘long hcov*’):ti,ab,kw | 1277 |
| #2 | ((long* or endur* or legacy* or slow* or gradual* or protract* or lengthy* or chronic* or persist* or relaps* or remit* or remission* or residual* or delay* or prolong* or extend* or linger* or permanent* or fluctuat* or sequela* or multisystem* or ‘multi system*’ or nonrecover* or ‘non recover*’ or subacute* or ‘sub acute*’ or lasting* or continuous* or continual* or continuing* or postacute* or ‘post acute*’ or postdischarg* or ‘post discharg*’ or postinfect* or ‘post infect*’ or postviral* or ‘post viral*’ or postvirus* or ‘post virus*’) NEAR/1 (covid* or coronavirus* or ‘corona* virus*’ or coronovirus* or ‘corono* virus*’ or coronavirinae* or ‘corona* virinae*’ or Cov or ‘2019-nCoV*’ or 2019nCoV* or ‘19-nCoV*’ or 19nCoV* or nCoV2019* or ‘nCoV-2019*’ or nCoV19* or ‘nCoV-19*’ or ‘HCoV-19*’ or HCoV19* or ‘HCoV-2019*’ or HCoV2019* or ‘2019 novel*’ or Ncov* or ‘n-cov’ or ‘SARS-CoV-2*’ or ‘SARSCoV-2*’ or ‘SARSCoV2*’ or ‘SARS-CoV2*’ or SARSCov19* or ‘SARS-Cov19*’ or ‘SARSCov-19*’ or ‘SARS-Cov-19*’ or SARSCov2019* or ‘SARS-Cov2019*’ or ‘SARSCov-2019*’ or ‘SARS-Cov-2019*’ or SARS2* or ‘SARS-2*’ or SARScoronavirus2* or ‘SARS-coronavirus-2*’ or ‘SARScoronavirus 2*’ or ‘SARS coronavirus2*’ or SARScoronovirus2* or ‘SARS-coronovirus-2*’ or ‘SARScoronovirus 2*’ or ‘SARS coronovirus2*’ or ‘severe acute respiratory syndrome*’)):ti,ab | 2297 |
| #3 | ((‘long* term*’ or longterm* or ‘long* haul*’ or longhaul* or ‘long* tail*’ or longtail* or longduration* or ‘long duration*’ or longlast* or ‘long last*’ or longstanding* or ‘long standing*’ or ‘medium* term*’ or mediumterm*) NEAR/3 (covid* or coronavirus* or ‘corona* virus*’ or coronovirus* or ‘corono* virus*’ or coronavirinae* or ‘corona* virinae*’ or Cov or ‘2019-nCoV*’ or 2019nCoV* or ‘19-nCoV*’ or 19nCoV* or nCoV2019* or ‘nCoV-2019*’ or nCoV19* or ‘nCoV-19*’ or ‘HCoV-19*’ or HCoV19* or ‘HCoV-2019*’ or HCoV2019* or ‘2019 novel*’ or Ncov* or ‘n-cov’ or ‘SARS-CoV-2*’ or ‘SARSCoV-2*’ or ‘SARSCoV2*’ or ‘SARS-CoV2*’ or SARSCov19* or ‘SARS-Cov19*’ or ‘SARSCov-19*’ or ‘SARS-Cov-19*’ or SARSCov2019* or ‘SARS-Cov2019*’ or ‘SARSCov-2019*’ or ‘SARS-Cov-2019*’ or SARS2* or ‘SARS-2*’ or SARScoronavirus2* or ‘SARS-coronavirus-2*’ or ‘SARScoronavirus 2*’ or ‘SARS coronavirus2*’ or SARScoronovirus2* or ‘SARS-coronovirus-2*’ or ‘SARScoronovirus 2*’ or ‘SARS coronovirus2*’ or ‘severe acute respiratory syndrome*’)):ti,ab | 1506 |
| #4 | ((postcovid* or ‘post covid*’ or postcoronavirus* or ‘postcorona* virus*’ or ‘post coronavirus*’ or ‘post corona* virus*’ or postcoronovirus* or ‘postcorono* virus*’ or ‘post coronovirus*’ or ‘post corono* virus*’ or postcoronavirinae* or ‘postcorona* virinae*’ or ‘post coronavirinae*’ or ‘post corona* virinae*’ or postCov or ‘post Cov’ or postsars* or ‘post sars*’ or ‘post severe acute respiratory syndrome*’ or postncov* or ‘post ncov*’ or posthcov* or ‘post hcov*’) NEAR/3 (syndrome* or disorder* or illness* or sickness* or disease* or condition* or symptom* or sign* or prognos* or followup* or ‘follow up*’ or feature* or comorbid* or ‘co morbid*’ or multimorbid* or ‘multi morbid*’ or survivor* or survival* or risk* or care* or convalescen* or recuperat* or aftercare* or ambulatory* or outpatient* or ‘out patient*’)):ti,ab | 1320 |
| #5 | ((ongoing* or long* or endur* or legacy* or slow* or gradual* or protract* or lengthy* or chronic* or persist* or relaps* or remit* or remission* or residual* or delay* or prolong* or extend* or linger* or permanent* or fluctuat* or multisystem* or ‘multi system*’ or nonrecover* or ‘non recover*’ or subacute* or ‘sub acute*’ or lasting* or continuous* or continual* or continuing* or postacute* or ‘post acute*’ or postdischarg* or ‘post discharg*’ or postinfect* or ‘post infect*’ or postviral* or ‘post viral*’ or postvirus* or ‘post virus*’ or ‘medium* term*’ or mediumterm*) NEAR/4 (sequela* or illness* or symptom* or sign* or prognos* or rehab* or convalescen* or recuperat* or followup* or ‘follow up*’ or feature*) NEAR/6 (covid* or coronavirus* or ‘corona* virus*’ or coronovirus* or ‘corono* virus*’ or coronavirinae* or ‘corona* virinae*’ or Cov or ‘2019-nCoV*’ or 2019nCoV* or ‘19-nCoV*’ or 19nCoV* or nCoV2019* or ‘nCoV-2019*’ or nCoV19* or ‘nCoV-19*’ or ‘HCoV-19*’ or HCoV19* or ‘HCoV-2019*’ or HCoV2019* or ‘2019 novel*’ or Ncov* or ‘n-cov’ or ‘SARS-CoV-2*’ or ‘SARSCoV-2*’ or ‘SARSCoV2*’ or ‘SARS-CoV2*’ or SARSCov19* or ‘SARS-Cov19*’ or ‘SARSCov-19*’ or ‘SARS-Cov-19*’ or SARSCov2019* or ‘SARS-Cov2019*’ or ‘SARSCov-2019*’ or ‘SARS-Cov-2019*’ or SARS2* or ‘SARS-2*’ or SARScoronavirus2* or ‘SARS-coronavirus-2*’ or ‘SARScoronavirus 2*’ or ‘SARS coronavirus2*’ or SARScoronovirus2* or ‘SARS-coronovirus-2*’ or ‘SARScoronovirus 2*’ or ‘SARS coronovirus2*’ or ‘severe acute respiratory syndrome*’)):ti,ab | 2652 |
| #6 | ((ongoing* or long* or endur* or legacy* or slow* or gradual* or protract* or lengthy* or chronic* or persist* or relaps* or remit* or remission* or residual* or delay* or prolong* or extend* or linger* or permanent* or fluctuat* or multisystem* or ‘multi system*’ or subacute* or ‘sub acute*’ or lasting* or continuous* or continual* or continuing* or post* or after* or follow* or ‘medium* term*’ or mediumterm*) NEAR/1 recover* NEAR/6 (covid* or coronavirus* or ‘corona* virus*’ or coronovirus* or ‘corono* virus*’ or coronavirinae* or ‘corona* virinae*’ or Cov or ‘2019-nCoV*’ or 2019nCoV* or ‘19-nCoV*’ or 19nCoV* or nCoV2019* or ‘nCoV-2019*’ or nCoV19* or ‘nCoV-19*’ or ‘HCoV-19*’ or HCoV19* or ‘HCoV-2019*’ or HCoV2019* or ‘2019 novel*’ or Ncov* or ‘n-cov’ or ‘SARS-CoV-2*’ or ‘SARSCoV-2*’ or ‘SARSCoV2*’ or ‘SARS-CoV2*’ or SARSCov19* or ‘SARS-Cov19*’ or ‘SARSCov-19*’ or ‘SARS-Cov-19*’ or SARSCov2019* or ‘SARS-Cov2019*’ or ‘SARSCov-2019*’ or ‘SARS-Cov-2019*’ or SARS2* or ‘SARS-2*’ or SARScoronavirus2* or ‘SARS-coronavirus-2*’ or ‘SARScoronavirus 2*’ or ‘SARS coronavirus2*’ or SARScoronovirus2* or ‘SARS-coronovirus-2*’ or ‘SARScoronovirus 2*’ or ‘SARS coronovirus2*’ or ‘severe acute respiratory syndrome*’)):ti,ab | 1092 |
| #7 | #1 OR #2 OR #3 OR #4 OR #5 OR #6 | 6765 |
| #8 | Coronavirinae/exp | 92952 |
| #9 | ‘Coronavirus infection’/exp | 233126 |
| #10 | ‘coronavirus disease 2019’/exp or ‘severe acute respiratory syndrome coronavirus 2’/exp | 226763 |
| #11 | (covid* or coronavirus* or ‘corona* virus*’ or coronovirus* or ‘corono* virus*’ or coronavirinae* or ‘corona* virinae*’ or Cov or ‘2019-nCoV*’ or 2019nCoV* or ‘19-nCoV*’ or 19nCoV* or nCoV2019* or ‘nCoV-2019*’ or nCoV19* or ‘nCoV-19*’ or ‘HCoV-19*’ or HCoV19* or ‘HCoV-2019*’ or HCoV2019* or ‘2019 novel*’ or Ncov* or ‘n-cov’ or ‘SARS-CoV-2*’ or ‘SARSCoV-2*’ or ‘SARSCoV2*’ or ‘SARS-CoV2*’ or SARSCov19* or ‘SARS-Cov19*’ or ‘SARSCov-19*’ or ‘SARS-Cov-19*’ or SARSCov2019* or ‘SARS-Cov2019*’ or ‘SARSCov-2019*’ or ‘SARS-Cov-2019*’ or SARS2* or ‘SARS-2*’ or SARScoronavirus2* or ‘SARS-coronavirus-2*’ or ‘SARScoronavirus 2*’ or ‘SARS coronavirus2*’ or SARScoronovirus2* or ‘SARS-coronovirus-2*’ or ‘SARScoronovirus 2*’ or ‘SARS coronovirus2*’ or ‘severe acute respiratory syndrome*’):ti | 239217 |
| #12 | #8 OR #9 OR #10 OR #11 | 294008 |
| #13 | ‘Functional status’/de | 61558 |
| #14 | Aftercare/de | 8075 |
| #15 | Convalescence/de | 54898 |
| #16 | Rehabilitation/de | 96852 |
| #17 | ‘Rehabilitation care’/de | 19441 |
| #18 | ‘Daily life activity’/de | 103754 |
| #19 | ‘Physical performance’/de | 24418 |
| #20 | #13 OR #14 OR #15 OR #16 OR #17 OR #18 OR #19 | 348236 |
| #21 | #12 AND #20 | 5253 |
| #22 | (‘long* haul*’ or longhaul* or ‘long* tail*’ or longtail* or longduration* or ‘long duration*’ or longlast* or ‘long last*’ or longstanding* or ‘long standing*’):ti,ab AND (#8 OR #9 OR #10) | 1238 |
| #23 | ((recover* or nonrecover*) NEAR/3 function* NEAR/6 (covid* or coronavirus* or ‘corona* virus*’ or coronovirus* or ‘corono* virus*’ or coronavirinae* or ‘corona* virinae*’ or Cov or ‘2019-nCoV*’ or 2019nCoV* or ‘19-nCoV*’ or 19nCoV* or nCoV2019* or ‘nCoV-2019*’ or nCoV19* or ‘nCoV-19*’ or ‘HCoV-19*’ or HCoV19* or ‘HCoV-2019*’ or HCoV2019* or ‘2019 novel*’ or Ncov* or ‘n-cov’ or ‘SARS-CoV-2*’ or ‘SARSCoV-2*’ or ‘SARSCoV2*’ or ‘SARS-CoV2*’ or SARSCov19* or ‘SARS-Cov19*’ or ‘SARSCov-19*’ or ‘SARS-Cov-19*’ or SARSCov2019* or ‘SARS-Cov2019*’ or ‘SARSCov-2019*’ or ‘SARS-Cov-2019*’ or SARS2* or ‘SARS-2*’ or SARScoronavirus2* or ‘SARS-coronavirus-2*’ or ‘SARScoronavirus 2*’ or ‘SARS coronavirus2*’ or SARScoronovirus2* or ‘SARS-coronovirus-2*’ or ‘SARScoronovirus 2*’ or ‘SARS coronovirus2*’ or ‘severe acute respiratory syndrome*’)):ti,ab | 119 |
| #24 | ((postacute* or ‘post acute*’ or postdischarg* or ‘post discharg*’ or postinfect* or ‘post infect*’ or postviral* or ‘post viral*’ or postvirus* or ‘post virus*’ or subacute* or ‘sub acute*’) NEAR/3 (care* or convalescen* or recuperat* or aftercare* or ambulatory* or outpatient* or ‘out patient*’ or survivor* or survival*) NEAR/6 (covid* or coronavirus* or ‘corona* virus*’ or coronovirus* or ‘corono* virus*’ or coronavirinae* or ‘corona* virinae*’ or Cov or ‘2019-nCoV*’ or 2019nCoV* or ‘19-nCoV*’ or 19nCoV* or nCoV2019* or ‘nCoV-2019*’ or nCoV19* or ‘nCoV-19*’ or ‘HCoV-19*’ or HCoV19* or ‘HCoV-2019*’ or HCoV2019* or ‘2019 novel*’ or Ncov* or ‘n-cov’ or ‘SARS-CoV-2*’ or ‘SARSCoV-2*’ or ‘SARSCoV2*’ or ‘SARS-CoV2*’ or SARSCov19* or ‘SARS-Cov19*’ or ‘SARSCov-19*’ or ‘SARS-Cov-19*’ or SARSCov2019* or ‘SARS-Cov2019*’ or ‘SARSCov-2019*’ or ‘SARS-Cov-2019*’ or SARS2* or ‘SARS-2*’ or SARScoronavirus2* or ‘SARS-coronavirus-2*’ or ‘SARScoronavirus 2*’ or ‘SARS coronavirus2*’ or SARScoronovirus2* or ‘SARS-coronovirus-2*’ or ‘SARScoronovirus 2*’ or ‘SARS coronovirus2*’ or ‘severe acute respiratory syndrome*’)):ti,ab | 89 |
| #25 | ((convalescen* or recuperat* or after* or followup* or ‘follow up*’ or rehab*) NEAR/1 (therap* or care*) NEAR/6 (covid* or coronavirus* or ‘corona* virus*’ or coronovirus* or ‘corono* virus*’ or coronavirinae* or ‘corona* virinae*’ or Cov or ‘2019-nCoV*’ or 2019nCoV* or ‘19-nCoV*’ or 19nCoV* or nCoV2019* or ‘nCoV-2019*’ or nCoV19* or ‘nCoV-19*’ or ‘HCoV-19*’ or HCoV19* or ‘HCoV-2019*’ or HCoV2019* or ‘2019 novel*’ or Ncov* or ‘n-cov’ or ‘SARS-CoV-2*’ or ‘SARSCoV-2*’ or ‘SARSCoV2*’ or ‘SARS-CoV2*’ or SARSCov19* or ‘SARS-Cov19*’ or ‘SARSCov-19*’ or ‘SARS-Cov-19*’ or SARSCov2019* or ‘SARS-Cov2019*’ or ‘SARSCov-2019*’ or ‘SARS-Cov-2019*’ or SARS2* or ‘SARS-2*’ or SARScoronavirus2* or ‘SARS-coronavirus-2*’ or ‘SARScoronavirus 2*’ or ‘SARS coronavirus2*’ or SARScoronovirus2* or ‘SARS-coronovirus-2*’ or ‘SARScoronovirus 2*’ or ‘SARS coronovirus2*’ or ‘severe acute respiratory syndrome*’)):ti,ab | 510 |
| #26 | (covid* or coronavirus* or ‘corona* virus*’ or coronovirus* or ‘corono* virus*’ or coronavirinae* or ‘corona* virinae*’ or Cov or ‘2019-nCoV*’ or 2019nCoV* or ‘19-nCoV*’ or 19nCoV* or nCoV2019* or ‘nCoV-2019*’ or nCoV19* or ‘nCoV-19*’ or ‘HCoV-19*’ or HCoV19* or ‘HCoV-2019*’ or HCoV2019* or ‘2019 novel*’ or Ncov* or ‘n-cov’ or ‘SARS-CoV-2*’ or ‘SARSCoV-2*’ or ‘SARSCoV2*’ or ‘SARS-CoV2*’ or SARSCov19* or ‘SARS-Cov19*’ or ‘SARSCov-19*’ or ‘SARS-Cov-19*’ or SARSCov2019* or ‘SARS-Cov2019*’ or ‘SARSCov-2019*’ or ‘SARS-Cov-2019*’ or SARS2* or ‘SARS-2*’ or SARScoronavirus2* or ‘SARS-coronavirus-2*’ or ‘SARScoronavirus 2*’ or ‘SARS coronavirus2*’ or SARScoronovirus2* or ‘SARS-coronovirus-2*’ or ‘SARScoronovirus 2*’ or ‘SARS coronovirus2*’ or ‘severe acute respiratory syndrome*’):ti,ab | 280960 |
| #27 | ((ongoing* or endur* or long* or legacy* or slow* or gradual* or protract* or lengthy* or chronic* or persist* or relaps* or remit* or remission* or residual* or delay* or prolong* or extend* or linger* or permanent* or fluctuat* or multisystem* or ‘multi system*’ or nonrecover* or ‘non recover*’ or subacute* or ‘sub acute*’ or lasting* or continuous* or continual* or continuing* or postacute* or ‘post acute*’ or postdischarg* or ‘post discharg*’ or postinfect* or ‘post infect*’ or postviral* or ‘post viral*’ or postvirus* or ‘post virus*’ or ‘medium* term*’ or mediumterm*) NEAR/1 (sequela* or complication* or consequence* or effect* or event* or impact* or outcome* or reaction* or complexit* or aftercare* or impair* or problem* or issue* or symptom* or disorder*)):ti,ab | 192253 |
| #28 | #26 AND #27 | 2941 |
| #29 | ((physiolog* or neuro* or cardio* or gastro* or musculo* or renal* or kidney* or cognitive* or cognition* or rheumato* or dermatol* or skin* or haematol* or blood* or autonomic* or nervous* or ‘nervous system*’ or otolaryngol* or laryngol* or otolog* or cerebro* or brain* or vascular* or respirator* or lung* or pulmonary* or psycholog* or ‘mental health*’ or mental* or psychiatr* or exertion* or debilit* or devitali* or enervat* or drain* or sleep* or weak* or tired* or frail* or sapp* or strength* or confusion* or letharg* or fatigue* or tired* or weariness* or exhaust* or malaise* or pain* or headache* or breathless* or breathing* or myalgia* or delirious* or delirium* or appetite* or muscle* or muscular* or fitness* or memory* or stress* or depress* or anxiety* or emotion* or cough* or fever* or temperatur* or pneumon* or conjunctivit* or throat* or pharyngit* or dyspnea* or dyspnoea* or sick* or nausea* or nauseous* or vomit* or diarrhoea* or diarrhea* or taste* or anosmia* or smell* or olfact* or sweat* or dehydrat* or pyrexi* or nasal* or nose* or mucus* or ear* or hearing* or deaf* or ‘brain fog*’ or cardiac* or thoracic* or chest* or ischemic* or ischaemic* or heart* or liver* or hepatic* or immuno* or palpitation* or vertigo* or metabol* or vestibular* or endocrine* or encephalit* or physical* or cough* or fibrosis* or myocarditis* or Guillain* or barre* or neuralgi* or amyotroph* or thrombo* or clot* or rash* or hive* or urticari* or lymph* or stroke* or TIA or toe* or foot* or feet* or finger* or chilblain* or numb* or inflammat* or inflame* or arthralgi* or eye* or organ or organs or tingl* or sting* or burn* or bladder* or urogenit* or genitourin* or genital* or reproducti* or urinary* or joint* or tachycard* or atrial* or autoimmun* or dysautonomi* or polyneuro* or mast* or mobility* or walking* or ambulation* or energy*) NEAR/3 (postcovid* or ‘post covid*’ or postcoronavirus* or ‘postcorona* virus*’ or ‘post coronavirus*’ or ‘post corona* virus*’ or postcoronovirus* or ‘postcorono* virus*’ or ‘post coronovirus*’ or ‘post corono* virus*’ or postcoronavirinae* or ‘postcorona* virinae*’ or ‘post coronavirinae*’ or ‘post corona* virinae*’ or postCov or ‘post Cov’ or postsars* or ‘post sars*’ or ‘post severe acute respiratory syndrome*’ or postncov* or ‘post ncov*’ or posthcov* or ‘post hcov*’)):ti,ab | 1071 |
| #30 | ((physiolog* or neuro* or cardio* or gastro* or musculo* or renal* or kidney* or cognitive* or cognition* or rheumato* or dermatol* or skin* or haematol* or blood* or autonomic* or nervous* or ‘nervous system*’ or otolaryngol* or laryngol* or otolog* or cerebro* or brain* or vascular* or respirator* or lung* or pulmonary* or psycholog* or ‘mental health*’ or mental* or psychiatr* or exertion* or debilit* or devitali* or enervat* or drain* or sleep* or weak* or tired* or frail* or sapp* or strength* or confusion* or letharg* or fatigue* or tired* or weariness* or exhaust* or malaise* or pain* or headache* or breathless* or breathing* or myalgia* or delirious* or delirium* or appetite* or muscle* or muscular* or fitness* or memory* or stress* or depress* or anxiety* or emotion* or cough* or fever* or temperatur* or pneumon* or conjunctivit* or throat* or pharyngit* or dyspnea* or dyspnoea* or sick* or nausea* or nauseous* or vomit* or diarrhoea* or diarrhea* or taste* or anosmia* or smell* or olfact* or sweat* or dehydrat* or pyrexi* or nasal* or nose* or mucus* or ear* or hearing* or deaf* or ‘brain fog*’ or cardiac* or thoracic* or chest* or ischemic* or ischaemic* or heart* or liver* or hepatic* or immuno* or palpitation* or vertigo* or metabol* or vestibular* or endocrine* or encephalit* or physical* or cough* or fibrosis* or myocarditis* or Guillain* or barre* or neuralgi* or amyotroph* or thrombo* or clot* or rash* or hive* or urticari* or lymph* or stroke* or TIA or toe* or foot* or feet* or finger* or chilblain* or numb* or inflammat* or inflame* or arthralgi* or eye* or organ or organs or tingl* or sting* or burn* or bladder* or urogenit* or genitourin* or genital* or reproducti* or urinary* or joint* or tachycard* or atrial* or autoimmun* or dysautonomi* or polyneuro* or mast* or mobility* or walking* or ambulation* or energy*) NEAR/1 (sequela* or complication* or consequence* or complexit*) NEAR/6 (covid* or coronavirus* or ‘corona* virus*’ or coronovirus* or ‘corono* virus*’ or coronavirinae* or ‘corona* virinae*’ or Cov or ‘2019-nCoV*’ or 2019nCoV* or ‘19-nCoV*’ or 19nCoV* or nCoV2019* or ‘nCoV-2019*’ or nCoV19* or ‘nCoV-19*’ or ‘HCoV-19*’ or HCoV19* or ‘HCoV-2019*’ or HCoV2019* or ‘2019 novel*’ or Ncov* or ‘n-cov’ or ‘SARS-CoV-2*’ or ‘SARSCoV-2*’ or ‘SARSCoV2*’ or ‘SARS-CoV2*’ or SARSCov19* or ‘SARS-Cov19*’ or ‘SARSCov-19*’ or ‘SARS-Cov-19*’ or SARSCov2019* or ‘SARS-Cov2019*’ or ‘SARSCov-2019*’ or ‘SARS-Cov-2019*’ or SARS2* or ‘SARS-2*’ or SARScoronavirus2* or ‘SARS-coronavirus-2*’ or ‘SARScoronavirus 2*’ or ‘SARS coronavirus2*’ or SARScoronovirus2* or ‘SARS-coronovirus-2*’ or ‘SARScoronovirus 2*’ or ‘SARS coronovirus2*’ or ‘severe acute respiratory syndrome*’)):ti,ab | 2909 |
| #31 | #21 OR #22 OR #23 OR #24 OR #25 OR #28 OR #29 OR #30 | 12909 |
| #32 | #7 OR #31 | 17192 |
| #33 | Letter:it OR editorial:it OR review:it | 4857878 |
| #34 | #32 NOT #33 | 14008 |
| #35 | Nonhuman/de NOT (human/de AND nonhuman/de) | 4992983 |
| #36 | #34 NOT #35 | 13787 |
| #37 | #34 NOT #35 AND [27-10-2020]/sd | 12342 |

## MEDLINE

| Platform | Ovid  Basic Search  Did not include Multimedia or Related Terms |
| --- | --- |
| Search date | 09/05/2022 |
| No. of results | 6202 |

|  |  | No. of results |
| --- | --- | --- |
| #1 | (longcovid* or long covid* or longcoronavirus* or longcorona* virus* or long coronavirus* or long corona* virus* or longcoronovirus* or longcorono* virus* or long coronovirus* or long corono* virus* or longcoronavirinae* or longcorona* virinae* or long coronavirinae* or long corona* virinae* or longCov or long Cov or longsars* or long sars* or long severe acute respiratory syndrome* or longncov* or long ncov* or longhcov* or long hcov*).ti,ab,kw,kf. | 699 |
| #2 | ((long* or endur* or legacy* or slow* or gradual* or protract* or lengthy* or chronic* or persist* or relaps* or remit* or remission* or residual* or delay* or prolong* or extend* or linger* or permanent* or fluctuat* or sequela* or multisystem* or multi system* or nonrecover* or non recover* or subacute* or lasting* or continuous* or continual* or continuing* or postacute* or post acute* or postdischarg* or post discharg* or postinfect* or post infect* or postviral* or post viral* or postvirus* or post virus*) adj1 (covid* or coronavirus* or corona* virus* or coronovirus* or corono* virus* or coronavirinae* or corona* virinae* or Cov or 2019-nCoV* or 2019nCoV* or 19-nCoV* or 19nCoV* or nCoV2019* or nCoV-2019* or nCoV19* or nCoV-19* or HCoV-19* or HCoV19* or HCoV-2019* or HCoV2019* or 2019 novel* or Ncov* or n-cov or SARS-CoV-2* or SARSCoV-2* or SARSCoV2* or SARS-CoV2* or SARSCov19* or SARS-Cov19* or SARSCov-19* or SARS-Cov-19* or SARSCov2019* or SARS-Cov2019* or SARSCov-2019* or SARS-Cov-2019* or SARS2* or SARS-2* or SARScoronavirus2* or SARS-coronavirus-2* or SARScoronavirus 2* or SARS coronavirus2* or SARScoronovirus2* or SARS-coronovirus-2* or SARScoronovirus 2* or SARS coronovirus2* or severe acute respiratory syndrome*)).ti,ab. | 996 |
| #3 | ((long* term* or longterm* or long* haul* or longhaul* or long* tail* or longtail* or longduration* or long duration* or longlast* or long last* or longstanding* or long standing* or medium* term* or mediumterm*) adj3 (covid* or coronavirus* or corona* virus* or coronovirus* or corono* virus* or coronavirinae* or corona* virinae* or Cov or 2019-nCoV* or 2019nCoV* or 19-nCoV* or 19nCoV* or nCoV2019* or nCoV-2019* or nCoV19* or nCoV-19* or HCoV-19* or HCoV19* or HCoV-2019* or HCoV2019* or 2019 novel* or Ncov* or n-cov or SARS-CoV-2* or SARSCoV-2* or SARSCoV2* or SARS-CoV2* or SARSCov19* or SARS-Cov19* or SARSCov-19* or SARS-Cov-19* or SARSCov2019* or SARS-Cov2019* or SARSCov-2019* or SARS-Cov-2019* or SARS2* or SARS-2* or SARScoronavirus2* or SARS-coronavirus-2* or SARScoronavirus 2* or SARS coronavirus2* or SARScoronovirus2* or SARS-coronovirus-2* or SARScoronovirus 2* or SARS coronovirus2* or severe acute respiratory syndrome*)).ti,ab. | 715 |
| #4 | ((postcovid* or post covid* or postcoronavirus* or postcorona* virus* or post coronavirus* or post corona* virus* or postcoronovirus* or postcorono* virus* or post coronovirus* or post corono* virus* or postcoronavirinae* or postcorona* virinae* or post coronavirinae* or post corona* virinae* or postCov or post Cov or postsars* or post sars* or post severe acute respiratory syndrome* or postncov* or post ncov* or posthcov* or post hcov*) adj3 (syndrome* or disorder* or illness* or sickness* or disease* or condition* or symptom* or sign* or prognos* or followup* or follow up* or feature* or comorbid* or co morbid* or multimorbid* or multi morbid* or survivor* or survival* or risk* or care* or convalescen* or recuperat* or aftercare* or ambulatory* or outpatient* or out patient*)).ti,ab. | 544 |
| #5 | ((ongoing* or long* or endur* or legacy* or slow* or gradual* or protract* or lengthy* or chronic* or persist* or relaps* or remit* or remission* or residual* or delay* or prolong* or extend* or linger* or permanent* or fluctuat* or multisystem* or multi system* or nonrecover* or non recover* or subacute* or lasting* or continuous* or continual* or continuing* or postacute* or post acute* or postdischarg* or post discharg* or postinfect* or post infect* or postviral* or post viral* or postvirus* or post virus* or medium* term* or mediumterm*) adj4 (sequela* or illness* or symptom* or sign* or prognos* or rehab* or convalescen* or recuperat* or followup* or follow up* or feature*) adj10 (covid* or coronavirus* or corona* virus* or coronovirus* or corono* virus* or coronavirinae* or corona* virinae* or Cov or 2019-nCoV* or 2019nCoV* or 19-nCoV* or 19nCoV* or nCoV2019* or nCoV-2019* or nCoV19* or nCoV-19* or HCoV-19* or HCoV19* or HCoV-2019* or HCoV2019* or 2019 novel* or Ncov* or n-cov or SARS-CoV-2* or SARSCoV-2* or SARSCoV2* or SARS-CoV2* or SARSCov19* or SARS-Cov19* or SARSCov-19* or SARS-Cov-19* or SARSCov2019* or SARS-Cov2019* or SARSCov-2019* or SARS-Cov-2019* or SARS2* or SARS-2* or SARScoronavirus2* or SARS-coronavirus-2* or SARScoronavirus 2* or SARS coronavirus2* or SARScoronovirus2* or SARS-coronovirus-2* or SARScoronovirus 2* or SARS coronovirus2* or severe acute respiratory syndrome*)).ti,ab. | 1652 |
| #6 | ((ongoing* or long* or endur* or legacy* or slow* or gradual* or protract* or lengthy* or chronic* or persist* or relaps* or remit* or remission* or residual* or delay* or prolong* or extend* or linger* or permanent* or fluctuat* or multisystem* or multi system* or subacute* or lasting* or continuous* or continual* or continuing* or post* or after* or follow* or medium* term* or mediumterm*) adj1 recover* adj10 (covid* or coronavirus* or corona* virus* or coronovirus* or corono* virus* or coronavirinae* or corona* virinae* or Cov or 2019-nCoV* or 2019nCoV* or 19-nCoV* or 19nCoV* or nCoV2019* or nCoV-2019* or nCoV19* or nCoV-19* or HCoV-19* or HCoV19* or HCoV-2019* or HCoV2019* or 2019 novel* or Ncov* or n-cov or SARS-CoV-2* or SARSCoV-2* or SARSCoV2* or SARS-CoV2* or SARSCov19* or SARS-Cov19* or SARSCov-19* or SARS-Cov-19* or SARSCov2019* or SARS-Cov2019* or SARSCov-2019* or SARS-Cov-2019* or SARS2* or SARS-2* or SARScoronavirus2* or SARS-coronavirus-2* or SARScoronavirus 2* or SARS coronavirus2* or SARScoronovirus2* or SARS-coronovirus-2* or SARScoronovirus 2* or SARS coronovirus2* or severe acute respiratory syndrome*)).ti,ab. | 472 |
| #7 | or/1-6 | 3532 |
| #8 | exp coronavirus/ | 135692 |
| #9 | exp Coronavirus Infections/ | 167199 |
| #10 | (covid* or coronavirus* or corona* virus* or coronovirus* or corono* virus* or coronavirinae* or corona* virinae* or Cov or 2019-nCoV* or 2019nCoV* or 19-nCoV* or 19nCoV* or nCoV2019* or nCoV-2019* or nCoV19* or nCoV-19* or HCoV-19* or HCoV19* or HCoV-2019* or HCoV2019* or 2019 novel* or Ncov* or n-cov or SARS-CoV-2* or SARSCoV-2* or SARSCoV2* or SARS-CoV2* or SARSCov19* or SARS-Cov19* or SARSCov-19* or SARS-Cov-19* or SARSCov2019* or SARS-Cov2019* or SARSCov-2019* or SARS-Cov-2019* or SARS2* or SARS-2* or SARScoronavirus2* or SARS-coronavirus-2* or SARScoronavirus 2* or SARS coronavirus2* or SARScoronovirus2* or SARS-coronovirus-2* or SARScoronovirus 2* or SARS coronovirus2* or severe acute respiratory syndrome*).ti. | 147660 |
| #11 | or/8-10 | 175984 |
| #12 | Recovery of Function/ | 58346 |
| #13 | Aftercare/ | 11624 |
| #14 | rehabilitation/ | 18651 |
| #15 | Activities of Daily Living/ | 70161 |
| #16 | convalescence/ | 3910 |
| #17 | Long Term Adverse Effects/ | 801 |
| #18 | Physical Functional Performance/ | 2322 |
| #19 | or/12-18 | 160630 |
| #20 | 11 and 19 | 1111 |
| #21 | (long* haul* or longhaul* or long* tail* or longtail* or longduration* or long duration* or longlast* or long last* or longstanding* or long standing*).ti,ab. and (8 or 9) | 0 |
| #22 | ((recover* or nonrecover*) adj3 function* adj10 (covid* or coronavirus* or corona* virus* or coronovirus* or corono* virus* or coronavirinae* or corona* virinae* or Cov or 2019-nCoV* or 2019nCoV* or 19-nCoV* or 19nCoV* or nCoV2019* or nCoV-2019* or nCoV19* or nCoV-19* or HCoV-19* or HCoV19* or HCoV-2019* or HCoV2019* or 2019 novel* or Ncov* or n-cov or SARS-CoV-2* or SARSCoV-2* or SARSCoV2* or SARS-CoV2* or SARSCov19* or SARS-Cov19* or SARSCov-19* or SARS-Cov-19* or SARSCov2019* or SARS-Cov2019* or SARSCov-2019* or SARS-Cov-2019* or SARS2* or SARS-2* or SARScoronavirus2* or SARS-coronavirus-2* or SARScoronavirus 2* or SARS coronavirus2* or SARScoronovirus2* or SARS-coronovirus-2* or SARScoronovirus 2* or SARS coronovirus2* or severe acute respiratory syndrome*)).ti,ab. | 59 |
| #23 | ((postacute* or post acute* or postdischarg* or post discharg* or postinfect* or post infect* or postviral* or post viral* or postvirus* or post virus* or subacute*) adj3 (care* or convalescen* or recuperat* or aftercare* or ambulatory* or outpatient* or out patient* or survivor* or survival*) adj10 (covid* or coronavirus* or corona* virus* or coronovirus* or corono* virus* or coronavirinae* or corona* virinae* or Cov or 2019-nCoV* or 2019nCoV* or 19-nCoV* or 19nCoV* or nCoV2019* or nCoV-2019* or nCoV19* or nCoV-19* or HCoV-19* or HCoV19* or HCoV-2019* or HCoV2019* or 2019 novel* or Ncov* or n-cov or SARS-CoV-2* or SARSCoV-2* or SARSCoV2* or SARS-CoV2* or SARSCov19* or SARS-Cov19* or SARSCov-19* or SARS-Cov-19* or SARSCov2019* or SARS-Cov2019* or SARSCov-2019* or SARS-Cov-2019* or SARS2* or SARS-2* or SARScoronavirus2* or SARS-coronavirus-2* or SARScoronavirus 2* or SARS coronavirus2* or SARScoronovirus2* or SARS-coronovirus-2* or SARScoronovirus 2* or SARS coronovirus2* or severe acute respiratory syndrome*)).ti,ab. | 59 |
| #24 | ((convalescen* or recuperat* or after* or followup* or follow up* or rehab*) adj1 (therap* or care*) adj10 (covid* or coronavirus* or corona* virus* or coronovirus* or corono* virus* or coronavirinae* or corona* virinae* or Cov or 2019-nCoV* or 2019nCoV* or 19-nCoV* or 19nCoV* or nCoV2019* or nCoV-2019* or nCoV19* or nCoV-19* or HCoV-19* or HCoV19* or HCoV-2019* or HCoV2019* or 2019 novel* or Ncov* or n-cov or SARS-CoV-2* or SARSCoV-2* or SARSCoV2* or SARS-CoV2* or SARSCov19* or SARS-Cov19* or SARSCov-19* or SARS-Cov-19* or SARSCov2019* or SARS-Cov2019* or SARSCov-2019* or SARS-Cov-2019* or SARS2* or SARS-2* or SARScoronavirus2* or SARS-coronavirus-2* or SARScoronavirus 2* or SARS coronavirus2* or SARScoronovirus2* or SARS-coronovirus-2* or SARScoronovirus 2* or SARS coronovirus2* or severe acute respiratory syndrome*)).ti,ab. | 155 |
| #25 | ((ongoing* or endur* or long* or legacy* or slow* or gradual* or protract* or lengthy* or chronic* or persist* or relaps* or remit* or remission* or residual* or delay* or prolong* or extend* or linger* or permanent* or fluctuat* or multisystem* or multi system* or nonrecover* or non recover* or subacute* or lasting* or continuous* or continual* or continuing* or postacute* or post acute* or postdischarg* or post discharg* or postinfect* or post infect* or postviral* or post viral* or postvirus* or post virus* or medium* term* or mediumterm* or adverse* or dangerous* or harmful* or indirect* or injurious* or secondary* or side effect* or undesirable* or sequela* or complication* or consequence* or effect* or event* or impact* or outcome* or reaction* or complexit* or aftercare* or impair* or problem* or issue* or rehab* or function* or perform*) adj10 ((daily* or everyday* or day* or normal* or usual*) adj1 (activit* or living* or life* or lives* or job* or work* or employ* or occupation* or hobby* or hobbies* or leisure*)) adj10 (covid* or coronavirus* or corona* virus* or coronovirus* or corono* virus* or coronavirinae* or corona* virinae* or Cov or 2019-nCoV* or 2019nCoV* or 19-nCoV* or 19nCoV* or nCoV2019* or nCoV-2019* or nCoV19* or nCoV-19* or HCoV-19* or HCoV19* or HCoV-2019* or HCoV2019* or 2019 novel* or Ncov* or n-cov or SARS-CoV-2* or SARSCoV-2* or SARSCoV2* or SARS-CoV2* or SARSCov19* or SARS-Cov19* or SARSCov-19* or SARS-Cov-19* or SARSCov2019* or SARS-Cov2019* or SARSCov-2019* or SARS-Cov-2019* or SARS2* or SARS-2* or SARScoronavirus2* or SARS-coronavirus-2* or SARScoronavirus 2* or SARS coronavirus2* or SARScoronovirus2* or SARS-coronovirus-2* or SARScoronovirus 2* or SARS coronovirus2* or severe acute respiratory syndrome*)).ti,ab. | 271 |
| #26 | ((ongoing* or endur* or long* or legacy* or slow* or gradual* or protract* or lengthy* or chronic* or persist* or relaps* or remit* or remission* or residual* or delay* or prolong* or extend* or linger* or permanent* or fluctuat* or multisystem* or multi system* or nonrecover* or non recover* or subacute* or lasting* or continuous* or continual* or continuing* or postacute* or post acute* or postdischarg* or post discharg* or postinfect* or post infect* or postviral* or post viral* or postvirus* or post virus* or medium* term* or mediumterm*) adj3 ((health* or adverse* or dangerous* or harmful* or indirect* or injurious* or secondary* or side* or undesirable* or negative* or damaging* or detriment* or abnormal*) adj1 (sequela* or complication* or consequence* or effect* or event* or impact* or outcome* or reaction* or complexit* or aftercare* or impair* or problem* or issue* or symptom* or disorder*)) adj10 (covid* or coronavirus* or corona* virus* or coronovirus* or corono* virus* or coronavirinae* or corona* virinae* or Cov or 2019-nCoV* or 2019nCoV* or 19-nCoV* or 19nCoV* or nCoV2019* or nCoV-2019* or nCoV19* or nCoV-19* or HCoV-19* or HCoV19* or HCoV-2019* or HCoV2019* or 2019 novel* or Ncov* or n-cov or SARS-CoV-2* or SARSCoV-2* or SARSCoV2* or SARS-CoV2* or SARSCov19* or SARS-Cov19* or SARSCov-19* or SARS-Cov-19* or SARSCov2019* or SARS-Cov2019* or SARSCov-2019* or SARS-Cov-2019* or SARS2* or SARS-2* or SARScoronavirus2* or SARS-coronavirus-2* or SARScoronavirus 2* or SARS coronavirus2* or SARScoronovirus2* or SARS-coronovirus-2* or SARScoronovirus 2* or SARS coronovirus2* or severe acute respiratory syndrome*)).ti,ab. | 170 |
| #27 | ((ongoing* or endur* or long* or legacy* or slow* or gradual* or protract* or lengthy* or chronic* or persist* or relaps* or remit* or remission* or residual* or delay* or prolong* or extend* or linger* or permanent* or fluctuat* or multisystem* or multi system* or nonrecover* or non recover* or subacute* or lasting* or continuous* or continual* or continuing* or postacute* or post acute* or postdischarg* or post discharg* or postinfect* or post infect* or postviral* or post viral* or postvirus* or post virus* or medium* term* or mediumterm*) adj3 ((physiolog* or neuro* or cardio* or gastro* or musculo* or renal* or kidney* or cognitive* or cognition* or rheumato* or dermatol* or skin* or haematol* or blood* or autonomic* or nervous* or nervous system* or otolaryngol* or laryngol* or otolog* or cerebro* or brain* or vascular* or respirator* or lung* or pulmonary* or psycholog* or mental health* or mental* or psychiatr* or exertion* or debilit* or devitali* or enervat* or drain* or sleep* or weak* or tired* or frail* or sapp* or strength* or confusion* or letharg* or fatigue* or tired* or weariness* or exhaust* or malaise* or pain* or headache* or breathless* or breathing* or myalgia* or delirious* or delirium* or appetite* or muscle* or muscular* or fitness* or memory* or stress* or depress* or anxiety* or emotion* or cough* or fever* or temperatur* or pneumon* or conjunctivit* or throat* or pharyngit* or dyspnea* or dyspnoea* or sick* or nausea* or nauseous* or vomit* or diarrhoea* or diarrhea* or taste* or anosmia* or smell* or olfact* or sweat* or dehydrat* or pyrexi* or nasal* or nose* or mucus* or ear* or hearing* or deaf* or brain fog* or cardiac* or thoracic* or chest* or ischemic* or ischaemic* or heart* or liver* or hepatic* or immuno* or palpitation* or vertigo* or metabol* or vestibular* or endocrine* or encephalit* or physical* or cough* or fibrosis* or myocarditis* or Guillain* or barre* or neuralgi* or amyotroph* or thrombo* or clot* or rash* or hive* or urticari* or lymph* or stroke* or TIA or toe* or foot* or feet* or finger* or chilblain* or numb* or inflammat* or inflame* or arthralgi* or eye* or organ or organs or tingl* or sting* or burn* or bladder* or urogenit* or genitourin* or genital* or reproducti* or urinary* or joint* or tachycard* or atrial* or autoimmun* or dysautonomi* or polyneuro* or mast* or mobility* or walking* or ambulation* or energy*) adj1 (sequela* or complication* or consequence* or effect* or event* or impact* or outcome* or reaction* or complexit* or aftercare* or impair* or problem* or issue* or symptom* or disorder* or abnormal*)) adj10 (covid* or coronavirus* or corona* virus* or coronovirus* or corono* virus* or coronavirinae* or corona* virinae* or Cov or 2019-nCoV* or 2019nCoV* or 19-nCoV* or 19nCoV* or nCoV2019* or nCoV-2019* or nCoV19* or nCoV-19* or HCoV-19* or HCoV19* or HCoV-2019* or HCoV2019* or 2019 novel* or Ncov* or n-cov or SARS-CoV-2* or SARSCoV-2* or SARSCoV2* or SARS-CoV2* or SARSCov19* or SARS-Cov19* or SARSCov-19* or SARS-Cov-19* or SARSCov2019* or SARS-Cov2019* or SARSCov-2019* or SARS-Cov-2019* or SARS2* or SARS-2* or SARScoronavirus2* or SARS-coronavirus-2* or SARScoronavirus 2* or SARS coronavirus2* or SARScoronovirus2* or SARS-coronovirus-2* or SARScoronovirus 2* or SARS coronovirus2* or severe acute respiratory syndrome*)).ti,ab. | 375 |
| #28 | ((physiolog* or neuro* or cardio* or gastro* or musculo* or renal* or kidney* or cognitive* or cognition* or rheumato* or dermatol* or skin* or haematol* or blood* or autonomic* or nervous* or nervous system* or otolaryngol* or laryngol* or otolog* or cerebro* or brain* or vascular* or respirator* or lung* or pulmonary* or psycholog* or mental health* or mental* or psychiatr* or exertion* or debilit* or devitali* or enervat* or drain* or sleep* or weak* or tired* or frail* or sapp* or strength* or confusion* or letharg* or fatigue* or tired* or weariness* or exhaust* or malaise* or pain* or headache* or breathless* or breathing* or myalgia* or delirious* or delirium* or appetite* or muscle* or muscular* or fitness* or memory* or stress* or depress* or anxiety* or emotion* or cough* or fever* or temperatur* or pneumon* or conjunctivit* or throat* or pharyngit* or dyspnea* or dyspnoea* or sick* or nausea* or nauseous* or vomit* or diarrhoea* or diarrhea* or taste* or anosmia* or smell* or olfact* or sweat* or dehydrat* or pyrexi* or nasal* or nose* or mucus* or ear* or hearing* or deaf* or brain fog* or cardiac* or thoracic* or chest* or ischemic* or ischaemic* or heart* or liver* or hepatic* or immuno* or palpitation* or vertigo* or metabol* or vestibular* or endocrine* or encephalit* or physical* or cough* or fibrosis* or myocarditis* or Guillain* or barre* or neuralgi* or amyotroph* or thrombo* or clot* or rash* or hive* or urticari* or lymph* or stroke* or TIA or toe* or foot* or feet* or finger* or chilblain* or numb* or inflammat* or inflame* or arthralgi* or eye* or organ or organs or tingl* or sting* or burn* or bladder* or urogenit* or genitourin* or genital* or reproducti* or urinary* or joint* or tachycard* or atrial* or autoimmun* or dysautonomi* or polyneuro* or mast* or mobility* or walking* or ambulation* or energy*) adj3 (postcovid* or post covid* or postcoronavirus* or postcorona* virus* or post coronavirus* or post corona* virus* or postcoronovirus* or postcorono* virus* or post coronovirus* or post corono* virus* or postcoronavirinae* or postcorona* virinae* or post coronavirinae* or post corona* virinae* or postCov or post Cov or postsars* or post sars* or post severe acute respiratory syndrome* or postncov* or post ncov* or posthcov* or post hcov*)).ti,ab. | 407 |
| #29 | ((physiolog* or neuro* or cardio* or gastro* or musculo* or renal* or kidney* or cognitive* or cognition* or rheumato* or dermatol* or skin* or haematol* or blood* or autonomic* or nervous* or nervous system* or otolaryngol* or laryngol* or otolog* or cerebro* or brain* or vascular* or respirator* or lung* or pulmonary* or psycholog* or mental health* or mental* or psychiatr* or exertion* or debilit* or devitali* or enervat* or drain* or sleep* or weak* or tired* or frail* or sapp* or strength* or confusion* or letharg* or fatigue* or tired* or weariness* or exhaust* or malaise* or pain* or headache* or breathless* or breathing* or myalgia* or delirious* or delirium* or appetite* or muscle* or muscular* or fitness* or memory* or stress* or depress* or anxiety* or emotion* or cough* or fever* or temperatur* or pneumon* or conjunctivit* or throat* or pharyngit* or dyspnea* or dyspnoea* or sick* or nausea* or nauseous* or vomit* or diarrhoea* or diarrhea* or taste* or anosmia* or smell* or olfact* or sweat* or dehydrat* or pyrexi* or nasal* or nose* or mucus* or ear* or hearing* or deaf* or brain fog* or cardiac* or thoracic* or chest* or ischemic* or ischaemic* or heart* or liver* or hepatic* or immuno* or palpitation* or vertigo* or metabol* or vestibular* or endocrine* or encephalit* or physical* or cough* or fibrosis* or myocarditis* or Guillain* or barre* or neuralgi* or amyotroph* or thrombo* or clot* or rash* or hive* or urticari* or lymph* or stroke* or TIA or toe* or foot* or feet* or finger* or chilblain* or numb* or inflammat* or inflame* or arthralgi* or eye* or organ or organs or tingl* or sting* or burn* or bladder* or urogenit* or genitourin* or genital* or reproducti* or urinary* or joint* or tachycard* or atrial* or autoimmun* or dysautonomi* or polyneuro* or mast* or mobility* or walking* or ambulation* or energy*) adj1 (sequela* or complication* or consequence* or complexit*) adj10 (covid* or coronavirus* or corona* virus* or coronovirus* or corono* virus* or coronavirinae* or corona* virinae* or Cov or 2019-nCoV* or 2019nCoV* or 19-nCoV* or 19nCoV* or nCoV2019* or nCoV-2019* or nCoV19* or nCoV-19* or HCoV-19* or HCoV19* or HCoV-2019* or HCoV2019* or 2019 novel* or Ncov* or n-cov or SARS-CoV-2* or SARSCoV-2* or SARSCoV2* or SARS-CoV2* or SARSCov19* or SARS-Cov19* or SARSCov-19* or SARS-Cov-19* or SARSCov2019* or SARS-Cov2019* or SARSCov-2019* or SARS-Cov-2019* or SARS2* or SARS-2* or SARScoronavirus2* or SARS-coronavirus-2* or SARScoronavirus 2* or SARS coronavirus2* or SARScoronovirus2* or SARS-coronovirus-2* or SARScoronovirus 2* or SARS coronovirus2* or severe acute respiratory syndrome*)).ti,ab. | 1644 |
| #30 | (((((multi or multiple* or overlap* or cluster* or numerous* or varied* or variety*) adj1 (symptom* or system* or disease* or disorder* or illness* or condition* or syndrome*)) or (multisymptom* or multisystem* or multidisease* or multidisorder* or multiillness* or multicondition* or multisyndrom*)) adj10 (covid* or coronavirus* or corona* virus* or coronovirus* or corono* virus* or coronavirinae* or corona* virinae* or Cov or 2019-nCoV* or 2019nCoV* or 19-nCoV* or 19nCoV* or nCoV2019* or nCoV-2019* or nCoV19* or nCoV-19* or HCoV-19* or HCoV19* or HCoV-2019* or HCoV2019* or 2019 novel* or Ncov* or n-cov or SARS-CoV-2* or SARSCoV-2* or SARSCoV2* or SARS-CoV2* or SARSCov19* or SARS-Cov19* or SARSCov-19* or SARS-Cov-19* or SARSCov2019* or SARS-Cov2019* or SARSCov-2019* or SARS-Cov-2019* or SARS2* or SARS-2* or SARScoronavirus2* or SARS-coronavirus-2* or SARScoronavirus 2* or SARS coronavirus2* or SARScoronovirus2* or SARS-coronovirus-2* or SARScoronovirus 2* or SARS coronovirus2* or severe acute respiratory syndrome*)) not (Multisystem* inflammatory* syndrome* or inflammatory* multisystem* syndrome*)).ti,ab. | 338 |
| #31 | or/20-30 | 4211 |
| #32 | 7 or 31 | 6962 |
| #33 | limit 32 to (letter or historical article or comment or editorial or news) | 759 |
| #34 | 32 not 33 | 6203 |
| #35 | Animals/ not (Animals/ and Humans/) | 11274 |
| #36 | 34 not 35 | 6202 |

## Cochrane Library

| Platform | Wiley |
| --- | --- |
| Search date | 03/06/2022 |
| No. of results | 542 |

|  |  | No. of results |
| --- | --- | --- |
| #1 | (longcovid* or (long NEXT covid*) or longcoronavirus* or (longcorona* NEXT virus*) or (long NEXT coronavirus*) or (long NEXT corona* NEXT virus*) or longcoronovirus* or (longcorono* NEXT virus*) or (long NEXT coronovirus*) or (long NEXT corono* NEXT virus*) or longcoronavirinae* or (longcorona* NEXT virinae*) or (long NEXT coronavirinae*) or (long NEXT corona* NEXT virinae*) or longCov or (long NEXT Cov) or longsars* or (long NEXT sars*) or "long severe acute respiratory syndrome" or "long severe acute respiratory syndromes" or longncov* or (long NEXT ncov*) or longhcov* or (long NEXT hcov*)):ti,ab | 76 |
| #2 | ((long* or endur* or legacy* or slow* or gradual* or protract* or lengthy* or chronic* or persist* or relaps* or remit* or remission* or residual* or delay* or prolong* or extend* or linger* or permanent* or fluctuat* or sequela* or multisystem* or "multi system*" or nonrecover* or "non recovery" or "non recovering" or subacute* or "sub acute*" or lasting* or continuous* or continual* or continuing* or postacute* or "post acute*" or postdischarg* or "post discharge" or "post discharging" or postinfect* or "post infection" or "post infective*" or postviral* or "post viral*" or postvirus* or "post virus*") near/1 (covid* or coronavirus* or "corona* virus*" or coronovirus* or "corono* virus*" or coronavirinae* or "corona* virinae*" or Cov or "2019-nCoV*" or 2019nCoV* or "19-nCoV*" or 19nCoV* or nCoV2019* or "nCoV-2019*" or nCoV19* or "nCoV-19*" or "HCoV-19*" or HCoV19* or "HCoV-2019*" or HCoV2019* or "2019 novel*" or Ncov* or "n-cov" or "SARS-CoV-2*" or "SARSCoV-2*" or "SARSCoV2*" or "SARS-CoV2*" or SARSCov19* or "SARS-Cov19*" or "SARSCov-19*" or "SARS-Cov-19*" or SARSCov2019* or "SARS-Cov2019*" or "SARSCov-2019*" or "SARS-Cov-2019*" or SARS2* or "SARS-2*" or SARScoronavirus2* or "SARS-coronavirus-2*" or "SARScoronavirus 2*" or "SARS coronavirus2*" or SARScoronovirus2* or "SARS-coronovirus-2*" or "SARScoronovirus 2*" or "SARS coronovirus2*" or "severe acute respiratory syndrome*")):ti,ab | 127 |
| #3 | (("long* term*" or longterm* or "long* haul*" or longhaul* or "long* tail*" or longtail* or longduration* or "long duration*" or longlast* or "long last*" or longstanding* or "long standing*" or "medium* term*" or mediumterm*) near/3 (covid* or coronavirus* or "corona* virus*" or coronovirus* or "corono* virus*" or coronavirinae* or "corona* virinae*" or Cov or "2019-nCoV*" or 2019nCoV* or "19-nCoV*" or 19nCoV* or nCoV2019* or "nCoV-2019*" or nCoV19* or "nCoV-19*" or "HCoV-19*" or HCoV19* or "HCoV-2019*" or HCoV2019* or "2019 novel*" or Ncov* or "n-cov" or "SARS-CoV-2*" or "SARSCoV-2*" or "SARSCoV2*" or "SARS-CoV2*" or SARSCov19* or "SARS-Cov19*" or "SARSCov-19*" or "SARS-Cov-19*" or SARSCov2019* or "SARS-Cov2019*" or "SARSCov-2019*" or "SARS-Cov-2019*" or SARS2* or "SARS-2*" or SARScoronavirus2* or "SARS-coronavirus-2*" or "SARScoronavirus 2*" or "SARS coronavirus2*" or SARScoronovirus2* or "SARS-coronovirus-2*" or "SARScoronovirus 2*" or "SARS coronovirus2*" or "severe acute respiratory syndrome*")):ti,ab | 52 |
| #4 | ((postcovid* or "post covid*" or postcoronavirus* or "postcorona* virus*" or "post coronavirus*" or "post corona* virus*" or postcoronovirus* or "postcorono* virus*" or "post coronovirus*" or "post corono* virus*" or postcoronavirinae* or "postcorona* virinae*" or "post coronavirinae*" or "post corona* virinae*" or postCov or "post Cov" or postsars* or "post sars*" or "post severe acute respiratory syndrome*" or postncov* or "post ncov*" or posthcov* or "post hcov*") near/3 (syndrome* or disorder* or illness* or sickness* or disease* or condition* or symptom* or sign* or prognos* or followup* or "follow up*" or feature* or comorbid* or "co morbid*" or multimorbid* or "multi morbid*" or survivor* or survival* or risk* or care* or convalescen* or recuperat* or aftercare* or ambulatory* or outpatient* or "out patient*" or "out patients*")):ti,ab | 77 |
| #5 | ((ongoing* or long* or endur* or legacy* or slow* or gradual* or protract* or lengthy* or chronic* or persist* or relaps* or remit* or remission* or residual* or delay* or prolong* or extend* or linger* or permanent* or fluctuat* or multisystem* or "multi system*" or nonrecover* or "non recovery" or "non recovering*" or subacute* or "sub acute*" or lasting* or continuous* or continual* or continuing* or postacute* or "post acute*" or postdischarg* or "post discharge" or "post discharging" or postinfect* or "post infection" or "post infective*" or postviral* or "post viral*" or postvirus* or "post virus*" or "medium* term*" or mediumterm*) near/4 (sequela* or illness* or symptom* or sign* or prognos* or rehab* or convalescen* or recuperat* or followup* or "follow up*" or feature*) near/10 (covid* or coronavirus* or "corona* virus*" or coronovirus* or "corono* virus*" or coronavirinae* or "corona* virinae*" or Cov or "2019-nCoV*" or 2019nCoV* or "19-nCoV*" or 19nCoV* or nCoV2019* or "nCoV-2019*" or nCoV19* or "nCoV-19*" or "HCoV-19*" or HCoV19* or "HCoV-2019*" or HCoV2019* or "2019 novel*" or Ncov* or "n-cov" or "SARS-CoV-2*" or "SARSCoV-2*" or "SARSCoV2*" or "SARS-CoV2*" or SARSCov19* or "SARS-Cov19*" or "SARSCov-19*" or "SARS-Cov-19*" or SARSCov2019* or "SARS-Cov2019*" or "SARSCov-2019*" or "SARS-Cov-2019*" or SARS2* or "SARS-2*" or SARScoronavirus2* or "SARS-coronavirus-2*" or "SARScoronavirus 2*" or "SARS coronavirus2*" or SARScoronovirus2* or "SARS-coronovirus-2*" or "SARScoronovirus 2*" or "SARS coronovirus2*" or "severe acute respiratory syndrome*")):ti,ab | 181 |
| #6 | ((ongoing* or long* or endur* or legacy* or slow* or gradual* or protract* or lengthy* or chronic* or persist* or relaps* or remit* or remission* or residual* or delay* or prolong* or extend* or linger* or permanent* or fluctuat* or multisystem* or "multi system*" or subacute* or "sub acute*" or lasting* or continuous* or continual* or continuing* or post* or after* or follow* or "medium* term*" or mediumterm*) near/1 recover* near/10 (covid* or coronavirus* or "corona* virus*" or coronovirus* or "corono* virus*" or coronavirinae* or "corona* virinae*" or Cov or "2019-nCoV*" or 2019nCoV* or "19-nCoV*" or 19nCoV* or nCoV2019* or "nCoV-2019*" or nCoV19* or "nCoV-19*" or "HCoV-19*" or HCoV19* or "HCoV-2019*" or HCoV2019* or "2019 novel*" or Ncov* or "n-cov" or "SARS-CoV-2*" or "SARSCoV-2*" or "SARSCoV2*" or "SARS-CoV2*" or SARSCov19* or "SARS-Cov19*" or "SARSCov-19*" or "SARS-Cov-19*" or SARSCov2019* or "SARS-Cov2019*" or "SARSCov-2019*" or "SARS- Cov-2019*" or SARS2* or "SARS-2*" or SARScoronavirus2* or "SARS-coronavirus-2*" or "SARScoronavirus 2*" or "SARS coronavirus2*" or SARScoronovirus2* or "SARS-coronovirus-2*" or "SARScoronovirus 2*" or "SARS coronovirus2*" or "severe acute respiratory syndrome*")):ti,ab | 51 |
| #7 | {or #1-#6} | 348 |
| #8 | [mh "coronavirus"] | 998 |
| #9 | [mh "coronavirus infections"] | 2406 |
| #10 | (covid* or coronavirus* or "corona* virus*" or coronovirus* or "corono* virus*" or coronavirinae* or "corona* virinae*" or Cov or "2019-nCoV*" or 2019nCoV* or "19-nCoV*" or 19nCoV* or nCoV2019* or "nCoV-2019*" or nCoV19* or "nCoV-19*" or "HCoV-19*" or HCoV19* or "HCoV-2019*" or HCoV2019* or "2019 novel*" or Ncov* or "n-cov" or "SARS-CoV-2*" or "SARSCoV-2*" or "SARSCoV2*" or "SARS-CoV2*" or SARSCov19* or "SARS-Cov19*" or "SARSCov-19*" or "SARS-Cov-19*" or SARSCov2019* or "SARS-Cov2019*" or "SARSCov-2019*" or "SARS-Cov-2019*" or SARS2* or "SARS-2*" or SARScoronavirus2* or "SARS-coronavirus-2*" or "SARScoronavirus 2*" or "SARS coronavirus2*" or SARScoronovirus2* or "SARS-coronovirus-2*" or "SARScoronovirus 2*" or "SARS coronovirus2*" or "severe acute respiratory syndrome*"):ti | 8751 |
| #11 | {or #8-#10} | 9150 |
| #12 | [mh ^"Recovery of Function"] | 5700 |
| #13 | [mh ^"Aftercare"] | 774 |
| #14 | [mh ^"Rehabilitation"] | 322 |
| #15 | [mh ^"Activities of Daily Living"] | 5300 |
| #16 | [mh ^"Convalescence"] | 158 |
| #17 | [mh ^"Long Term Adverse Effects"] | 51 |
| #18 | [mh ^"Physical Functional Performance"] | 310 |
| #19 | {or #12-#18} | 12030 |
| #20 | #11 and #19 | 31 |
| #21 | ("long* haul*" or longhaul* or "long* tail*" or longtail* or longduration* or "long duration*" or longlast* or "long last*" or longstanding* or "long standing*"):ti,ab | 6226 |
| #22 | #8 or #9 | 2411 |
| #23 | #21 and #22 | 16 |
| #24 | ((recover* or nonrecover*) near/3 function* near/10 (covid* or coronavirus* or "corona* virus*" or coronovirus* or "corono* virus*" or coronavirinae* or "corona* virinae*" or Cov or "2019-nCoV*" or 2019nCoV* or "19-nCoV*" or 19nCoV* or nCoV2019* or "nCoV-2019*" or nCoV19* or "nCoV-19*" or "HCoV-19*" or HCoV19* or "HCoV-2019*" or HCoV2019* or "2019 novel*" or Ncov* or "n-cov" or "SARS-CoV-2*" or "SARSCoV-2*" or "SARSCoV2*" or "SARS-CoV2*" or SARSCov19* or "SARS-Cov19*" or "SARSCov-19*" or "SARS-Cov-19*" or SARSCov2019* or "SARS-Cov2019*" or "SARSCov-2019*" or "SARS-Cov-2019*" or SARS2* or "SARS-2*" or SARScoronavirus2* or "SARS-coronavirus-2*" or "SARScoronavirus 2*" or "SARS coronavirus2*" or SARScoronovirus2* or "SARS-coronovirus-2*" or "SARScoronovirus 2*" or "SARS coronovirus2*" or "severe acute respiratory syndrome*")):ti,ab | 8 |
| #25 | ((postacute* or "post acute*" or postdischarg* or "post discharge" or "post discharging" or postinfect* or "post infection*" or "post infective*" or postviral* or "post viral*" or postvirus* or "post virus*" or subacute* or "sub acute*") near/3 (care* or convalescen* or recuperat* or aftercare* or ambulatory* or outpatient* or "out patient" or "out patients*" or survivor* or survival*) near/10 (covid* or coronavirus* or "corona* virus*" or coronovirus* or "corono* virus*" or coronavirinae* or "corona* virinae*" or Cov or "2019-nCoV*" or 2019nCoV* or "19-nCoV*" or 19nCoV* or nCoV2019* or "nCoV-2019*" or nCoV19* or "nCoV-19*" or "HCoV-19*" or HCoV19* or "HCoV-2019*" or HCoV2019* or "2019 novel*" or Ncov* or "n-cov" or "SARS-CoV-2*" or "SARSCoV-2*" or "SARSCoV2*" or "SARS-CoV2*" or SARSCov19* or "SARS-Cov19*" or "SARSCov-19*" or "SARS-Cov-19*" or SARSCov2019* or "SARS-Cov2019*" or "SARSCov-2019*" or "SARS-Cov-2019*" or SARS2* or "SARS-2*" or SARScoronavirus2* or "SARS-coronavirus-2*" or "SARScoronavirus 2*" or "SARS coronavirus2*" or SARScoronovirus2* or "SARS-coronovirus-2*" or "SARScoronovirus 2*" or "SARS coronovirus2*" or "severe acute respiratory syndrome*")):ti,ab | 4 |
| #26 | ((convalescen* or recuperat* or after* or followup* or "follow up*" or rehab*) near/1 (therap* or care*) near/10 (covid* or coronavirus* or "corona* virus*" or coronovirus* or "corono* virus*" or coronavirinae* or "corona* virinae*" or Cov or "2019-nCoV*" or 2019nCoV* or "19-nCoV*" or 19nCoV* or nCoV2019* or "nCoV-2019*" or nCoV19* or "nCoV-19*" or "HCoV-19*" or HCoV19* or "HCoV-2019*" or HCoV2019* or "2019 novel*" or Ncov* or "n-cov" or "SARS-CoV-2*" or "SARSCoV-2*" or "SARSCoV2*" or "SARS-CoV2*" or SARSCov19* or "SARS-Cov19*" or "SARSCov-19*" or "SARS-Cov-19*" or SARSCov2019* or "SARS-Cov2019*" or "SARSCov-2019*" or "SARS-Cov-2019*" or SARS2* or "SARS-2*" or SARScoronavirus2* or "SARS-coronavirus-2*" or "SARScoronavirus 2*" or "SARS coronavirus2*" or SARScoronovirus2* or "SARS-coronovirus-2*" or "SARScoronovirus 2*" or "SARS coronovirus2*" or "severe acute respiratory syndrome*")):ti,ab | 14 |
| #27 | ((ongoing* or endur* or long* or legacy* or slow* or gradual* or protract* or lengthy* or chronic* or persist* or relaps* or remit* or remission* or residual* or delay* or prolong* or extend* or linger* or permanent* or fluctuat* or multisystem* or "multi system*" or nonrecover* or "non recovery" or "non recovering*" or subacute* or "sub acute*" or lasting* or continuous* or continual* or continuing* or postacute* or "post acute*" or postdischarg* or "post discharge" or "post discharging" or postinfect* or "post infection*" or "post infective*" or postviral* or "post viral*" or postvirus* or "post virus*" or "medium* term*" or mediumterm* or adverse* or dangerous* or harmful* or indirect* or injurious* or secondary* or side effect* or undesirable* or sequela* or complication* or consequence* or effect* or event* or impact* or outcome* or reaction* or complexit* or aftercare* or impair* or problem* or issue* or rehab* or function* or perform*) near/10 ((daily* or everyday* or day* or normal* or usual*) near/1 (activit* or living* or life* or lives* or job* or work* or employ* or occupation* or hobby* or hobbies* or leisure*)) near/10 (covid* or coronavirus* or "corona* virus*" or coronovirus* or "corono* virus*" or coronavirinae* or "corona* virinae*" or Cov or "2019-nCoV*" or 2019nCoV* or "19-nCoV*" or 19nCoV* or nCoV2019* or "nCoV-2019*" or nCoV19* or "nCoV-19*" or "HCoV-19*" or HCoV19* or "HCoV-2019*" or HCoV2019* or "2019 novel*" or Ncov* or "n-cov" or "SARS-CoV-2*" or "SARSCoV-2*" or "SARSCoV2*" or "SARS-CoV2*" or SARSCov19* or "SARS-Cov19*" or "SARSCov-19*" or "SARS-Cov-19*" or SARSCov2019* or "SARS-Cov2019*" or "SARSCov-2019*" or "SARS-Cov-2019*" or SARS2* or "SARS-2*" or SARScoronavirus2* or "SARS-coronavirus-2*" or "SARScoronavirus 2*" or "SARS coronavirus2*" or SARScoronovirus2* or "SARS-coronovirus-2*" or "SARScoronovirus 2*" or "SARS coronovirus2*" or "severe acute respiratory syndrome*")):ti,ab | 25 |
| #28 | ((ongoing* or endur* or long* or legacy* or slow* or gradual* or protract* or lengthy* or chronic* or persist* or relaps* or remit* or remission* or residual* or delay* or prolong* or extend* or linger* or permanent* or fluctuat* or multisystem* or "multi system*" or nonrecover* or "non recovery" or "non recovering" or subacute* or "sub acute*" or lasting* or continuous* or continual* or continuing* or postacute* or "post acute*" or postdischarg* or "post discharge" or "post discharging" or postinfect* or "post infection*" or "post infective*" or postviral* or "post viral*" or postvirus* or "post virus*" or "medium* term*" or mediumterm*) near/3 ((health* or adverse* or dangerous* or harmful* or indirect* or injurious* or secondary* or side* or undesirable* or negative* or damaging* or detriment* or abnormal*) near/1 (sequela* or complication* or consequence* or effect* or event* or impact* or outcome* or reaction* or complexit* or aftercare* or impair* or problem* or issue* or symptom* or disorder*)) near/10 (covid* or coronavirus* or "corona* virus*" or coronovirus* or "corono* virus*" or coronavirinae* or "corona* virinae*" or Cov or "2019-nCoV*" or 2019nCoV* or "19-nCoV*" or 19nCoV* or nCoV2019* or "nCoV-2019*" or nCoV19* or "nCoV-19*" or "HCoV-19*" or HCoV19* or "HCoV-2019*" or HCoV2019* or "2019 novel*" or Ncov* or "n-cov" or "SARS-CoV-2*" or "SARSCoV-2*" or "SARSCoV2*" or "SARS-CoV2*" or SARSCov19* or "SARS-Cov19*" or "SARSCov-19*" or "SARS-Cov-19*" or SARSCov2019* or "SARS-Cov2019*" or "SARSCov-2019*" or "SARS-Cov-2019*" or SARS2* or "SARS-2*" or SARScoronavirus2* or "SARS-coronavirus-2*" or "SARScoronavirus 2*" or "SARS coronavirus2*" or SARScoronovirus2* or "SARS-coronovirus-2*" or "SARScoronovirus 2*" or "SARS coronovirus2*" or "severe acute respiratory syndrome*")):ti,ab | 17 |
| #29 | ((ongoing* or endur* or long* or legacy* or slow* or gradual* or protract* or lengthy* or chronic* or persist* or relaps* or remit* or remission* or residual* or delay* or prolong* or extend* or linger* or permanent* or fluctuat* or multisystem* or "multi system*" or nonrecover* or "non recovery" or "non recovering" or subacute* or "sub acute*" or lasting* or continuous* or continual* or continuing* or postacute* or "post acute*" or postdischarg* or "post discharge" or "post discharging" or postinfect* or "post infection*" or "post infective*" or postviral* or "post viral*" or postvirus* or "post virus*" or "medium* term*" or mediumterm*) near/3 ((physiolog* or neuro* or cardio* or gastro* or musculo* or renal* or kidney* or cognitive* or cognition* or rheumato* or dermatol* or skin* or haematol* or blood* or autonomic* or nervous* or nervous system* or otolaryngol* or laryngol* or otolog* or cerebro* or brain* or vascular* or respirator* or lung* or pulmonary* or psycholog* or mental health* or mental* or psychiatr* or exertion* or debilit* or devitali* or enervat* or drain* or sleep* or weak* or tired* or frail* or sapp* or strength* or confusion* or letharg* or fatigue* or tired* or weariness* or exhaust* or malaise* or pain* or headache* or breathless* or breathing* or myalgia* or delirious* or delirium* or appetite* or muscle* or muscular* or fitness* or memory* or stress* or depress* or anxiety* or emotion* or cough* or fever* or temperatur* or pneumon* or conjunctivit* or throat* or pharyngit* or dyspnea* or dyspnoea* or sick* or nausea* or nauseous* or vomit* or diarrhoea* or diarrhea* or taste* or anosmia* or smell* or olfact* or sweat* or dehydrat* or pyrexi* or nasal* or nose* or mucus* or ear* or hearing* or deaf* or "brain fog*" or cardiac* or thoracic* or chest* or ischemic* or ischaemic* or heart* or liver* or hepatic* or immuno* or palpitation* or vertigo* or metabol* or vestibular* or endocrine* or encephalit*) near/1 (sequela* or complication* or consequence* or effect* or event* or impact* or outcome* or reaction* or complexit* or aftercare* or impair* or problem* or issue* or symptom* or disorder* or abnormal*)) near/10 (covid* or coronavirus* or "corona* virus*" or coronovirus* or "corono* virus*" or coronavirinae* or "corona* virinae*" or Cov or "2019-nCoV*" or 2019nCoV* or "19-nCoV*" or 19nCoV* or nCoV2019* or "nCoV-2019*" or nCoV19* or "nCoV-19*" or "HCoV-19*" or HCoV19* or "HCoV-2019*" or HCoV2019* or "2019 novel*" or Ncov* or "n-cov" or "SARS-CoV-2*" or "SARSCoV-2*" or "SARSCoV2*" or "SARS-CoV2*" or SARSCov19* or "SARS-Cov19*" or "SARSCov-19*" or "SARS-Cov-19*" or SARSCov2019* or "SARS-Cov2019*" or "SARSCov-2019*" or "SARS-Cov-2019*" or SARS2* or "SARS-2*" or SARScoronavirus2* or "SARS-coronavirus-2*" or "SARScoronavirus 2*" or "SARS coronavirus2*" or SARScoronovirus2* or "SARS-coronovirus-2*" or "SARScoronovirus 2*" or "SARS coronovirus2*" or "severe acute respiratory syndrome*")):ti,ab | 48 |
| #30 | ((physiolog* or neuro* or cardio* or gastro* or musculo* or renal* or kidney* or cognitive* or cognition* or rheumato* or dermatol* or skin* or haematol* or blood* or autonomic* or nervous* or "nervous system*" or otolaryngol* or laryngol* or otolog* or cerebro* or brain* or vascular* or respirator* or lung* or pulmonary* or psycholog* or "mental health*" or mental* or psychiatr* or exertion* or debilit* or devitali* or enervat* or drain* or sleep* or weak* or tired* or frail* or sapp* or strength* or confusion* or letharg* or fatigue* or tired* or weariness* or exhaust* or malaise* or pain* or headache* or breathless* or breathing* or myalgia* or delirious* or delirium* or appetite* or muscle* or muscular* or fitness* or memory* or stress* or depress* or anxiety* or emotion* or cough* or fever* or temperatur* or pneumon* or conjunctivit* or throat* or pharyngit* or dyspnea* or dyspnoea* or sick* or nausea* or nauseous* or vomit* or diarrhoea* or diarrhea* or taste* or anosmia* or smell* or olfact* or sweat* or dehydrat* or pyrexi* or nasal* or nose* or mucus* or ear* or hearing* or deaf* or "brain fog*" or cardiac* or thoracic* or chest* or ischemic* or ischaemic* or heart* or liver* or hepatic* or immuno* or palpitation* or vertigo* or metabol* or vestibular* or endocrine* or encephalit*) near/3 (postcovid* or "post covid*" or postcoronavirus* or "postcorona* virus*" or "post coronavirus*" or "post corona* virus*" or postcoronovirus* or "postcorono* virus*" or "post coronovirus*" or "post corono* virus*" or postcoronavirinae* or "postcorona* virinae*" or "post coronavirinae*" or "post corona* virinae*" or postCov or "post Cov" or postsars* or "post sars*" or "post severe acute respiratory syndrome*" or postncov* or "post ncov*" or posthcov* or "post hcov*")):ti,ab | 71 |
| #31 | ((physiolog* or neuro* or cardio* or gastro* or musculo* or renal* or kidney* or cognitive* or cognition* or rheumato* or dermatol* or skin* or haematol* or blood* or autonomic* or nervous* or nervous system* or otolaryngol* or laryngol* or otolog* or cerebro* or brain* or vascular* or respirator* or lung* or pulmonary* or psycholog* or mental health* or mental* or psychiatr* or exertion* or debilit* or devitali* or enervat* or drain* or sleep* or weak* or tired* or frail* or sapp* or strength* or confusion* or letharg* or fatigue* or tired* or weariness* or exhaust* or malaise* or pain* or headache* or breathless* or breathing* or myalgia* or delirious* or delirium* or appetite* or muscle* or muscular* or fitness* or memory* or stress* or depress* or anxiety* or emotion* or cough* or fever* or temperatur* or pneumon* or conjunctivit* or throat* or pharyngit* or dyspnea* or dyspnoea* or sick* or nausea* or nauseous* or vomit* or diarrhoea* or diarrhea* or taste* or anosmia* or smell* or olfact* or sweat* or dehydrat* or pyrexi* or nasal* or nose* or mucus* or ear* or hearing* or deaf* or "brain fog*" or cardiac* or thoracic* or chest* or ischemic* or ischaemic* or heart* or liver* or hepatic* or immuno* or palpitation* or vertigo* or metabol* or vestibular* or endocrine* or encephalit*) near/1 (sequela* or complication* or consequence* or complexit*) near/10 (covid* or coronavirus* or "corona* virus*" or coronovirus* or "corono* virus*" or coronavirinae* or "corona* virinae*" or Cov or "2019-nCoV*" or 2019nCoV* or "19-nCoV*" or 19nCoV* or nCoV2019* or "nCoV-2019*" or nCoV19* or "nCoV-19*" or "HCoV-19*" or HCoV19* or "HCoV-2019*" or HCoV2019* or "2019 novel*" or Ncov* or "n-cov" or "SARS-CoV-2*" or "SARSCoV-2*" or "SARSCoV2*" or "SARS-CoV2*" or SARSCov19* or "SARS-Cov19*" or "SARSCov-19*" or "SARS-Cov-19*" or SARSCov2019* or "SARS-Cov2019*" or "SARSCov-2019*" or "SARS-Cov-2019*" or SARS2* or "SARS-2*" or SARScoronavirus2* or "SARS-coronavirus-2*" or "SARScoronavirus 2*" or "SARS coronavirus2*" or SARScoronovirus2* or "SARS-coronovirus-2*" or "SARScoronovirus 2*" or "SARS coronovirus2*" or "severe acute respiratory syndrome*")):ti,ab | 89 |
| #32 | (((((multi or multiple* or overlap* or cluster* or numerous* or varied* or variety*) near/1 (symptom* or system* or disease* or disorder* or illness* or condition* or syndrome*)) or (multisymptom* or multisystem* or multidisease* or multidisorder* or multiillness* or multicondition* or multisyndrom*)) near/10 (covid* or coronavirus* or "corona* virus*" or coronovirus* or "corono* virus*" or coronavirinae* or "corona* virinae*" or Cov or "2019-nCoV*" or 2019nCoV* or "19-nCoV*" or 19nCoV* or nCoV2019* or "nCoV-2019*" or nCoV19* or "nCoV-19*" or "HCoV-19*" or HCoV19* or "HCoV-2019*" or HCoV2019* or "2019 novel*" or Ncov* or "n-cov" or "SARS-CoV-2*" or "SARSCoV-2*" or "SARSCoV2*" or "SARS-CoV2*" or SARSCov19* or "SARS-Cov19*" or "SARSCov-19*" or "SARS-Cov-19*" or SARSCov2019* or "SARS-Cov2019*" or "SARSCov-2019*" or "SARS-Cov-2019*" or SARS2* or "SARS-2*" or SARScoronavirus2* or "SARS-coronavirus-2*" or "SARScoronavirus 2*" or "SARS coronavirus2*" or SARScoronovirus2* or "SARS-coronovirus-2*" or "SARScoronovirus 2*" or "SARS coronovirus2*" or "severe acute respiratory syndrome*")) not ("Multisystem* inflammatory* syndrome*" or "inflammatory* multisystem* syndrome*")):ti,ab | 15 |
| #33 | {or #23-#32} | 263 |
| #34 | #7 or #20 or #33 | 542 |

NOTE: the strategy could not be run as a whole as it would time out on the Wiley platform. The individual lines had to be run separately in order to obtain the results. Registry entry records were not added to the search results.

## Web of Science Core Collection

| Platform | Clarivate  Advanced search |
| --- | --- |
| Search date | 09/05/2022 |
| No. of results | 11877 |

|  |  | Filter | No. of results |
| --- | --- | --- | --- |
| #1 | TS=(longcovid* or “long covid*” or longcoronavirus* or “longcorona* virus*” or “long coronavirus*” or “long corona* virus*” or longcoronovirus* or “longcorono* virus*” or “long coronovirus*” or “long corono* virus*” or longcoronavirinae* or “longcorona* virinae*” or “long coronavirinae*” or “long corona* virinae*” or longCov or “long Cov” or longsars* or “long sars*” or "long severe acute respiratory syndrome*" or longncov* or “long ncov*” or longhcov* or “long hcov*”) |  | 1050 |
| #2 | TS=((long* or endur* or legacy* or slow* or gradual* or protract* or lengthy* or chronic* or persist* or relaps* or remit* or remission* or residual* or delay* or prolong* or extend* or linger* or permanent* or fluctuat* or sequela* or multisystem* or "multi system*" or nonrecover* or "non recover*" or subacute* or "sub acute*" or lasting* or continuous* or continual* or continuing* or postacute* or "post acute*" or postdischarg* or "post discharg*" or postinfect* or "post infect*" or postviral* or "post viral*" or postvirus* or "post virus*") NEAR/1 (covid* or coronavirus* or “corona* virus*” or coronovirus* or “corono* virus*” or coronavirinae* or “corona* virinae*” or Cov or "2019-nCoV*" or 2019nCoV* or "19-nCoV*" or 19nCoV* or nCoV2019* or "nCoV-2019*" or nCoV19* or "nCoV-19*" or "HCoV-19*" or HCoV19* or "HCoV-2019*" or HCoV2019* or "2019 novel*" or Ncov* or "n-cov" or "SARS-CoV-2*" or "SARSCoV-2*" or "SARSCoV2*" or "SARS-CoV2*" or SARSCov19* or "SARS-Cov19*" or "SARSCov-19*" or "SARS-Cov-19*" or SARSCov2019* or "SARS-Cov2019*" or "SARSCov-2019*" or "SARS-Cov-2019*" or SARS2* or "SARS-2*" or SARScoronavirus2* or "SARS-coronavirus-2*" or "SARScoronavirus 2*" or "SARS coronavirus2*" or SARScoronovirus2* or "SARS-coronovirus-2*" or "SARScoronovirus 2*" or "SARS coronovirus2*" or "severe acute respiratory syndrome*")) |  | 3970 |
| #3 | TS=(("long* term*" or longterm* or "long* haul*" or longhaul* or "long* tail*" or longtail* or longduration* or "long duration*" or longlast* or "long last*" or longstanding* or "long standing*" or "medium* term*" or mediumterm*) NEAR/3 (covid* or coronavirus* or “corona* virus*” or coronovirus* or “corono* virus*” or coronavirinae* or “corona* virinae*” or Cov or "2019-nCoV*" or 2019nCoV* or "19-nCoV*" or 19nCoV* or nCoV2019* or "nCoV-2019*" or nCoV19* or "nCoV-19*" or "HCoV-19*" or HCoV19* or "HCoV-2019*" or HCoV2019* or "2019 novel*" or Ncov* or "n-cov" or "SARS-CoV-2*" or "SARSCoV-2*" or "SARSCoV2*" or "SARS-CoV2*" or SARSCov19* or "SARS-Cov19*" or "SARSCov-19*" or "SARS-Cov-19*" or SARSCov2019* or "SARS-Cov2019*" or "SARSCov-2019*" or "SARS-Cov-2019*" or SARS2* or "SARS-2*" or SARScoronavirus2* or "SARS-coronavirus-2*" or "SARScoronavirus 2*" or "SARS coronavirus2*" or SARScoronovirus2* or "SARS-coronovirus-2*" or "SARScoronovirus 2*" or "SARS coronovirus2*" or "severe acute respiratory syndrome*")) |  | 2036 |
| #4 | TS=((postcovid* or “post covid*” or postcoronavirus* or “postcorona* virus*” or “post coronavirus*” or “post corona* virus*” or postcoronovirus* or “postcorono* virus*” or “post coronovirus*” or “post corono* virus*” or postcoronavirinae* or “postcorona* virinae*” or “post coronavirinae”* or “post corona* virinae*” or postCov or “post Cov” or postsars* or “post sars*” or "post severe acute respiratory syndrome*" or postncov* or “post ncov*” or posthcov* or “post hcov*”) NEAR/3 (syndrome* or disorder* or illness* or sickness* or disease* or condition* or symptom* or sign* or prognos* or followup* or "follow up*" or feature* or comorbid* or "co morbid*" or multimorbid* or "multi morbid*" or survivor* or survival* or risk* or care* or convalescen* or recuperat* or aftercare* or ambulatory* or outpatient* or "out patient*")) |  | 1089 |
| #5 | TS=((ongoing* or long* or endur* or legacy* or slow* or gradual* or protract* or lengthy* or chronic* or persist* or relaps* or remit* or remission* or residual* or delay* or prolong* or extend* or linger* or permanent* or fluctuat* or multisystem* or "multi system*" or nonrecover* or "non recover*" or subacute* or "sub acute*" or lasting* or continuous* or continual* or continuing* or postacute* or "post acute*" or postdischarg* or "post discharg*" or postinfect* or "post infect*" or postviral* or "post viral*" or postvirus* or "post virus*" or "medium* term*" or mediumterm*) NEAR/4 (sequela* or illness* or symptom* or sign* or prognos* or rehab* or convalescen* or recuperat* or followup* or "follow up*" or feature*) NEAR/10 (covid* or coronavirus* or “corona* virus*” or coronovirus* or “corono* virus*” or coronavirinae* or “corona* virinae*” or Cov or "2019-nCoV*" or 2019nCoV* or "19-nCoV*" or 19nCoV* or nCoV2019* or "nCoV-2019*" or nCoV19* or "nCoV-19*" or "HCoV-19*" or HCoV19* or "HCoV-2019*" or HCoV2019* or "2019 novel*" or Ncov* or "n-cov" or "SARS-CoV-2*" or "SARSCoV-2*" or "SARSCoV2*" or "SARS-CoV2*" or SARSCov19* or "SARS-Cov19*" or "SARSCov-19*" or "SARS-Cov-19*" or SARSCov2019* or "SARS-Cov2019*" or "SARSCov-2019*" or "SARS-Cov-2019*" or SARS2* or "SARS-2*" or SARScoronavirus2* or "SARS-coronavirus-2*" or "SARScoronavirus 2*" or "SARS coronavirus2*" or SARScoronovirus2* or "SARS-coronovirus-2*" or "SARScoronovirus 2*" or "SARS coronovirus2*" or "severe acute respiratory syndrome*")) |  | 2970 |
| #6 | TS=((ongoing* or long* or endur* or legacy* or slow* or gradual* or protract* or lengthy* or chronic* or persist* or relaps* or remit* or remission* or residual* or delay* or prolong* or extend* or linger* or permanent* or fluctuat* or multisystem* or "multi system*" or subacute* or "sub acute*" or lasting* or continuous* or continual* or continuing* or post* or after* or follow* or "medium* term*" or mediumterm*) NEAR/1 recover* NEAR/10 (covid* or coronavirus* or “corona* virus*” or coronovirus* or “corono* virus*” or coronavirinae* or “corona* virinae*” or Cov or "2019-nCoV*" or 2019nCoV* or "19-nCoV*" or 19nCoV* or nCoV2019* or "nCoV-2019*" or nCoV19* or "nCoV-19*" or "HCoV-19*" or HCoV19* or "HCoV-2019*" or HCoV2019* or "2019 novel*" or Ncov* or "n-cov" or "SARS-CoV-2*" or "SARSCoV-2*" or "SARSCoV2*" or "SARS-CoV2*" or SARSCov19* or "SARS-Cov19*" or "SARSCov-19*" or "SARS-Cov-19*" or SARSCov2019* or "SARS-Cov2019*" or "SARSCov-2019*" or "SARS-Cov-2019*" or SARS2* or "SARS-2*" or SARScoronavirus2* or "SARS-coronavirus-2*" or "SARScoronavirus 2*" or "SARS coronavirus2*" or SARScoronovirus2* or "SARS-coronovirus-2*" or "SARScoronovirus 2*" or "SARS coronovirus2*" or "severe acute respiratory syndrome*")) |  | 1093 |
| #7 | TS=((recover* or nonrecover*) NEAR/3 function* NEAR/10 (covid* or coronavirus* or “corona* virus*” or coronovirus* or “corono* virus*” or coronavirinae* or “corona* virinae*” or Cov or "2019-nCoV*" or 2019nCoV* or "19-nCoV*" or 19nCoV* or nCoV2019* or "nCoV-2019*" or nCoV19* or "nCoV-19*" or "HCoV-19*" or HCoV19* or "HCoV-2019*" or HCoV2019* or "2019 novel*" or Ncov* or "n-cov" or "SARS-CoV-2*" or "SARSCoV-2*" or "SARSCoV2*" or "SARS-CoV2*" or SARSCov19* or "SARS-Cov19*" or "SARSCov-19*" or "SARS-Cov-19*" or SARSCov2019* or "SARS-Cov2019*" or "SARSCov-2019*" or "SARS-Cov-2019*" or SARS2* or "SARS-2*" or SARScoronavirus2* or "SARS-coronavirus-2*" or "SARScoronavirus 2*" or "SARS coronavirus2*" or SARScoronovirus2* or "SARS-coronovirus-2*" or "SARScoronovirus 2*" or "SARS coronovirus2*" or "severe acute respiratory syndrome*")) |  | 111 |
| #8 | TS=((postacute* or "post acute*" or postdischarg* or "post discharg*" or postinfect* or "post infect*" or postviral* or "post viral*" or postvirus* or "post virus*" or subacute* or "sub acute*") NEAR/3 (care* or convalescen* or recuperat* or aftercare* or ambulatory* or outpatient* or "out patient*" or survivor* or survival*) NEAR/10 (covid* or coronavirus* or “corona* virus*” or coronovirus* or “corono* virus*” or coronavirinae* or “corona* virinae*” or Cov or "2019-nCoV*" or 2019nCoV* or "19-nCoV*" or 19nCoV* or nCoV2019* or "nCoV-2019*" or nCoV19* or "nCoV-19*" or "HCoV-19*" or HCoV19* or "HCoV-2019*" or HCoV2019* or "2019 novel*" or Ncov* or "n-cov" or "SARS-CoV-2*" or "SARSCoV-2*" or "SARSCoV2*" or "SARS-CoV2*" or SARSCov19* or "SARS-Cov19*" or "SARSCov-19*" or "SARS-Cov-19*" or SARSCov2019* or "SARS-Cov2019*" or "SARSCov-2019*" or "SARS-Cov-2019*" or SARS2* or "SARS-2*" or SARScoronavirus2* or "SARS-coronavirus-2*" or "SARScoronavirus 2*" or "SARS coronavirus2*" or SARScoronovirus2* or "SARS-coronovirus-2*" or "SARScoronovirus 2*" or "SARS coronovirus2*" or "severe acute respiratory syndrome*")) |  | 99 |
| #9 | TS=((convalescen* or recuperat* or after* or followup* or "follow up*" or rehab*) NEAR/1 (therap* or care*) NEAR/10 (covid* or coronavirus* or “corona* virus*” or coronovirus* or “corono* virus*” or coronavirinae* or “corona* virinae*” or Cov or "2019-nCoV*" or 2019nCoV* or "19-nCoV*" or 19nCoV* or nCoV2019* or "nCoV-2019*" or nCoV19* or "nCoV-19*" or "HCoV-19*" or HCoV19* or "HCoV-2019*" or HCoV2019* or "2019 novel*" or Ncov* or "n-cov" or "SARS-CoV-2*" or "SARSCoV-2*" or "SARSCoV2*" or "SARS-CoV2*" or SARSCov19* or "SARS-Cov19*" or "SARSCov-19*" or "SARS-Cov-19*" or SARSCov2019* or "SARS-Cov2019*" or "SARSCov-2019*" or "SARS-Cov-2019*" or SARS2* or "SARS-2*" or SARScoronavirus2* or "SARS-coronavirus-2*" or "SARScoronavirus 2*" or "SARS coronavirus2*" or SARScoronovirus2* or "SARS-coronovirus-2*" or "SARScoronovirus 2*" or "SARS coronovirus2*" or "severe acute respiratory syndrome*")) |  | 650 |
| #10 | TS=((ongoing* or endur* or long* or legacy* or slow* or gradual* or protract* or lengthy* or chronic* or persist* or relaps* or remit* or remission* or residual* or delay* or prolong* or extend* or linger* or permanent* or fluctuat* or multisystem* or "multi system*" or nonrecover* or "non recover*" or subacute* or "sub acute*" or lasting* or continuous* or continual* or continuing* or postacute* or "post acute*" or postdischarg* or "post discharg*" or postinfect* or "post infect*" or postviral* or "post viral*" or postvirus* or "post virus*" or "medium* term*" or mediumterm* or adverse* or dangerous* or harmful* or indirect* or injurious* or secondary* or “side effect*” or undesirable* or sequela* or complication* or consequence* or effect* or event* or impact* or outcome* or reaction* or complexit* or aftercare* or impair* or problem* or issue* or rehab* or function* or perform*) NEAR/10 ((daily* or everyday* or day* or normal* or usual*) NEAR/1 (activit* or living* or life* or lives* or job* or work* or employ* or occupation* or hobby* or hobbies* or leisure*)) NEAR/10 (covid* or coronavirus* or “corona* virus*” or coronovirus* or “corono* virus*” or coronavirinae* or “corona* virinae*” or Cov or "2019-nCoV*" or 2019nCoV* or "19-nCoV*" or 19nCoV* or nCoV2019* or "nCoV-2019*" or nCoV19* or "nCoV-19*" or "HCoV-19*" or HCoV19* or "HCoV-2019*" or HCoV2019* or "2019 novel*" or Ncov* or "n-cov" or "SARS-CoV-2*" or "SARSCoV-2*" or "SARSCoV2*" or "SARS-CoV2*" or SARSCov19* or "SARS-Cov19*" or "SARSCov-19*" or "SARS-Cov-19*" or SARSCov2019* or "SARS-Cov2019*" or "SARSCov-2019*" or "SARS-Cov-2019*" or SARS2* or "SARS-2*" or SARScoronavirus2* or "SARS-coronavirus-2*" or "SARScoronavirus 2*" or "SARS coronavirus2*" or SARScoronovirus2* or "SARS-coronovirus-2*" or "SARScoronovirus 2*" or "SARS coronovirus2*" or "severe acute respiratory syndrome*")) |  | 680 |
| #11 | TS=((ongoing* or endur* or long* or legacy* or slow* or gradual* or protract* or lengthy* or chronic* or persist* or relaps* or remit* or remission* or residual* or delay* or prolong* or extend* or linger* or permanent* or fluctuat* or multisystem* or "multi system*" or nonrecover* or "non recover*" or subacute* or "sub acute*" or lasting* or continuous* or continual* or continuing* or postacute* or "post acute*" or postdischarg* or "post discharg*" or postinfect* or "post infect*" or postviral* or "post viral*" or postvirus* or "post virus*" or "medium* term*" or mediumterm*) NEAR/3 ((health* or adverse* or dangerous* or harmful* or indirect* or injurious* or secondary* or side* or undesirable* or negative* or damaging* or detriment* or abnormal*) NEAR/1 (sequela* or complication* or consequence* or effect* or event* or impact* or outcome* or reaction* or complexit* or aftercare* or impair* or problem* or issue* or symptom* or disorder*)) NEAR/10 (covid* or coronavirus* or “corona* virus*” or coronovirus* or “corono* virus*” or coronavirinae* or “corona* virinae*” or Cov or "2019-nCoV*" or 2019nCoV* or "19-nCoV*" or 19nCoV* or nCoV2019* or "nCoV-2019*" or nCoV19* or "nCoV-19*" or "HCoV-19*" or HCoV19* or "HCoV-2019*" or HCoV2019* or "2019 novel*" or Ncov* or "n-cov" or "SARS-CoV-2*" or "SARSCoV-2*" or "SARSCoV2*" or "SARS-CoV2*" or SARSCov19* or "SARS-Cov19*" or "SARSCov-19*" or "SARS-Cov-19*" or SARSCov2019* or "SARS-Cov2019*" or "SARSCov-2019*" or "SARS-Cov-2019*" or SARS2* or "SARS-2*" or SARScoronavirus2* or "SARS-coronavirus-2*" or "SARScoronavirus 2*" or "SARS coronavirus2*" or SARScoronovirus2* or "SARS-coronovirus-2*" or "SARScoronovirus 2*" or "SARS coronovirus2*" or "severe acute respiratory syndrome*")) |  | 383 |
| #12 | TS=((ongoing* or endur* or long* or legacy* or slow* or gradual* or protract* or lengthy* or chronic* or persist* or relaps* or remit* or remission* or residual* or delay* or prolong* or extend* or linger* or permanent* or fluctuat* or multisystem* or "multi system*" or nonrecover* or "non recover*" or subacute* or "sub acute*" or lasting* or continuous* or continual* or continuing* or postacute* or "post acute*" or postdischarg* or "post discharg*" or postinfect* or "post infect*" or postviral* or "post viral*" or postvirus* or "post virus*" or "medium* term*" or mediumterm*) NEAR/3 ((physiolog* or neuro* or cardio* or gastro* or musculo* or renal* or kidney* or cognitive* or cognition* or rheumato* or dermatol* or skin* or haematol* or blood* or autonomic* or nervous* or “nervous system*” or otolaryngol* or laryngol* or otolog* or cerebro* or brain* or vascular* or respirator* or lung* or pulmonary* or psycholog* or “mental health*” or mental* or psychiatr* or exertion* or debilit* or devitali* or enervat* or drain* or sleep* or weak* or tired* or frail* or sapp* or strength* or confusion* or letharg* or fatigue* or tired* or weariness* or exhaust* or malaise* or pain* or headache* or breathless* or breathing* or myalgia* or delirious* or delirium* or appetite* or muscle* or muscular* or fitness* or memory* or stress* or depress* or anxiety* or emotion* or cough* or fever* or temperatur* or pneumon* or conjunctivit* or throat* or pharyngit* or dyspnea* or dyspnoea* or sick* or nausea* or nauseous* or vomit* or diarrhoea* or diarrhea* or taste* or anosmia* or smell* or olfact* or sweat* or dehydrat* or pyrexi* or nasal* or nose* or mucus* or ear* or hearing* or deaf* or "brain fog*" or cardiac* or thoracic* or chest* or ischemic* or ischaemic* or heart* or liver* or hepatic* or immuno* or palpitation* or vertigo* or metabol* or vestibular* or endocrine* or encephalit* or physical* or cough* or fibrosis* or myocarditis* or Guillain* or barre* or neuralgi* or amyotroph* or thrombo* or clot* or rash* or hive* or urticari* or lymph* or stroke* or TIA or toe* or foot* or feet* or finger* or chilblain* or numb* or inflammat* or inflame* or arthralgi* or eye* or organ or organs or tingl* or sting* or burn* or bladder* or urogenit* or genitourin* or genital* or reproducti* or urinary* or joint* or tachycard* or atrial* or autoimmun* or dysautonomi* or polyneuro* or mast* or mobility* or walking* or ambulation* or energy*) NEAR/1 (sequela* or complication* or consequence* or effect* or event* or impact* or outcome* or reaction* or complexit* or aftercare* or impair* or problem* or issue* or symptom* or disorder* or abnormal*)) NEAR/10 (covid* or coronavirus* or “corona* virus*” or coronovirus* or “corono* virus*” or coronavirinae* or “corona* virinae*” or Cov or "2019-nCoV*" or 2019nCoV* or "19-nCoV*" or 19nCoV* or nCoV2019* or "nCoV-2019*" or nCoV19* or "nCoV-19*" or "HCoV-19*" or HCoV19* or "HCoV-2019*" or HCoV2019* or "2019 novel*" or Ncov* or "n-cov" or "SARS-CoV-2*" or "SARSCoV-2*" or "SARSCoV2*" or "SARS-CoV2*" or SARSCov19* or "SARS-Cov19*" or "SARSCov-19*" or "SARS-Cov-19*" or SARSCov2019* or "SARS-Cov2019*" or "SARSCov-2019*" or "SARS-Cov-2019*" or SARS2* or "SARS-2*" or SARScoronavirus2* or "SARS-coronavirus-2*" or "SARScoronavirus 2*" or "SARS coronavirus2*" or SARScoronovirus2* or "SARS-coronovirus-2*" or "SARScoronovirus 2*" or "SARS coronovirus2*" or "severe acute respiratory syndrome*")) |  | 1012 |
| #13 | TS=((physiolog* or neuro* or cardio* or gastro* or musculo* or renal* or kidney* or cognitive* or cognition* or rheumato* or dermatol* or skin* or haematol* or blood* or autonomic* or nervous* or “nervous system*” or otolaryngol* or laryngol* or otolog* or cerebro* or brain* or vascular* or respirator* or lung* or pulmonary* or psycholog* or “mental health*” or mental* or psychiatr* or exertion* or debilit* or devitali* or enervat* or drain* or sleep* or weak* or tired* or frail* or sapp* or strength* or confusion* or letharg* or fatigue* or tired* or weariness* or exhaust* or malaise* or pain* or headache* or breathless* or breathing* or myalgia* or delirious* or delirium* or appetite* or muscle* or muscular* or fitness* or memory* or stress* or depress* or anxiety* or emotion* or cough* or fever* or temperatur* or pneumon* or conjunctivit* or throat* or pharyngit* or dyspnea* or dyspnoea* or sick* or nausea* or nauseous* or vomit* or diarrhoea* or diarrhea* or taste* or anosmia* or smell* or olfact* or sweat* or dehydrat* or pyrexi* or nasal* or nose* or mucus* or ear* or hearing* or deaf* or "brain fog*" or cardiac* or thoracic* or chest* or ischemic* or ischaemic* or heart* or liver* or hepatic* or immuno* or palpitation* or vertigo* or metabol* or vestibular* or endocrine* or encephalit* or physical* or cough* or fibrosis* or myocarditis* or Guillain* or barre* or neuralgi* or amyotroph* or thrombo* or clot* or rash* or hive* or urticari* or lymph* or stroke* or TIA or toe* or foot* or feet* or finger* or chilblain* or numb* or inflammat* or inflame* or arthralgi* or eye* or organ or organs or tingl* or sting* or burn* or bladder* or urogenit* or genitourin* or genital* or reproducti* or urinary* or joint* or tachycard* or atrial* or autoimmun* or dysautonomi* or polyneuro* or mast* or mobility* or walking* or ambulation* or energy*) NEAR/3 (postcovid* or “post covid*” or postcoronavirus* or “postcorona* virus*” or “post coronavirus*” or “post corona* virus*” or postcoronovirus* or “postcorono* virus*” or “post coronovirus*” or “post corono* virus*” or postcoronavirinae* or “postcorona* virinae*” or “post coronavirinae*” or “post corona* virinae*” or postCov or “post Cov” or postsars* or “post sars*” or "post severe acute respiratory syndrome*" or postncov* or “post ncov*” or posthcov* or “post hcov*”)) |  | 956 |
| #14 | TS=((physiolog* or neuro* or cardio* or gastro* or musculo* or renal* or kidney* or cognitive* or cognition* or rheumato* or dermatol* or skin* or haematol* or blood* or autonomic* or nervous* or “nervous system*” or otolaryngol* or laryngol* or otolog* or cerebro* or brain* or vascular* or respirator* or lung* or pulmonary* or psycholog* or “mental health*” or mental* or psychiatr* or exertion* or debilit* or devitali* or enervat* or drain* or sleep* or weak* or tired* or frail* or sapp* or strength* or confusion* or letharg* or fatigue* or tired* or weariness* or exhaust* or malaise* or pain* or headache* or breathless* or breathing* or myalgia* or delirious* or delirium* or appetite* or muscle* or muscular* or fitness* or memory* or stress* or depress* or anxiety* or emotion* or cough* or fever* or temperatur* or pneumon* or conjunctivit* or throat* or pharyngit* or dyspnea* or dyspnoea* or sick* or nausea* or nauseous* or vomit* or diarrhoea* or diarrhea* or taste* or anosmia* or smell* or olfact* or sweat* or dehydrat* or pyrexi* or nasal* or nose* or mucus* or ear* or hearing* or deaf* or "brain fog*" or cardiac* or thoracic* or chest* or ischemic* or ischaemic* or heart* or liver* or hepatic* or immuno* or palpitation* or vertigo* or metabol* or vestibular* or endocrine* or encephalit* or physical* or cough* or fibrosis* or myocarditis* or Guillain* or barre* or neuralgi* or amyotroph* or thrombo* or clot* or rash* or hive* or urticari* or lymph* or stroke* or TIA or toe* or foot* or feet* or finger* or chilblain* or numb* or inflammat* or inflame* or arthralgi* or eye* or organ or organs or tingl* or sting* or burn* or bladder* or urogenit* or genitourin* or genital* or reproducti* or urinary* or joint* or tachycard* or atrial* or autoimmun* or dysautonomi* or polyneuro* or mast* or mobility* or walking* or ambulation* or energy*) NEAR/1 (sequela* or complication* or consequence* or complexit*) NEAR/10 (covid* or coronavirus* or “corona* virus*” or coronovirus* or “corono* virus*” or coronavirinae* or “corona* virinae*” or Cov or "2019-nCoV*" or 2019nCoV* or "19-nCoV*" or 19nCoV* or nCoV2019* or "nCoV-2019*" or nCoV19* or "nCoV-19*" or "HCoV-19*" or HCoV19* or "HCoV-2019*" or HCoV2019* or "2019 novel*" or Ncov* or "n-cov" or "SARS-CoV-2*" or "SARSCoV-2*" or "SARSCoV2*" or "SARS-CoV2*" or SARSCov19* or "SARS-Cov19*" or "SARSCov-19*" or "SARS-Cov-19*" or SARSCov2019* or "SARS-Cov2019*" or "SARSCov-2019*" or "SARS-Cov-2019*" or SARS2* or "SARS-2*" or SARScoronavirus2* or "SARS-coronavirus-2*" or "SARScoronavirus 2*" or "SARS coronavirus2*" or SARScoronovirus2* or "SARS-coronovirus-2*" or "SARScoronovirus 2*" or "SARS coronovirus2*" or "severe acute respiratory syndrome*")) |  | 3140 |
| #15 | TS=(((((multi or multiple* or overlap* or cluster* or numerous* or varied* or variety*) NEAR/1 (symptom* or system* or disease* or disorder* or illness* or condition* or syndrome*)) or (multisymptom* or multisystem* or multidisease* or multidisorder* or multiillness* or multicondition* or multisyndrom*)) NEAR/10 (covid* or coronavirus* or “corona* virus*” or coronovirus* or “corono* virus*” or coronavirinae* or “corona* virinae*” or Cov or "2019-nCoV*" or 2019nCoV* or "19-nCoV*" or 19nCoV* or nCoV2019* or "nCoV-2019*" or nCoV19* or "nCoV-19*" or "HCoV-19*" or HCoV19* or "HCoV-2019*" or HCoV2019* or "2019 novel*" or Ncov* or "n-cov" or "SARS-CoV-2*" or "SARSCoV-2*" or "SARSCoV2*" or "SARS-CoV2*" or SARSCov19* or "SARS-Cov19*" or "SARSCov-19*" or "SARS-Cov-19*" or SARSCov2019* or "SARS-Cov2019*" or "SARSCov-2019*" or "SARS-Cov-2019*" or SARS2* or "SARS-2*" or SARScoronavirus2* or "SARS-coronavirus-2*" or "SARScoronavirus 2*" or "SARS coronavirus2*" or SARScoronovirus2* or "SARS-coronovirus-2*" or "SARScoronovirus 2*" or "SARS coronovirus2*" or "severe acute respiratory syndrome*")) not ("Multisystem* inflammatory* syndrome*" or "inflammatory* multisystem* syndrome*")) |  | 1024 |
| #16 | #1 OR #2 OR #3 OR #4 OR #5 OR #6 OR #7 OR #8 OR #9 OR #10 OR #11 OR #12 OR #13 OR #14 OR #15 | Document types: Articles, Review Articles | 11877 |

## Cumulative Index to Nursing and Allied Health Literature (CINAHL)

| Platform | EBSCOhost |
| --- | --- |
| Search date | 09/05/2022 |
| No. of results | 3108 |

|  |  |  | No. of results |
| --- | --- | --- | --- |
| S1 | TI (longcovid* or long covid* or longcoronavirus* or longcorona* virus* or long coronavirus* or long corona* virus* or longcoronovirus* or longcorono* virus* or long coronovirus* or long corono* virus* or longcoronavirinae* or longcorona* virinae* or long coronavirinae* or long corona* virinae* or longCov or long Cov or longsars* or long sars* or "long severe acute respiratory syndrome*" or longncov* or long ncov* or longhcov* or long hcov*) | Expanders - Apply equivalent subjects Search modes - Boolean/Phrase | 688 |
| S2 | AB (longcovid* or long covid* or longcoronavirus* or longcorona* virus* or long coronavirus* or long corona* virus* or longcoronovirus* or longcorono* virus* or long coronovirus* or long corono* virus* or longcoronavirinae* or longcorona* virinae* or long coronavirinae* or long corona* virinae* or longCov or long Cov or longsars* or long sars* or "long severe acute respiratory syndrome*" or longncov* or long ncov* or longhcov* or long hcov*) | Expanders - Apply equivalent subjects Search modes - Boolean/Phrase | 919 |
| S3 | TI ((long* or endur* or legacy* or slow* or gradual* or protract* or lengthy* or chronic* or persist* or relaps* or remit* or remission* or residual* or delay* or prolong* or extend* or linger* or permanent* or fluctuat* or sequela* or multisystem* or "multi system*" or nonrecover* or "non recover*" or subacute* or "sub acute*" or lasting* or continuous* or continual* or continuing* or postacute* or "post acute*" or postdischarg* or "post discharg*" or postinfect* or "post infect*" or postviral* or "post viral*" or postvirus* or "post virus*") N1 (covid* or coronavirus* or corona* virus* or coronovirus* or corono* virus* or coronavirinae* or corona* virinae* or Cov or "2019-nCoV*" or 2019nCoV* or "19-nCoV*" or 19nCoV* or nCoV2019* or "nCoV-2019*" or nCoV19* or "nCoV-19*" or "HCoV-19*" or HCoV19* or "HCoV-2019*" or HCoV2019* or "2019 novel*" or Ncov* or "n-cov" or "SARS-CoV-2*" or "SARSCoV-2*" or "SARSCoV2*" or "SARS-CoV2*" or SARSCov19* or "SARS-Cov19*" or "SARSCov-19*" or "SARS-Cov-19*" or SARSCov2019* or "SARS-Cov2019*" or "SARSCov-2019*" or "SARS-Cov-2019*" or SARS2* or "SARS-2*" or SARScoronavirus2* or "SARS-coronavirus-2*" or "SARScoronavirus 2*" or "SARS coronavirus2*" or SARScoronovirus2* or "SARS-coronovirus-2*" or "SARScoronovirus 2*" or "SARS coronovirus2*" or "severe acute respiratory syndrome*")) | Expanders - Apply equivalent subjects Search modes - Boolean/Phrase | 817 |
| S4 | AB ((long* or endur* or legacy* or slow* or gradual* or protract* or lengthy* or chronic* or persist* or relaps* or remit* or remission* or residual* or delay* or prolong* or extend* or linger* or permanent* or fluctuat* or sequela* or multisystem* or "multi system*" or nonrecover* or "non recover*" or subacute* or "sub acute*" or lasting* or continuous* or continual* or continuing* or postacute* or "post acute*" or postdischarg* or "post discharg*" or  postinfect* or "post infect*" or postviral* or "post viral*" or postvirus* or "post virus*") N1 (covid* or coronavirus* or corona* virus* or coronovirus* or corono* virus* or coronavirinae* or corona* virinae* or Cov or "2019-nCoV*" or 2019nCoV* or "19-nCoV*" or 19nCoV* or nCoV2019* or "nCoV-2019*" or nCoV19* or "nCoV-19*" or "HCoV-19*" or HCoV19* or "HCoV-2019*" or HCoV2019* or "2019 novel*" or Ncov* or "n-cov" or "SARS-CoV-2*" or "SARSCoV-2*" or "SARSCoV2*" or "SARS-CoV2*" or SARSCov19* or "SARS-Cov19*" or "SARSCov-19*" or "SARS-Cov-19*" or SARSCov2019* or "SARS-Cov2019*" or "SARSCov-2019*" or "SARS-Cov-2019*" or SARS2* or "SARS-2*" or SARScoronavirus2* or "SARS-coronavirus-2*" or "SARScoronavirus 2*" or "SARS coronavirus2*" or SARScoronovirus2* or "SARS-coronovirus-2*" or "SARScoronovirus 2*" or "SARS coronovirus2*" or "severe acute respiratory syndrome*")) | Expanders - Apply equivalent subjects Search modes - Boolean/Phrase | 933 |
| S5 | TI (("long* term*" or longterm* or "long* haul*" or longhaul* or "long* tail*" or longtail* or longduration* or "long duration*" or longlast* or "long last*" or longstanding* or "long standing*" or "medium* term*" or mediumterm*) N3 (covid* or coronavirus* or corona* virus* or coronovirus* or corono* virus* or coronavirinae* or corona* virinae* or Cov or "2019-nCoV*" or 2019nCoV* or "19-nCoV*" or 19nCoV* or nCoV2019* or "nCoV-2019*" or nCoV19* or "nCoV-19*" or "HCoV-19*" or HCoV19* or "HCoV-2019*" or HCoV2019* or "2019 novel*" or Ncov* or "n-cov" or "SARS-CoV-2*" or "SARSCoV-2*" or "SARSCoV2*" or "SARS-CoV2*" or SARSCov19* or "SARS-Cov19*" or "SARSCov-19*" or "SARS-Cov-19*" or SARSCov2019* or "SARS-Cov2019*" or "SARSCov-2019*" or "SARS-Cov-2019*" or SARS2* or "SARS-2*" or SARScoronavirus2* or "SARS-coronavirus-2*" or "SARScoronavirus 2*" or "SARS coronavirus2*" or SARScoronovirus2* or "SARS-coronovirus-2*" or "SARScoronovirus 2*" or "SARS coronovirus2*" or "severe acute respiratory syndrome*")) | Expanders - Apply equivalent subjects Search modes - Boolean/Phrase | 300 |
| S6 | AB (("long* term*" or longterm* or "long* haul*" or longhaul* or "long* tail*" or longtail* or longduration* or "long duration*" or longlast* or "long last*" or longstanding* or "long standing*" or "medium* term*" or mediumterm*) N3 (covid* or coronavirus* or corona* virus* or coronovirus* or corono* virus* or coronavirinae* or corona* virinae* or Cov or "2019-nCoV*" or 2019nCoV* or "19-nCoV*" or 19nCoV* or nCoV2019* or "nCoV-2019*" or nCoV19* or "nCoV-19*" or "HCoV-19*" or HCoV19* or "HCoV-2019*" or HCoV2019* or "2019 novel*" or Ncov* or "n-cov" or "SARS-CoV-2*" or "SARSCoV-2*" or "SARSCoV2*" or "SARS-CoV2*" or SARSCov19* or "SARS-Cov19*" or "SARSCov-19*" or "SARS-Cov-19*" or SARSCov2019* or "SARS-Cov2019*" or "SARSCov-2019*" or "SARS-Cov-2019*" or SARS2* or "SARS-2*" or SARScoronavirus2* or "SARS-coronavirus-2*" or "SARScoronavirus 2*" or "SARS coronavirus2*" or SARScoronovirus2* or "SARS-coronovirus-2*" or "SARScoronovirus 2*" or "SARS coronovirus2*" or "severe acute respiratory syndrome*")) | Expanders - Apply equivalent subjects Search modes - Boolean/Phrase | 488 |
| S7 | TI ((postcovid* or post covid* or postcoronavirus* or postcorona* virus* or post coronavirus* or post corona* virus* or postcoronovirus* or postcorono* virus* or post coronovirus* or post corono* virus* or postcoronavirinae* or postcorona* virinae* or post coronavirinae* or post corona* virinae* or postCov or post Cov or postsars* or post sars* or "post severe acute respiratory syndrome*" or postncov* or post ncov* or posthcov* or post hcov*) N3 (syndrome* or disorder* or illness* or sickness* or disease* or condition* or symptom* or sign* or prognos* or followup* or "follow up*" or feature* or comorbid* or "co morbid*" or multimorbid* or "multi morbid*" or survivor* or survival* or risk* or care* or convalescen* or recuperat* or aftercare* or ambulatory* or outpatient* or "out patient*")) | Expanders - Apply equivalent subjects Search modes - Boolean/Phrase | 180 |
| S8 | AB ((postcovid* or post covid* or postcoronavirus* or postcorona* virus* or post coronavirus* or post corona* virus* or postcoronovirus* or postcorono* virus* or post coronovirus* or post corono* virus* or postcoronavirinae* or postcorona* virinae* or post coronavirinae* or post corona* virinae* or postCov or post Cov or postsars* or post sars* or "post severe acute respiratory syndrome*" or postncov* or post ncov* or posthcov* or post hcov*) N3 (syndrome* or disorder* or illness* or sickness* or disease* or condition* or symptom* or sign* or prognos* or followup* or "follow up*" or feature* or comorbid* or "co morbid*" or multimorbid* or "multi morbid*" or survivor* or survival* or risk* or care* or convalescen* or recuperat* or aftercare* or ambulatory* or outpatient* or "out patient*")) | Expanders - Apply equivalent subjects Search modes - Boolean/Phrase | 242 |
| S9 | TI ((ongoing* or long* or endur* or legacy* or slow* or gradual* or protract* or lengthy* or chronic* or persist* or relaps* or remit* or remission* or residual* or delay* or prolong* or extend* or linger* or permanent* or fluctuat* or multisystem* or "multi system*" or nonrecover* or "non recover*" or subacute* or "sub acute*" or lasting* or continuous* or continual* or continuing* or postacute* or "post acute*" or postdischarg* or "post discharg*" or postinfect* or "post infect*" or postviral* or "post viral*" or postvirus* or "post virus*" or "medium* term*" or mediumterm*) N4 (sequela* or illness* or symptom* or sign* or prognos* or rehab* or convalescen* or recuperat* or followup* or "follow up*" or feature*) N10 (covid* or coronavirus* or corona* virus* or coronovirus* or corono* virus* or coronavirinae* or corona* virinae* or Cov or "2019-nCoV*" or 2019nCoV* or "19-nCoV*" or 19nCoV* or nCoV2019* or "nCoV-2019*" or nCoV19* or "nCoV-19*" or "HCoV-19*" or HCoV19* or "HCoV-2019*" or HCoV2019* or "2019 novel*" or Ncov* or "n-cov" or "SARS-CoV-2*" or "SARSCoV-2*" or "SARSCoV2*" or "SARS-CoV2*" or SARSCov19* or "SARS-Cov19*" or "SARSCov-19*" or "SARS-Cov-19*" or SARSCov2019* or "SARS-Cov2019*" or "SARSCov-2019*" or "SARS-Cov-2019*" or SARS2* or "SARS-2*" or SARScoronavirus2* or "SARS-coronavirus-2*" or "SARScoronavirus 2*" or "SARS coronavirus2*" or SARScoronovirus2* or "SARS-coronovirus-2*" or "SARScoronovirus 2*" or "SARS coronovirus2*" or "severe acute respiratory syndrome*")) | Expanders - Apply equivalent subjects Search modes - Boolean/Phrase | 258 |
| S10 | AB ((ongoing* or long* or endur* or legacy* or slow* or  gradual* or protract* or lengthy* or chronic* or persist* or relaps* or remit* or remission* or residual* or delay* or prolong* or extend* or linger* or permanent* or fluctuat* or multisystem* or "multi system*" or nonrecover* or "non recover*" or subacute* or "sub acute*" or lasting* or continuous* or continual* or continuing* or postacute* or "post acute*" or postdischarg* or "post discharg*" or postinfect* or "post infect*" or postviral* or "post viral*" or postvirus* or "post virus*" or "medium* term*" or mediumterm*) N4 (sequela* or illness* or symptom* or sign* or prognos* or rehab* or convalescen* or recuperat* or followup* or "follow up*" or feature*) N10 (covid* or coronavirus* or corona* virus* or coronovirus* or corono* virus* or coronavirinae* or corona* virinae* or Cov or "2019-nCoV*" or 2019nCoV* or "19-nCoV*" or 19nCoV* or nCoV2019* or "nCoV-2019*" or nCoV19* or "nCoV-19*" or "HCoV-19*" or HCoV19* or "HCoV-2019*" or HCoV2019* or "2019 novel*" or Ncov* or "n-cov" or "SARS-CoV-2*" or "SARSCoV-2*" or "SARSCoV2*" or "SARS-CoV2*" or SARSCov19* or "SARS-Cov19*" or "SARSCov-19*" or "SARS-Cov-19*" or SARSCov2019* or "SARS-Cov2019*" or "SARSCov-2019*" or "SARS-Cov-2019*" or SARS2* or "SARS-2*" or SARScoronavirus2* or "SARS-coronavirus-2*" or "SARScoronavirus 2*" or "SARS coronavirus2*" or SARScoronovirus2* or "SARS-coronovirus-2*" or "SARScoronovirus 2*" or "SARS coronovirus2*" or "severe acute respiratory syndrome*")) | Expanders -  Apply equivalent subjects Search modes - Boolean/Phrase | 766 |
| S11 | TI ((ongoing* or long* or endur* or legacy* or slow* or gradual* or protract* or lengthy* or chronic* or persist* or relaps* or remit* or remission* or residual* or delay* or prolong* or extend* or linger* or permanent* or fluctuat* or multisystem* or "multi system*" or subacute* or "sub acute*" or lasting* or continuous* or continual* or continuing* or post* or after* or follow* or "medium* term*" or mediumterm*) N1 recover* N10 (covid* or coronavirus* or corona* virus* or coronovirus* or corono* virus* or coronavirinae* or corona* virinae* or Cov or "2019-nCoV*" or 2019nCoV* or "19-nCoV*" or 19nCoV* or nCoV2019* or "nCoV-2019*" or nCoV19* or "nCoV-19*" or "HCoV-19*" or HCoV19* or "HCoV-2019*" or HCoV2019* or "2019 novel*" or Ncov* or "n-cov" or "SARS-CoV-2*" or "SARSCoV-2*" or "SARSCoV2*" or "SARS-CoV2*" or SARSCov19* or "SARS-Cov19*" or "SARSCov-19*" or "SARS-Cov-19*" or SARSCov2019* or "SARS-Cov2019*" or "SARSCov-2019*" or "SARS-Cov-2019*" or SARS2* or "SARS-2*" or SARScoronavirus2* or "SARS-coronavirus-2*" or "SARScoronavirus 2*" or "SARS coronavirus2*" or SARScoronovirus2* or "SARS-coronovirus-2*" or "SARScoronovirus 2*" or "SARS coronovirus2*" or "severe acute respiratory syndrome*")) | Expanders - Apply equivalent subjects Search modes - Boolean/Phrase | 96 |
| S12 | AB ((ongoing* or long* or endur* or legacy* or slow* or gradual* or protract* or lengthy* or chronic* or persist* or relaps* or remit* or remission* or residual* or delay* or prolong* or extend* or linger* or permanent* or fluctuat* or multisystem* or "multi system*" or subacute* or "sub acute*" or lasting* or continuous* or continual* or continuing* or post* or after* or follow* or "medium* term*" or mediumterm*) N1 recover* N10 (covid* or coronavirus* or corona* virus* or coronovirus* or corono* virus* or coronavirinae* or corona* virinae* or Cov or "2019-nCoV*" or 2019nCoV* or "19-nCoV*" or 19nCoV* or nCoV2019* or "nCoV-2019*" or nCoV19* or "nCoV-19*" or "HCoV-19*" or HCoV19* or "HCoV-2019*" or HCoV2019* or "2019 novel*" or Ncov* or "n-cov" or "SARS-CoV-2*" or "SARSCoV-2*" or "SARSCoV2*" or "SARS-CoV2*" or SARSCov19* or "SARS-Cov19*" or "SARSCov-19*" or "SARS-Cov-19*" or SARSCov2019* or "SARS-Cov2019*" or "SARSCov-2019*" or "SARS-Cov-2019*" or SARS2* or "SARS-2*" or SARScoronavirus2* or "SARS-coronavirus-2*" or "SARScoronavirus 2*" or "SARS coronavirus2*" or SARScoronovirus2* or "SARS-coronovirus-2*" or "SARScoronovirus 2*" or "SARS coronovirus2*" or "severe acute respiratory syndrome*")) | Expanders - Apply equivalent subjects Search modes - Boolean/Phrase | 208 |
| S13 | S1 OR S2 OR S3 OR S4 OR S5 OR S6 OR S7 OR S8 OR S9 OR S10 OR S11 OR S12 | Expanders - Apply equivalent subjects Search modes - Boolean/Phrase | 3314 |
| S14 | (MH "Coronavirus+") | Expanders - Apply equivalent subjects Search modes - Boolean/Phrase | 2670 |
| S15 | (MH "Coronavirus Infections+") | Expanders - Apply equivalent subjects Search modes - Boolean/Phrase | 36612 |
| S16 | TI (covid* or coronavirus* or corona* virus* or coronovirus* or corono* virus* or coronavirinae* or corona* virinae* or Cov or "2019-nCoV*" or 2019nCoV* or "19-nCoV*" or 19nCoV* or nCoV2019* or "nCoV-2019*" or nCoV19* or "nCoV-19*" or "HCoV-19*" or HCoV19* or "HCoV-2019*" or HCoV2019* or "2019 novel*" or Ncov* or "n-cov" or "SARS-CoV-2*" or "SARSCoV-2*" or "SARSCoV2*" or "SARS-CoV2*" or SARSCov19* or "SARS-Cov19*" or "SARSCov-19*" or "SARS-Cov-19*" or SARSCov2019* or "SARS-Cov2019*" or "SARSCov-2019*" or "SARS-Cov-2019*" or SARS2* or "SARS-2*" or SARScoronavirus2* or "SARS-coronavirus-2*" or "SARScoronavirus 2*" or "SARS coronavirus2*" or SARScoronovirus2* or "SARS-coronovirus-2*" or "SARScoronovirus 2*" or "SARS coronovirus2*" or "severe acute respiratory syndrome*") | Expanders - Apply equivalent subjects Search modes - Boolean/Phrase | 74006 |
| S17 | S14 OR S15 OR S16 | Expanders - Apply equivalent subjects Search modes - Boolean/Phrase | 83220 |
| S18 | (MH "Functional Status") | Expanders - Apply equivalent subjects Search modes - Boolean/Phrase | 27079 |
| S19 | (MH "After Care") | Expanders - Apply equivalent subjects Search modes - Boolean/Phrase | 17862 |
| S20 | (MH "Rehabilitation") | Expanders - Apply equivalent subjects Search modes - Boolean/Phrase | 17317 |
| S21 | (MH "Activities of Daily Living") | Expanders - Apply equivalent subjects Search modes - Boolean/Phrase | 36009 |
| S22 | (MH "Recovery") | Expanders - Apply equivalent subjects Search modes - Boolean/Phrase | 36786 |
| S23 | (MH "Adverse Health Care Event") | Expanders - Apply equivalent subjects Search modes - Boolean/Phrase | 8949 |
| S24 | (MH "Treatment Complications, Delayed") | Expanders - Apply equivalent subjects Search modes - Boolean/Phrase | 2392 |
| S25 | (MH "Physical Performance") | Expanders - Apply equivalent subjects Search modes - Boolean/Phrase | 6089 |
| S26 | S18 OR S19 OR S20 OR S21 OR S22 OR S23 OR S24 OR S25 | Expanders - Apply equivalent subjects Search modes -  Boolean/Phrase | 141975 |
| S27 | S17 AND S26 | Expanders - Apply equivalent subjects Search modes - Boolean/Phrase | 1153 |
| S28 | S14 OR S15 | Expanders - Apply equivalent subjects Search modes - Boolean/Phrase | 37188 |
| S29 | TI ("long* haul*" or longhaul* or "long* tail*" or longtail* or longduration* or "long duration*" or longlast* or "long last*" or longstanding* or "long standing*") | Expanders - Apply equivalent subjects Search modes - Boolean/Phrase | 2303 |
| S30 | AB ("long* haul*" or longhaul* or "long* tail*" or longtail* or longduration* or "long duration*" or longlast* or "long last*" or longstanding* or "long standing*") | Expanders - Apply equivalent subjects Search modes - Boolean/Phrase | 15807 |
| S31 | S29 OR S30 | Expanders - Apply equivalent subjects Search modes - Boolean/Phrase | 17301 |
| S32 | S28 AND S31 | Expanders - Apply equivalent subjects Search modes - Boolean/Phrase | 174 |
| S33 | TI ((recover* or nonrecover*) N3 function* N10 (covid* or coronavirus* or corona* virus* or coronovirus* or corono* virus* or coronavirinae* or corona* virinae* or Cov or "2019-nCoV*" or 2019nCoV* or "19-nCoV*" or 19nCoV* or nCoV2019* or "nCoV-2019*" or nCoV19* or "nCoV-19*" or "HCoV-19*" or HCoV19* or "HCoV-2019*" or HCoV2019* or "2019 novel*" or Ncov* or "n-cov" or "SARS-CoV-2*" or "SARSCoV-2*" or "SARSCoV2*" or "SARS-CoV2*" or SARSCov19* or "SARS-Cov19*" or "SARSCov-19*" or "SARS-Cov-19*" or SARSCov2019* or "SARS-Cov2019*" or "SARSCov-2019*" or "SARS-Cov-2019*" or SARS2* or "SARS-2*" or SARScoronavirus2* or "SARS-coronavirus-2*" or "SARScoronavirus 2*" or "SARS coronavirus2*" or SARScoronovirus2* or "SARS-coronovirus-2*" or "SARScoronovirus 2*" or "SARS coronovirus2*" or "severe acute respiratory syndrome*")) | Expanders - Apply equivalent subjects Search modes - Boolean/Phrase | 10 |
| S34 | AB ((recover* or nonrecover*) N3 function* N10 (covid* or coronavirus* or corona* virus* or coronovirus* or corono* virus* or coronavirinae* or corona* virinae* or Cov or "2019-nCoV*" or 2019nCoV* or "19-nCoV*" or 19nCoV* or nCoV2019* or "nCoV-2019*" or nCoV19* or "nCoV-19*" or "HCoV-19*" or HCoV19* or "HCoV-2019*" or HCoV2019* or "2019 novel*" or Ncov* or "n-cov" or "SARS-CoV-2*" or "SARSCoV-2*" or "SARSCoV2*" or "SARS-CoV2*" or SARSCov19* or "SARS-Cov19*" or "SARSCov-19*" or "SARS-Cov-19*" or SARSCov2019* or "SARS-Cov2019*" or "SARSCov-2019*" or "SARS-Cov-2019*" or SARS2* or "SARS-2*" or SARScoronavirus2* or "SARS-coronavirus-2*" or "SARScoronavirus 2*" or "SARS coronavirus2*" or SARScoronovirus2* or "SARS-coronovirus-2*" or "SARScoronovirus 2*" or "SARS coronovirus2*" or "severe acute respiratory syndrome*")) | Expanders - Apply equivalent subjects Search modes - Boolean/Phrase | 19 |
| S35 | TI ((postacute* or "post acute*" or postdischarg* or "post discharg*" or postinfect* or "post infect*" or postviral* or "post viral*" or postvirus* or "post virus*" or subacute* or "sub acute*") N3 (care* or convalescen* or recuperat* or aftercare* or ambulatory* or outpatient* or "out patient*" or survivor* or survival*) N10 (covid* or coronavirus* or corona* virus* or coronovirus* or corono* virus* or coronavirinae* or corona* virinae* or Cov or "2019-nCoV*" or 2019nCoV* or "19-nCoV*" or 19nCoV* or nCoV2019* or "nCoV-2019*" or nCoV19* or "nCoV-19*" or "HCoV-19*" or HCoV19* or "HCoV-2019*" or HCoV2019* or "2019 novel*" or Ncov* or "n-cov" or "SARS-CoV-2*" or "SARSCoV-2*" or "SARSCoV2*" or "SARS-CoV2*" or SARSCov19* or "SARS-Cov19*" or "SARSCov-19*" or "SARS-Cov-19*" or SARSCov2019* or "SARS-Cov2019*" or "SARSCov-2019*" or "SARS-Cov-2019*" or SARS2* or "SARS-2*" or SARScoronavirus2* or "SARS-coronavirus-2*" or "SARScoronavirus 2*" or "SARS coronavirus2*" or SARScoronovirus2* or "SARS-coronovirus-2*" or "SARScoronovirus 2*" or "SARS coronovirus2*" or "severe acute respiratory syndrome*")) | Expanders - Apply equivalent subjects Search modes - Boolean/Phrase | 38 |
| S36 | AB ((postacute* or "post acute*" or postdischarg* or "post discharg*" or postinfect* or "post infect*" or postviral* or "post viral*" or postvirus* or "post virus*" or subacute* or "sub acute*") N3 (care* or convalescen* or recuperat* or aftercare* or ambulatory* or outpatient* or "out patient*" or survivor* or survival*) N10 (covid* or coronavirus* or corona* virus* or coronovirus* or corono* virus* or coronavirinae* or corona* virinae* or Cov or "2019-nCoV*" or 2019nCoV* or "19-nCoV*" or 19nCoV* or nCoV2019* or "nCoV-2019*" or nCoV19* or "nCoV-19*" or "HCoV-19*" or HCoV19* or "HCoV-2019*" or HCoV2019* or "2019 novel*" or Ncov* or "n-cov" or "SARS-CoV-2*" or "SARSCoV-2*" or "SARSCoV2*" or "SARS-CoV2*" or SARSCov19* or "SARS-Cov19*" or "SARSCov-19*" or "SARS-Cov-19*" or SARSCov2019* or "SARS-Cov2019*" or "SARSCov-2019*" or "SARS-Cov-2019*" or SARS2* or "SARS-2*" or SARScoronavirus2* or "SARS-coronavirus-2*" or "SARScoronavirus 2*" or "SARS coronavirus2*" or SARScoronovirus2* or "SARS-coronovirus-2*" or "SARScoronovirus 2*" or "SARS coronovirus2*" or "severe acute respiratory syndrome*")) | Expanders - Apply equivalent subjects Search modes - Boolean/Phrase | 34 |
| S37 | TI ((convalescen* or recuperat* or after* or followup* or "follow up*" or rehab*) N1 (therap* or care*) N10 (covid* or coronavirus* or corona* virus* or coronovirus* or corono* virus* or coronavirinae* or corona* virinae* or Cov or "2019-nCoV*" or 2019nCoV* or "19-nCoV*" or 19nCoV* or nCoV2019* or "nCoV-2019*" or nCoV19* or "nCoV-19*" or "HCoV-19*" or HCoV19* or "HCoV-2019*" or HCoV2019* or "2019 novel*" or Ncov* or "n-cov" or "SARS-CoV-2*" or "SARSCoV-2*" or "SARSCoV2*" or "SARS-CoV2*" or SARSCov19* or "SARS-Cov19*" or "SARSCov-19*" or "SARS-Cov-19*" or SARSCov2019* or "SARS-Cov2019*" or "SARSCov-2019*" or "SARS-Cov-2019*" or SARS2* or "SARS-2*" or SARScoronavirus2* or "SARS-coronavirus-2*" or "SARScoronavirus 2*" or "SARS coronavirus2*" or SARScoronovirus2* or "SARS-coronovirus-2*" or "SARScoronovirus 2*" or "SARS coronovirus2*" or "severe acute respiratory syndrome*")) | Expanders - Apply equivalent subjects Search modes - Boolean/Phrase | 105 |
| S38 | AB ((convalescen* or recuperat* or after* or followup* or "follow up*" or rehab*) N1 (therap* or care*) N10 (covid* or coronavirus* or corona* virus* or coronovirus* or corono* virus* or coronavirinae* or corona* virinae* or Cov or "2019-nCoV*" or 2019nCoV* or "19-nCoV*" or 19nCoV* or nCoV2019* or "nCoV-2019*" or nCoV19* or "nCoV-19*" or "HCoV-19*" or HCoV19* or "HCoV-2019*" or HCoV2019* or "2019 novel*" or Ncov* or "n-cov" or "SARS-CoV-2*" or "SARSCoV-2*" or "SARSCoV2*" or "SARS-CoV2*" or SARSCov19* or "SARS-Cov19*" or "SARSCov-19*" or "SARS-Cov-19*" or SARSCov2019* or "SARS-Cov2019*" or "SARSCov-2019*" or "SARS-Cov-2019*" or SARS2* or "SARS-2*" or SARScoronavirus2* or "SARS-coronavirus-2*" or "SARScoronavirus 2*" or "SARS coronavirus2*" or SARScoronovirus2* or "SARS-coronovirus-2*" or "SARScoronovirus 2*" or "SARS coronovirus2*" or "severe acute respiratory syndrome*")) | Expanders - Apply equivalent subjects Search modes - Boolean/Phrase | 125 |
| S39 | TI ((ongoing* or endur* or long* or legacy* or slow* or gradual* or protract* or lengthy* or chronic* or persist* or relaps* or remit* or remission* or residual* or delay* or prolong* or extend* or linger* or permanent* or fluctuat* or multisystem* or "multi system*" or nonrecover* or "non recover*" or subacute* or "sub acute*" or lasting* or continuous* or continual* or continuing* or postacute* or "post acute*" or postdischarg* or "post discharg*" or postinfect* or "post infect*" or postviral* or "post viral*" or postvirus* or "post virus*" or "medium* term*" or mediumterm* or adverse* or dangerous* or harmful* or indirect* or injurious* or secondary* or side effect* or undesirable* or sequela* or complication* or consequence* or effect* or event* or impact* or outcome* or reaction* or complexit* or aftercare* or impair* or problem* or issue* or rehab* or function* or perform*) N10 ((daily* or everyday* or day* or normal* or usual*) N1 (activit* or living* or life* or lives* or job* or work* or employ* or occupation* or hobby* or hobbies* or leisure*)) N10 (covid* or coronavirus* or corona* virus* or coronovirus* or corono* virus* or coronavirinae* or corona* virinae* or Cov or "2019-nCoV*" or 2019nCoV* or "19-nCoV*" or 19nCoV* or nCoV2019* or "nCoV-2019*" or nCoV19* or "nCoV-19*" or "HCoV-19*" or HCoV19* or "HCoV-2019*" or HCoV2019* or "2019 novel*" or Ncov* or "n-cov" or "SARS-CoV-2*" or "SARSCoV-2*" or "SARSCoV2*" or "SARS-CoV2*" or SARSCov19* or "SARS-Cov19*" or "SARSCov-19*" or "SARS-Cov-19*" or SARSCov2019* or "SARS-Cov2019*" or "SARSCov-2019*" or "SARS-Cov-2019*" or SARS2* or "SARS-2*" or SARScoronavirus2* or "SARS-coronavirus-2*" or "SARScoronavirus 2*" or "SARS coronavirus2*" or SARScoronovirus2* or "SARS-coronovirus-2*" or "SARScoronovirus 2*" or "SARS coronovirus2*" or "severe acute respiratory syndrome*")) | Expanders - Apply equivalent subjects Search modes - Boolean/Phrase | 17 |
| S40 | AB ((ongoing* or endur* or long* or legacy* or slow* or gradual* or protract* or lengthy* or chronic* or persist* or relaps* or remit* or remission* or residual* or delay* or prolong* or extend* or linger* or permanent* or fluctuat* or multisystem* or "multi system*" or nonrecover* or "non recover*" or subacute* or "sub acute*" or lasting* or continuous* or continual* or continuing* or postacute* or "post acute*" or postdischarg* or "post discharg*" or postinfect* or "post infect*" or postviral* or "post viral*" or postvirus* or "post virus*" or "medium* term*" or mediumterm* or adverse* or dangerous* or harmful* or indirect* or injurious* or secondary* or side effect* or undesirable* or sequela* or complication* or consequence* or effect* or event* or impact* or outcome* or reaction* or complexit* or aftercare* or impair* or problem* or issue* or rehab* or function* or perform*) N10 ((daily* or everyday* or day* or normal* or usual*) N1 (activit* or living* or life* or lives* or job* or work* or employ* or occupation* or hobby* or hobbies* or leisure*)) N10 (covid* or coronavirus* or corona* virus* or coronovirus* or corono* virus* or coronavirinae* or corona* virinae* or Cov or "2019-nCoV*" or 2019nCoV* or "19-nCoV*" or 19nCoV* or nCoV2019* or "nCoV-2019*" or nCoV19* or "nCoV-19*" or "HCoV-19*" or HCoV19* or "HCoV-2019*" or HCoV2019* or "2019 novel*" or Ncov* or "n-cov" or "SARS-CoV-2*" or "SARSCoV-2*" or "SARSCoV2*" or "SARS-CoV2*" or SARSCov19* or "SARS-Cov19*" or "SARSCov-19*" or "SARS-Cov-19*" or SARSCov2019* or "SARS-Cov2019*" or "SARSCov-2019*" or "SARS-Cov-2019*" or SARS2* or "SARS-2*" or SARScoronavirus2* or "SARS-coronavirus-2*" or "SARScoronavirus 2*" or "SARS coronavirus2*" or SARScoronovirus2* or "SARS-coronovirus-2*" or "SARScoronovirus 2*" or "SARS coronovirus2*" or "severe acute respiratory syndrome*")) | Expanders - Apply equivalent subjects Search modes - Boolean/Phrase | 159 |
| S41 | TI ((ongoing* or endur* or long* or legacy* or slow* or gradual* or protract* or lengthy* or chronic* or persist* or relaps* or remit* or remission* or residual* or delay* or prolong* or extend* or linger* or permanent* or fluctuat* or multisystem* or "multi system*" or nonrecover* or "non recover*" or subacute* or "sub acute*" or lasting* or continuous* or continual* or continuing* or postacute* or "post acute*" or postdischarg* or "post discharg*" or postinfect* or "post infect*" or postviral* or "post viral*" or postvirus* or "post virus*" or "medium* term*" or mediumterm*) N3 ((health* or adverse* or dangerous* or harmful* or indirect* or injurious* or secondary* or side* or undesirable* or negative* or damaging* or detriment* or abnormal*) N1 (sequela* or complication* or consequence* or effect* or event* or impact* or outcome* or reaction* or complexit* or aftercare* or impair* or problem* or issue* or symptom* or disorder*)) N10 (covid* or coronavirus* or corona* virus* or coronovirus* or corono* virus* or coronavirinae* or corona* virinae* or Cov or "2019-nCoV*" or 2019nCoV* or "19-nCoV*" or 19nCoV* or nCoV2019* or "nCoV-2019*" or nCoV19* or "nCoV-19*" or "HCoV-19*" or HCoV19* or "HCoV-2019*" or HCoV2019* or "2019 novel*" or Ncov* or "n-cov" or "SARS-CoV-2*" or "SARSCoV-2*" or "SARSCoV2*" or "SARS-CoV2*" or SARSCov19* or "SARS-Cov19*" or "SARSCov-19*" or "SARS-Cov-19*" or SARSCov2019* or "SARS-Cov2019*" or "SARSCov-2019*" or "SARS-Cov-2019*" or SARS2* or "SARS-2*" or SARScoronavirus2* or "SARS-coronavirus-2*" or "SARScoronavirus 2*" or "SARS coronavirus2*" or SARScoronovirus2* or "SARS-coronovirus-2*" or "SARScoronovirus 2*" or "SARS coronovirus2*" or "severe acute respiratory syndrome*")) | Expanders - Apply equivalent subjects Search modes - Boolean/Phrase | 21 |
| S42 | AB ((ongoing* or endur* or long* or legacy* or slow* or gradual* or protract* or lengthy* or chronic* or persist* or relaps* or remit* or remission* or residual* or delay* or prolong* or extend* or linger* or permanent* or fluctuat* or multisystem* or "multi system*" or nonrecover* or "non recover*" or subacute* or "sub acute*" or lasting* or continuous* or continual* or continuing* or postacute* or "post acute*" or postdischarg* or "post discharg*" or postinfect* or "post infect*" or postviral* or "post viral*" or postvirus* or "post virus*" or "medium* term*" or mediumterm* ) N3 ((health* or adverse* or dangerous* or harmful* or indirect* or injurious* or secondary* or side* or undesirable* or negative* or damaging* or detriment* or abnormal*) N1 (sequela* or complication* or consequence* or effect* or event* or impact* or outcome* or reaction* or complexit* or aftercare* or impair* or problem* or issue* or symptom* or disorder*)) N10 (covid* or coronavirus* or corona* virus* or coronovirus* or corono* virus* or coronavirinae* or corona* virinae* or Cov or "2019-nCoV*" or 2019nCoV* or "19-nCoV*" or 19nCoV* or nCoV2019* or "nCoV-2019*" or nCoV19* or "nCoV-19*" or "HCoV-19*" or HCoV19* or "HCoV-2019*" or HCoV2019* or "2019 novel*" or Ncov* or "n-cov" or "SARS-CoV-2*" or "SARSCoV-2*" or "SARSCoV2*" or "SARS-CoV2*" or SARSCov19* or "SARS-Cov19*" or "SARSCov-19*" or "SARS-Cov-19*" or SARSCov2019* or "SARS-Cov2019*" or "SARSCov-2019*" or "SARS-Cov-2019*" or SARS2* or "SARS-2*" or SARScoronavirus2* or "SARS-coronavirus-2*" or "SARScoronavirus 2*" or "SARS coronavirus2*" or SARScoronovirus2* or "SARS-coronovirus-2*" or "SARScoronovirus 2*" or "SARS coronovirus2*" or "severe acute respiratory syndrome*")) | Expanders - Apply equivalent subjects Search modes - Boolean/Phrase | 143 |
| S43 | TI ((ongoing* or endur* or long* or legacy* or slow* or gradual* or protract* or lengthy* or chronic* or persist* or relaps* or remit* or remission* or residual* or delay* or prolong* or extend* or linger* or permanent* or fluctuat* or multisystem* or "multi system*" or nonrecover* or "non recover*" or subacute* or "sub acute*" or lasting* or  continuous* or continual* or continuing* or postacute* or "post acute*" or postdischarg* or "post discharg*" or postinfect* or "post infect*" or postviral* or "post viral*" or postvirus* or "post virus*" or "medium* term*" or mediumterm*) N3 ((physiolog* or neuro* or cardio* or gastro* or musculo* or renal* or kidney* or cognitive* or cognition* or rheumato* or dermatol* or skin* or haematol* or blood* or autonomic* or nervous* or nervous system* or otolaryngol* or laryngol* or otolog* or cerebro* or brain* or vascular* or respirator* or lung* or pulmonary* or psycholog* or mental health* or mental* or psychiatr* or exertion* or debilit* or devitali* or enervat* or drain* or sleep* or weak* or tired* or frail* or sapp* or strength* or confusion* or letharg* or fatigue* or tired* or weariness* or exhaust* or malaise* or pain* or headache* or breathless* or breathing* or myalgia* or delirious* or delirium* or appetite* or muscle* or muscular* or fitness* or memory* or stress* or depress* or anxiety* or emotion* or cough* or fever* or temperatur* or pneumon* or conjunctivit* or throat* or pharyngit* or dyspnea* or dyspnoea* or sick* or nausea* or nauseous* or vomit* or diarrhoea* or diarrhea* or taste* or anosmia* or smell* or olfact* or sweat* or dehydrat* or pyrexi* or nasal* or nose* or mucus* or ear* or hearing* or deaf* or "brain fog*" or cardiac* or thoracic* or chest* or ischemic* or ischaemic* or heart* or liver* or hepatic* or immuno* or palpitation* or vertigo* or metabol* or vestibular* or endocrine* or encephalit* or physical* or cough* or fibrosis* or myocarditis* or Guillain* or barre* or neuralgi* or amyotroph* or thrombo* or clot* or rash* or hive* or urticari* or lymph* or stroke* or TIA or toe* or foot* or feet* or finger* or chilblain* or numb* or inflammat* or inflame* or arthralgi* or eye* or organ or organs or tingl* or sting* or burn* or bladder* or urogenit* or genitourin* or genital* or reproducti* or urinary* or joint* or tachycard* or atrial* or autoimmun* or dysautonomi* or polyneuro* or mast* or mobility* or walking* or ambulation* or energy*) N1 (sequela* or complication* or consequence* or effect* or event* or impact* or outcome* or reaction* or complexit* or aftercare* or impair* or problem* or issue* or symptom* or disorder* or abnormal*)) N10 (covid* or coronavirus* or corona* virus* or coronovirus* or corono* virus* or coronavirinae* or corona* virinae* or Cov or "2019-nCoV*" or 2019nCoV* or "19-nCoV*" or 19nCoV* or nCoV2019* or "nCoV-2019*" or nCoV19* or "nCoV-19*" or "HCoV-19*" or HCoV19* or "HCoV-2019*" or HCoV2019* or "2019 novel*" or Ncov* or "n-cov" or "SARS-CoV-2*" or "SARSCoV-2*" or "SARSCoV2*" or "SARS-CoV2*" or SARSCov19* or "SARS-Cov19*" or "SARSCov-19*" or "SARS-Cov-19*" or SARSCov2019* or "SARS-Cov2019*" or "SARSCov-2019*" or "SARS-Cov-2019*" or SARS2* or "SARS-2*" or SARScoronavirus2* or "SARS-coronavirus-2*" or "SARScoronavirus 2*" or "SARS coronavirus2*" or SARScoronovirus2* or "SARS-coronovirus-2*" or "SARScoronovirus 2*" or "SARS coronovirus2*" or "severe acute respiratory syndrome*")) | Expanders - Apply equivalent subjects Search modes - Boolean/Phrase | 86 |
| S44 | AB ((ongoing* or endur* or long* or legacy* or slow* or gradual* or protract* or lengthy* or chronic* or persist* or relaps* or remit* or remission* or residual* or delay* or prolong* or extend* or linger* or permanent* or fluctuat* or multisystem* or "multi system*" or nonrecover* or "non recover*" or subacute* or "sub acute*" or lasting* or continuous* or continual* or continuing* or postacute* or "post acute*" or postdischarg* or "post discharg*" or postinfect* or "post infect*" or postviral* or "post viral*" or postvirus* or "post virus*" or "medium* term*" or mediumterm*) N3 ((physiolog* or neuro* or cardio* or gastro* or musculo* or renal* or kidney* or cognitive* or cognition* or rheumato* or dermatol* or skin* or haematol* or blood* or autonomic* or nervous* or nervous or otolaryngol* or laryngol* or otolog* or cerebro* or brain* or vascular* or respirator* or lung* or pulmonary* or psycholog* or mental health* or mental* or psychiatr* or exertion* or debilit* or devitali* or enervat* or drain* or sleep* or weak* or tired* or frail* or sapp* or strength* or confusion* or letharg* or fatigue* or tired* or weariness* or exhaust* or malaise* or pain* or headache* or breathless* or breathing* or myalgia* or delirious* or delirium* or appetite* or muscle* or muscular* or fitness* or memory* or stress* or depress* or anxiety* or emotion* or cough* or fever* or temperatur* or pneumon* or conjunctivit* or throat* or pharyngit* or dyspnea* or dyspnoea* or sick* or nausea* or nauseous* or vomit* or diarrhoea* or diarrhea* or taste* or anosmia* or smell* or olfact* or sweat* or dehydrat* or pyrexi* or nasal* or nose* or mucus* or ear* or hearing* or deaf* or "brain fog*" or cardiac* or thoracic* or chest* or ischemic* or ischaemic* or heart* or liver* or hepatic* or immuno* or palpitation* or vertigo* or metabol* or vestibular* or endocrine* or encephalit* or physical* or cough* or fibrosis* or myocarditis* or Guillain* or barre* or neuralgi* or amyotroph* or thrombo* or clot* or rash* or hive* or urticari* or lymph* or stroke* or TIA or toe* or foot* or feet* or finger* or chilblain* or numb* or inflammat* or inflame* or arthralgi* or eye* or organ or organs or tingl* or sting* or burn* or bladder* or urogenit* or genitourin* or genital* or reproducti* or urinary* or joint* or tachycard* or atrial* or autoimmun* or dysautonomi* or polyneuro* or mast* or mobility* or walking* or ambulation* or energy*) N1 (sequela* or complication* or consequence* or effect* or event* or impact* or outcome* or reaction* or complexit* or aftercare* or impair* or problem* or issue* or symptom* or disorder* or abnormal*)) N10 (covid* or coronavirus* or corona* virus* or coronovirus* or corono* virus* or coronavirinae* or corona* virinae* or Cov or "2019-nCoV*" or 2019nCoV* or "19-nCoV*" or 19nCoV* or nCoV2019* or "nCoV-2019*" or nCoV19* or "nCoV-19*" or "HCoV-19*" or HCoV19* or "HCoV-2019*" or HCoV2019* or "2019 novel*" or Ncov* or "n-cov" or "SARS-CoV-2*" or "SARSCoV-2*" or "SARSCoV2*" or "SARS-CoV2*" or SARSCov19* or "SARS-Cov19*" or "SARSCov-19*" or "SARS-Cov-19*" or SARSCov2019* or "SARS-Cov2019*" or "SARSCov-2019*" or "SARS-Cov-2019*" or SARS2* or "SARS-2*" or SARScoronavirus2* or "SARS-coronavirus-2*" or "SARScoronavirus 2*" or "SARS coronavirus2*" or SARScoronovirus2* or "SARS-coronovirus-2*" or "SARScoronovirus 2*" or "SARS coronovirus2*" or "severe acute respiratory syndrome*")) | Expanders - Apply equivalent subjects Search modes - Boolean/Phrase | 231 |
| S45 | TI ((physiolog* or neuro* or cardio* or gastro* or musculo* or renal* or kidney* or cognitive* or cognition* or rheumato* or dermatol* or skin* or haematol* or blood* or autonomic* or nervous* or nervous system* or otolaryngol* or laryngol* or otolog* or cerebro* or brain* or vascular* or respirator* or lung* or pulmonary* or psycholog* or mental health* or mental* or psychiatr* or exertion* or debilit* or devitali* or enervat* or drain* or sleep* or weak* or tired* or frail* or sapp* or strength* or confusion* or letharg* or fatigue* or tired* or weariness* or exhaust* or malaise* or pain* or headache* or breathless* or breathing* or myalgia* or delirious* or delirium* or appetite* or muscle* or muscular* or fitness* or memory* or stress* or depress* or anxiety* or emotion* or cough* or fever* or temperatur* or pneumon* or conjunctivit* or throat* or pharyngit* or dyspnea* or dyspnoea* or sick* or nausea* or nauseous* or vomit* or diarrhoea* or diarrhea* or taste* or anosmia* or smell* or olfact* or sweat* or dehydrat* or pyrexi* or nasal* or nose* or mucus* or ear* or hearing* or deaf* or "brain fog*" or cardiac* or thoracic* or chest* or ischemic* or ischaemic* or heart* or liver* or hepatic* or immuno* or palpitation* or vertigo* or metabol* or vestibular* or endocrine* or encephalit* or physical* or cough* or fibrosis* or myocarditis* or Guillain* or barre* or neuralgi* or amyotroph* or thrombo* or clot* or rash* or hive* or urticari* or lymph* or stroke* or TIA or toe* or foot* or feet* or finger* or chilblain* or numb* or inflammat* or inflame* or arthralgi* or eye* or organ or organs or tingl* or sting* or burn* or bladder* or urogenit* or genitourin* or genital* or reproducti* or urinary* or joint* or tachycard* or atrial* or autoimmun* or dysautonomi* or polyneuro* or mast* or mobility* or walking* or ambulation* or energy*) N3 (postcovid* or post covid* or postcoronavirus* or postcorona* virus* or post coronavirus* or post corona* virus* or postcoronovirus* or postcorono* virus* or post coronovirus* or post corono* virus* or postcoronavirinae* or postcorona* virinae* or post coronavirinae* or post corona* virinae* or postCov or post Cov or postsars* or post sars* or "post severe acute respiratory syndrome*" or postncov* or post ncov* or posthcov* or post hcov*)) | Expanders - Apply equivalent subjects Search modes - Boolean/Phrase | 141 |
| S46 | AB ((physiolog* or neuro* or cardio* or gastro* or musculo* or renal* or kidney* or cognitive* or cognition* or rheumato* or dermatol* or skin* or haematol* or blood* or autonomic* or nervous* or nervous system* or otolaryngol* or laryngol* or otolog* or cerebro* or brain* or vascular* or respirator* or lung* or pulmonary* or psycholog* or mental health* or mental* or psychiatr* or exertion* or debilit* or devitali* or enervat* or drain* or sleep* or weak* or tired* or frail* or sapp* or strength* or confusion* or letharg* or fatigue* or tired* or weariness* or exhaust* or malaise* or pain* or headache* or breathless* or breathing* or myalgia* or delirious* or delirium* or appetite* or muscle* or muscular* or fitness* or memory* or stress* or depress* or anxiety* or emotion* or cough* or fever* or temperatur* or pneumon* or conjunctivit* or throat* or pharyngit* or dyspnea* or dyspnoea* or sick* or nausea* or nauseous* or vomit* or diarrhoea* or diarrhea* or taste* or anosmia* or smell* or olfact* or sweat* or dehydrat* or pyrexi* or nasal* or nose* or mucus* or ear* or hearing* or deaf* or "brain fog*" or cardiac* or thoracic* or chest* or ischemic* or ischaemic* or heart* or liver* or hepatic* or immuno* or palpitation* or vertigo* or metabol* or vestibular* or endocrine* or encephalit* or physical* or cough* or fibrosis* or myocarditis* or Guillain* or barre* or neuralgi* or amyotroph* or thrombo* or clot* or rash* or hive* or urticari* or lymph* or stroke* or TIA or toe* or foot* or feet* or finger* or chilblain* or numb* or inflammat* or inflame* or arthralgi* or eye* or organ or organs or tingl* or sting* or burn* or bladder* or urogenit* or genitourin* or genital* or reproducti* or urinary* or joint* or tachycard* or atrial* or autoimmun* or dysautonomi* or polyneuro* or mast* or mobility* or walking* or ambulation* or energy*) N3 (postcovid* or post covid* or postcoronavirus* or postcorona* virus* or post coronavirus* or post corona* virus* or postcoronovirus* or postcorono* virus* or post coronovirus* or post corono* virus* or postcoronavirinae* or postcorona* virinae* or post coronavirinae* or post corona* virinae* or postCov or post Cov or postsars* or post sars* or "post severe acute respiratory syndrome*" or postncov* or post ncov* or posthcov* or post hcov*)) | Expanders - Apply equivalent subjects Search modes - Boolean/Phrase | 144 |
| S47 | TI ((physiolog* or neuro* or cardio* or gastro* or musculo* or renal* or kidney* or cognitive* or cognition* or rheumato* or dermatol* or skin* or haematol* or blood* or autonomic* or nervous* or nervous system* or otolaryngol* or laryngol* or otolog* or cerebro* or brain* or vascular* or respirator* or lung* or pulmonary* or psycholog* or mental health* or mental* or psychiatr* or exertion* or debilit* or devitali* or enervat* or drain* or sleep* or weak* or tired* or frail* or sapp* or strength* or confusion* or letharg* or fatigue* or tired* or weariness* or exhaust* or malaise* or pain* or headache* or breathless* or breathing* or myalgia* or delirious* or delirium* or appetite* or muscle* or muscular* or fitness* or memory* or stress* or depress* or anxiety* or emotion* or cough* or fever* or temperatur* or pneumon* or conjunctivit* or throat* or pharyngit* or dyspnea* or dyspnoea* or sick* or nausea* or nauseous* or vomit* or diarrhoea* or diarrhea* or taste* or anosmia* or smell* or olfact* or sweat* or dehydrat* or pyrexi* or nasal* or nose* or mucus* or ear* or hearing* or deaf* or "brain fog*" or cardiac* or thoracic* or chest* or ischemic* or ischaemic* or heart* or liver* or hepatic* or immuno* or palpitation* or vertigo* or metabol* or vestibular* or endocrine* or encephalit* or physical* or cough* or fibrosis* or myocarditis* or Guillain* or barre* or neuralgi* or amyotroph* or thrombo* or clot* or rash* or hive* or urticari* or lymph* or stroke* or TIA or toe* or foot* or feet* or finger* or chilblain* or numb* or inflammat* or inflame* or arthralgi* or eye* or organ or organs or tingl* or sting* or burn* or bladder* or urogenit* or genitourin* or genital* or reproducti* or urinary* or joint* or tachycard* or atrial* or autoimmun* or dysautonomi* or polyneuro* or mast* or mobility* or walking* or ambulation* or energy*) N1 (sequela* or complication* or consequence* or complexit*) N10 (covid* or coronavirus* or corona* virus* or coronovirus* or corono* virus* or coronavirinae* or corona* virinae* or Cov or "2019-nCoV*" or 2019nCoV* or "19-nCoV*" or 19nCoV* or nCoV2019* or "nCoV-2019*" or nCoV19* or "nCoV-19*" or "HCoV-19*" or HCoV19* or "HCoV-2019*" or HCoV2019* or "2019 novel*" or Ncov* or "n-cov" or "SARS-CoV-2*" or "SARSCoV-2*" or "SARSCoV2*" or "SARS-CoV2*" or SARSCov19* or "SARS-Cov19*" or "SARSCov-19*" or "SARS-Cov-19*" or SARSCov2019* or "SARS-Cov2019*" or "SARSCov-2019*" or "SARS-Cov-2019*" or SARS2* or "SARS-2*" or SARScoronavirus2* or "SARS-coronavirus-2*" or "SARScoronavirus 2*" or "SARS coronavirus2*" or SARScoronovirus2* or "SARS-coronovirus-2*" or "SARScoronovirus 2*" or "SARS coronovirus2*" or "severe acute respiratory syndrome*")) | Expanders - Apply equivalent subjects Search modes - Boolean/Phrase | 237 |
| S48 | AB ((physiolog* or neuro* or cardio* or gastro* or musculo* or renal* or kidney* or cognitive* or cognition* or rheumato* or dermatol* or skin* or haematol* or blood* or autonomic* or nervous* or nervous system* or otolaryngol* or laryngol* or otolog* or cerebro* or brain* or vascular* or respirator* or lung* or pulmonary* or psycholog* or mental health* or mental* or psychiatr* or exertion* or debilit* or devitali* or enervat* or drain* or sleep* or weak* or tired* or frail* or sapp* or strength* or confusion* or letharg* or fatigue* or tired* or weariness* or exhaust* or malaise* or pain* or headache* or breathless* or breathing* or myalgia* or delirious* or delirium* or appetite* or muscle* or muscular* or fitness* or memory* or stress* or depress* or anxiety* or emotion* or cough* or fever* or temperatur* or pneumon* or conjunctivit* or throat* or pharyngit* or dyspnea* or dyspnoea* or sick* or nausea* or nauseous* or vomit* or diarrhoea* or diarrhea* or taste* or anosmia* or smell* or olfact* or sweat* or dehydrat* or pyrexi* or nasal* or nose* or mucus* or ear* or hearing* or deaf* or "brain fog*" or cardiac* or thoracic* or chest* or ischemic* or ischaemic* or heart* or liver* or hepatic* or immuno* or palpitation* or vertigo* or metabol* or vestibular* or endocrine* or encephalit* or physical* or cough* or fibrosis* or myocarditis* or Guillain* or barre* or neuralgi* or amyotroph* or thrombo* or clot* or rash* or hive* or urticari* or lymph* or stroke* or TIA or toe* or foot* or feet* or finger* or chilblain* or numb* or inflammat* or inflame* or arthralgi* or eye* or organ or organs or tingl* or sting* or burn* or bladder* or urogenit* or genitourin* or genital* or reproducti* or urinary* or joint* or tachycard* or atrial* or autoimmun* or dysautonomi* or polyneuro* or mast* or mobility* or walking* or ambulation* or energy*) N1 (sequela* or complication* or consequence* or complexit*) N10 (covid* or coronavirus* or corona* virus* or coronovirus* or corono* virus* or coronavirinae* or corona* virinae* or Cov or "2019-nCoV*" or 2019nCoV* or "19-nCoV*" or 19nCoV* or nCoV2019* or "nCoV-2019*" or nCoV19* or "nCoV-19*" or "HCoV-19*" or HCoV19* or "HCoV-2019*" or HCoV2019* or "2019 novel*" or Ncov* or "n-cov" or "SARS-CoV-2*" or "SARSCoV-2*" or "SARSCoV2*" or "SARS-CoV2*" or SARSCov19* or "SARS-Cov19*" or "SARSCov-19*" or "SARS-Cov-19*" or SARSCov2019* or "SARS-Cov2019*" or "SARSCov-2019*" or "SARS-Cov-2019*" or SARS2* or "SARS-2*" or SARScoronavirus2* or "SARS-coronavirus-2*" or "SARScoronavirus 2*" or "SARS coronavirus2*" or SARScoronovirus2* or "SARS-coronovirus-2*" or "SARScoronovirus 2*" or "SARS coronovirus2*" or  "severe acute respiratory syndrome*")) | Expanders - Apply equivalent subjects Search modes - Boolean/Phrase | 604 |
| S49 | TI (((((multi or multiple* or overlap* or cluster* or numerous* or varied* or variety*) N1 (symptom* or system* or disease* or disorder* or illness* or condition* or syndrome*)) or (multisymptom* or multisystem* or multidisease* or multidisorder* or multiillness* or multicondition* or multisyndrom*)) N10 (covid* or coronavirus* or corona* virus* or coronovirus* or corono* virus* or coronavirinae* or corona* virinae* or Cov or "2019-nCoV*" or 2019nCoV* or "19-nCoV*" or 19nCoV* or nCoV2019* or "nCoV-2019*" or nCoV19* or "nCoV-19*" or "HCoV-19*" or HCoV19* or "HCoV-2019*" or HCoV2019* or "2019 novel*" or Ncov* or "n-cov" or "SARS-CoV-2*" or "SARSCoV-2*" or "SARSCoV2*" or "SARS-CoV2*" or SARSCov19* or "SARS-Cov19*" or "SARSCov-19*" or "SARS-Cov-19*" or SARSCov2019* or "SARS-Cov2019*" or "SARSCov-2019*" or "SARS-Cov-2019*" or SARS2* or "SARS-2*" or SARScoronavirus2* or "SARS-coronavirus-2*" or "SARScoronavirus 2*" or "SARS coronavirus2*" or SARScoronovirus2* or "SARS-coronovirus-2*" or "SARScoronovirus 2*" or "SARS coronovirus2*" or "severe acute respiratory syndrome*")) not ("Multisystem* inflammatory* syndrome*" or "inflammatory* multisystem* syndrome*") | Expanders - Apply equivalent subjects Search modes - Boolean/Phrase | 52 |
| S50 | AB (((((multi or multiple* or overlap* or cluster* or numerous* or varied* or variety*) N1 (symptom* or system* or disease* or disorder* or illness* or condition* or syndrome*)) or (multisymptom* or multisystem* or multidisease* or multidisorder* or multiillness* or multicondition* or multisyndrom*)) N10 (covid* or coronavirus* or corona* virus* or coronovirus* or corono* virus* or coronavirinae* or corona* virinae* or Cov or "2019-nCoV*" or 2019nCoV* or "19-nCoV*" or 19nCoV* or nCoV2019* or "nCoV-2019*" or nCoV19* or "nCoV-19*" or "HCoV-19*" or HCoV19* or "HCoV-2019*" or HCoV2019* or "2019 novel*" or Ncov* or "n-cov" or "SARS-CoV-2*" or "SARSCoV-2*" or "SARSCoV2*" or "SARS-CoV2*" or SARSCov19* or "SARS-Cov19*" or "SARSCov-19*" or "SARS-Cov-19*" or SARSCov2019* or "SARS-Cov2019*" or "SARSCov-2019*" or "SARS-Cov-2019*" or SARS2* or "SARS-2*" or SARScoronavirus2* or "SARS-coronavirus-2*" or "SARScoronavirus 2*" or "SARS coronavirus2*" or SARScoronovirus2* or "SARS-coronovirus-2*" or "SARScoronovirus 2*" or "SARS coronovirus2*" or "severe acute respiratory syndrome*")) not ("Multisystem* inflammatory* syndrome*" or "inflammatory* multisystem* syndrome*") | Expanders - Apply equivalent subjects Search modes - Boolean/Phrase | 193 |
| S51 | S13 OR S27 OR S32 OR S33 OR S34 OR S35 OR S36 OR S37 OR S38 OR S39 OR S40 OR S41 OR S42 OR S43 OR S44 OR S45 OR S46 OR S47 OR S48 OR S49 OR S50 | Expanders - Apply equivalent subjects Search modes - Boolean/Phrase | 5649 |
| S52 | ((MH "animals+") OR (MH "animal studies") OR TI(animal model*)) | Expanders - Apply equivalent subjects Search modes - Boolean/Phrase | 237767 |
| S53 | (MH "human") | Expanders - Apply equivalent subjects Search modes - Boolean/Phrase | 2537337 |
| S54 | S52 not S53 | Expanders - Apply equivalent subjects Search modes - Boolean/Phrase | 204721 |
| S55 | S51 not S54 | Publication type – clinical trial, corrected article, journal article, meta analysis, practice guidelines, randomized controlled trial, research, review, systematic review  Expanders - Apply equivalent subjects Search modes - Boolean/Phrase | 3108 |

## PsycArticles

| Platform | ProQuest |
| --- | --- |
| Method | Only selected peer reviewed records. |
| Search date | 09/05/2022 |
| No. of results | 328 |

|  |  | No. of results |
| --- | --- | --- |
| S1 | TI,AB(longcovid* or long covid* or longcoronavirus* or longcorona* virus* or long coronavirus* or long corona* virus* or longcoronovirus* or longcorono* virus* or long coronovirus* or long corono* virus* or longcoronavirinae* or longcorona* virinae* or long coronavirinae* or long corona* virinae* or longCov or long Cov or longsars* or long sars* or "long severe acute respiratory syndrome*" or longncov* or long ncov* or longhcov* or long hcov* or postcovid* or post covid* or postcoronavirus* or postcorona* virus* or post coronavirus* or post corona* virus* or postcoronovirus* or postcorono* virus* or post coronovirus* or post corono* virus* or postcoronavirinae* or postcorona* virinae* or post coronavirinae* or post corona* virinae* or postCov or post Cov or postsars* or post sars* or "post severe acute respiratory syndrome*" or postncov* or post ncov* or posthcov* or post hcov*) | 117 |
| S2 | TI,AB(covid* or coronavirus* or coronavirus* or coronavirinae* or "SARS-CoV-2" or "SARSCoV-2" or "SARSCoV2" or SARScoronavirus2 or "SARS-coronavirus-2" or "SARScoronavirus 2" or "SARS coronavirus2" or SARScoronavirus2 or "SARS-coronavirus-2" or "SARScoronavirus 2" or "SARS coronavirus2" or "severe acute respiratory syndrome*") | 1051 |
| S3 | TI,AB(ongoing* or long* or endur* or legacy* or slow* or gradual* or protract* or lengthy* or chronic* or persist* or relaps* or remit* or remission* or residual* or delay* or prolong* or extend* or linger* or permanent* or fluctuat* or sequela* or multisystem* or "multi system*" or subacute* or "sub acute*" or lasting* or continuous* or continual* or continuing* or postacute* or "post acute*" or postdischarg* or "post discharg*" or postinfect* or "post infect*" or postviral* or "post viral*" or postvirus* or "post virus*" or "long* term*" or longterm* or "long* haul*" or longhaul* or "long* tail*" or longtail* or longduration* or "long duration*" or longlast* or "long last*" or longstanding* or "long standing*" or "medium* term*" or mediumterm* or rehab* or convalescen* or recuperat* or followup* or "follow up*" or recover* or nonrecover* or aftercare) | 48363 |
| S4 | S2 AND S3 | 303 |
| S5 | S1 OR S4 | 328 |

## PEDro

| Platform | pedro.org.au |
| --- | --- |
| Search date | 09/05/2022 |
| How the results were selected | Only downloaded the relevant references, based on their title. |
| No. of results | 81, downloaded 15.  26 reviews, downloaded 5.  55 clinical trials, downloaded 10. |

| Abstract & Title | COVID* |
| --- | --- |
| Method | Systematic Review  Clinical Trial  (Two separate searches) |

## EuropePMC

NOTE: This source provides open access to preprints of preliminary reports of work that have not been peer-reviewed. Preprints were not included in the final analysis, but it was checked whether the corresponding articles had already been published.

| URL | https://europepmc.org/ |
| --- | --- |
| Search date | 09/05/2022 |
| How the results were selected | Searched only for preprints. |
| Search strategies | (longcovid or “long covid” or longcoronavirus or “long coronavirus” or “long severe acute respiratory syndrome” or postcovid or “post covid” or postcoronavirus or “post coronavirus” or “post severe acute respiratory syndrome”) AND (remission or recovery or nonrecovery or rehabilitation or convalescence or recuperation or followup or "follow up" or aftercare) |
| No. of results | 1610 |

# Summary of Findings tables

## Physical training program

| **Summary of findings:** | | | |  |
| --- | --- | --- | --- | --- |
| **The effect of a physical training program in patients with persisting symptoms after COVID-19.** | | | |  |
| **Patient or population:** patients with persisting symptoms after COVID-19  **Setting:** ambulatory care  **Intervention:** a physical training program  **Comparison:** any or no | | | |  |
| Outcomes | Impact | № of participants (studies) | Certainty of the evidence (GRADE) |  |
|  |  |  |  |  |
| Dyspnoea assessed with: mMRC | One RCT showed decreased dyspnoea (mMRC) after training (RR for being dyspnoea-free at 6 weeks 1.46, 95%CI 1.17 to 1.82), but not at 28 weeks follow-up (RR 1.22, 95%CI 0.92 to 1.61). | 120 (1 RCT) | ⨁◯◯◯ Very low^a,b^ |  |
| Dyspnoea assessed with: Borg, mMRC, NPI, prevalence, VAS. | Four studies reported beneficial effects on dyspnoea (Borg, mMRC), while three studies reported no difference (prevalence, NPI, VAS). | 351 (7 observational studies) | ⨁◯◯◯ Very low^a,c,d,e^ |  |
| Physical capacity assessed with: 6MWT, squat time. | One RCT showed an increased 6-minute walking distance after training (difference between groups at 6 weeks: 65 meters, 95%CI 44 to 87), and also at 28 weeks follow-up (difference between groups: 69 meters, 95%CI 46 to 91).  Squat time also increased after training (difference between groups at 6 weeks: 20 seconds, 95%CI 12 to 28), and also at 28 weeks follow-up (difference between groups: 22 seconds, 95%CI 14 to 30). | 120 (1 RCT) | ⨁◯◯◯ Very low^a,b^ |  |
| Physical capacity assessed with: 6MWT, muscle strength, VO2 peak, W peak, STST, ISWT, ESWT, step test, handgrip strength. | Ten studies reported beneficial effects on physical capacity (6MWT, ESWT, handgrip strength, ISWT, muscle strength, step test, STST, VO2 peak, W peak), while one study reported mixed effects (significant improvement on 6MWT and STST, but no difference for handgrip strength). | 598 (11 observational studies) | ⨁◯◯◯ Very low^a,c,e,f^ |  |
| Pulmonary function assessed with: MVV | One RCT showed an increased MVV after training (difference between groups at 6 weeks: 10.57l/min, 95%CI 3.26 to 17.88), but not at 28 weeks follow-up (difference between groups: 5.2l/min, 95%CI -2.33 to 12.73). | 120 (1 RCT) | ⨁◯◯◯ Very low^a,b^ |  |
| Pulmonary function assessed with: FVC, VC, inspiratory breath hold test. | One study reported beneficial effects on pulmonary function (FVC), while one study reported mixed effects (significant improvement on the inspiratory breath hold test, but no difference for VC), and one study reported no difference (FVC). | 139 (3 observational studies) | ⨁◯◯◯ Very low^a,b,c,e^ |  |
| Quality of life assessed with: SF-12 | One RCT reported an improved physical quality of life (SF-12 PCS) after training (difference between groups at 6 weeks: 3.79, 95%CI 1.24 to 6.35) and at 28 weeks follow-up (difference between groups: 2.69, 95%CI 0.06 to 5.32). Mental quality of life (SF-12 MCS) did not improve after training (difference between groups at 6 weeks: 2.18, 95%CI -0.54 to 4.9), nor at 28 weeks follow-up (difference between groups: 1.99, 95%CI -0.81 to 4.79). | 120 (1 RCT) | ⨁◯◯◯ Very low^a,b^ |  |
| Quality of life assessed with: SF-12, SF-36, EQ-5D, EQ-5D-5L, VQ-11, SarQoL. | All eight studies reported beneficial effects on quality of life (EQ-5D, EQ-5D-5L, SarQoL, SF-12, SF-36, VQ-11). | 428 (8 observational studies) | ⨁◯◯◯ Very low^a,c,d,e,f^ |  |
| Fatigue assessed with: Prevalence, VAS, FACIT, MFI-20, ASS. | Four studies reported reduced fatigue levels after completing a physical training program (ASS, FACIT, MFI-20, prevalence, VAS), while one study reported no difference (prevalence). | 315 (5 observational studies) | ⨁◯◯◯ Very low^a,c,e^ |  |
| Muscle pain assessed with: Prevalence NRS. | Two studies reported no difference in muscle pain (NRS, prevalence) after completing a physical training program. | 66 (2 observational studies) | ⨁◯◯◯ Very low^a,b,d,e^ |  |
| Chest pain assessed with: Prevalence. | One study reported beneficial effects on the prevalence of chest pain, while one study reported no difference on its prevalence. | 113 (2 observational studies) | ⨁◯◯◯ Very low^a,b,c^ |  |
| Cognitive function assessed with: MoCA, prevalence. | One study reported beneficial effects on cognitive function (MoCA), while one study reported no difference (prevalence of memory impairment). | 104 (2 observational studies) | ⨁◯◯◯ Very low^a,b,c,e^ |  |
| Psychological well-being assessed with: HADS, PHQ-9, WAM. | One study reported beneficial effects on psychological well-being (WAM), while three studies reported no difference (HADS, PHQ-9). | 118 (4 observational studies) | ⨁◯◯◯ Very low^a,b,c,d,e^ |  |
| Return to daily life activities assessed with: N return to work. | One study reported no difference on return to work rates. Another study reported that 16 out of 22 participants resumed work immediately after the termination of the rehabilitation program. | 128 (2 observational studies) | ⨁◯◯◯ Very low^b,c^ |  |
| Functional capacity assessed with: CAT, FIQ. | Two studies reported beneficial effects on functional capacity (CAT, FIQ). | 57 (2 observational studies) | ⨁◯◯◯ Very low^a,b,d,e^ |  |
| **GRADE Working Group grades of evidence** **High certainty:** we are very confident that the true effect lies close to that of the estimate of the effect. **Moderate certainty:** we are moderately confident in the effect estimate: the true effect is likely to be close to the estimate of the effect, but there is a possibility that it is substantially different. **Low certainty:** our confidence in the effect estimate is limited: the true effect may be substantially different from the estimate of the effect. **Very low certainty:** we have very little confidence in the effect estimate: the true effect is likely to be substantially different from the estimate of effect. | | | |  |

Explanations

a. Indirectness because the intervention consists of a combination of a physical training program and other exercises.

b. Imprecision due to a low number of participants.

c. Study limitations (risk of bias) mainly due to an unacceptable cohort recruitment, failure to report or account for confounding factors, and inappropriate follow-up of subjects (CASP cohort study checklist).

d. High risk of bias in measurement of the outcome (Cochrane RoB 2.0).

e. Inconsistency because different scales are used to measure the outcome.

f. Some concerns (risk of bias) due to selection of the reported result (Cochrane RoB 2.0).

## Breathing exercises

| **Summary of findings:** | | | |  |
| --- | --- | --- | --- | --- |
| **The effect of breathing exercises in patients with persisting symptoms after COVID-19.** | | | |  |
| **Patient or population:** patients with persisting symptoms after COVID-19  **Setting:** ambulatory care  **Intervention:** breathing exercises  **Comparison:** any or no | | | |  |
| Outcomes | Impact | № of participants (studies) | Certainty of the evidence (GRADE) |  |
|  |  |  |  |  |
| Dyspnoea assessed with: mMRC, dyspnoea-12, VAS. | Two RCTs reported mixed effects on dyspnoea. One RCT showed decreased dyspnoea (mMRC) after training (RR for being dyspnoea-free at 6 weeks 1.46, 95%CI 1.17 to 1.82), but not at 28 weeks follow-up (RR 1.22, 95%CI 0.92 to 1.61). One RCT showed beneficial results for VAS breathlessness running (mean (SD) at follow-up: intervention group 71.47 (28.12), control group 81.48 (22.22); p=0.0026), but not for the dyspnoea-12 score, VAS breathlessness rest, VAS breathlessness walking, or the VAS breathlessness stairs. | 270 (2 RCTs) | ⨁◯◯◯ Very low^a,b,c,d^ |  |
| Dyspnoea assessed with: Borg, C19-YRS, mMRC, NPI, prevalence. | Four studies reported beneficial effects on dyspnoea (Borg, C19-YRS, mMRC), while two studies reported no difference (NPI, prevalence). | 324 (6 observational studies) | ⨁◯◯◯ Very low^b,c,e,f^ |  |
| Physical capacity assessed with: 6MWT, squat time. | Two RCTs found an improved 6-minute walking distance (RCT 1: difference between groups at 6 weeks 65.45 meters, 95%CI 43.8 to 87.2 and at 28 weeks 69 meters, 95%CI 46 to 91; RCT 2: after the intervention in the intervention group 212.3 meters (SD 82.5), control group 157.2 meters (SD 71.7), p<0.05). One RCT also reported an improved squat time: difference between the groups at 6 weeks was 20 seconds (95%CI 12 to 28) and at 22 weeks 22 seconds (95%CI 14 to 30). | 196 (2 RCTs) | ⨁◯◯◯ Very low^c,d,g^ |  |
| Physical capacity assessed with: 6MWT, STST, handgrip strength. | Three studies reported beneficial effects on physical capacity (6MWT, STST), while one study reported mixed effects (significant improvement on 6MWT and STST, but no difference for handgrip strength). | 270 (4 observational studies) | ⨁◯◯◯ Very low^b,c,d,e^ |  |
| Pulmonary function assessed with: MVV, FVC. | One RCT showed an increased MVV after training (difference 10.57 l/min, 95%CI 3.26 to 17.88), but not at 28 weeks follow-up (difference 5.2 l/min, 95%CI -2.33 to 12.73). One RCT showed an improvement in FVC for the intervention group (after the intervention 2.36 l, SD 0.49), compared to the control group (after the intervention 2.08 l, SD 0.37). Another RCT did not show differences in FVC between the intervention group (after the intervention 70.5 l, SD 5.5) and the control group (after the intervention 69.4 l, SD 5.9). | 244 (3 RCTs) | ⨁◯◯◯ Very low^b,c,d,h^ |  |
| Pulmonary function assessed with: VC, inspiratory breath hold test, FVC. | One study reported mixed effects on pulmonary function (significant improvement on the inspiratory breath hold test, but no difference for VC), while one study reported no difference (FVC). | 100 (2 observational studies) | ⨁◯◯◯ Very low^b,c,d,e^ |  |
| Psychological well-being assessed with: SAS, SDS, GAD-7. | One RCT found an improvement in anxiety, but not in depression: SAS anxiety intervention group 47.4 (SD 6.3), control group 54.9 (SD 7.3), p<0.05; SDS depression intervention group 54.5 (SD 5.9), control group 55.8 (SD 7.1), p>0.05. One RCT found no improvement in anxiety: mean (SD) at follow up intervention group 7.29 (5.81), control group 7.34 (5.37), p=0.085. | 226 (2 RCTs) | ⨁◯◯◯ Very low^b,d,h^ |  |
| Psychological well-being assessed with: C19-YRS, PHQ-9. | Two studies reported no difference for psychological well-being (C19-YRS, PHQ-9). | 54 (2 observational studies) | ⨁◯◯◯ Very low^b,c,d,f^ |  |
| Quality of life assessed with: SF-12, SF-36. | Three RCTs reported an improved quality of life. One RCT showed a significant improvement in all components of the SF-36: PCS mean (SD) intervention group 71.6 (7.6), control group 54.1 (7.5); MCS intervention group 73.7 (7.6), control group 62.1 (7.6). One RCT showed significant differences for SF-12 PCS only: PCS difference 3.79 (95%CI 1.24 to 6.35), at 28 weeks follow-up 2.69 (95% CI 0.06 to 5.32); MCS difference 2.18 (95% CI -0.54 to 4.9), at 28 weeks follow-up 1.99 (95% CI -0.81 to 4.79). One RCT showed significant differences for SF-36 MCS only: MCS intervention group 34.40 (11.97), control group 34.17 (10.37), p=0.047; PCS intervention group 34.02 (9.39), control group 33.30 (8.51), p=0.54. | 346 (3 RCTs) | ⨁◯◯◯ Very low^c,d,h^ |  |
| Quality of life assessed with: SF-12, SF-36, EQ-5D. | All four studies reported beneficial effects on quality of life (EQ-5D, SF-12, SF-36). | 271 (4 observational studies) | ⨁◯◯◯ Very low^b,c,d,e,f^ |  |
| Functional capacity assessed with: FIM, CAT. | Two RCTs found no improvement in functional capacity. One RCT found no difference in FIM after treatment in the intervention group 109.4 (SD 11.1) compared to the control group 108.9 (SD 10.1). One RCT found no improvement in CAT after treatment: intervention group 17.43 (SD 7.95), control group 17.76 (SD 7.15). | 226 (2 RCTs) | ⨁◯◯◯ Very low^b,d,h^ |  |
| Functional capacity assessed with: C19-YRS, FIQ. | One study reported beneficial effects on functional capacity (FIQ), while one study did not (C19-YRS). | 54 (2 observational studies) | ⨁◯◯◯ Very low^b,c,d,f^ |  |
| Fatigue assessed with: C19-YRS, VAS, prevalence. | All three studies reported beneficial effects on fatigue (C19-YRS, prevalence, VAS). | 251 (3 observational studies) | ⨁◯◯◯ Very low^b,c,d,e^ |  |
| Muscle pain assessed with: C19-YRS, NRS. | One study reported beneficial effects on muscle pain (C19-YRS), while one study did not (NRS). | 54 (2 observational studies) | ⨁◯◯◯ Very low^b,c,d,f^ |  |
| Chest pain assessed with: Prevalence. | One study reported beneficial effects on the prevalence of chest pain. | 74 (1 observational study) | ⨁◯◯◯ Very low^c,d,e^ |  |
| Cognitive function assessed with: C19-YRS, prevalence. | One study reported beneficial effects on cognitive function (C19-YRS), while one study reported no difference on the prevalence of memory impairment. | 101 (2 observational studies) | ⨁◯◯◯ Very low^b,c,d,e^ |  |
| Return to normal daily life activities assessed with: C19-YRS. | One study reported beneficial effects on return to normal daily life activities (C19-YRS). | 27 (1 observational study) | ⨁◯◯◯ Very low^d^ |  |
| **GRADE Working Group grades of evidence** **High certainty:** we are very confident that the true effect lies close to that of the estimate of the effect. **Moderate certainty:** we are moderately confident in the effect estimate: the true effect is likely to be close to the estimate of the effect, but there is a possibility that it is substantially different. **Low certainty:** our confidence in the effect estimate is limited: the true effect may be substantially different from the estimate of the effect. **Very low certainty:** we have very little confidence in the effect estimate: the true effect is likely to be substantially different from the estimate of effect. | | | |  |

Explanations

a. High risk of bias due to missing outcome data and in measurement of the outcome (Cochrane RoB 2.0).

b. Inconsistency because different scales are used to measure the outcome.

c. Indirectness because the intervention consists of a combination of breathing exercises and other exercises.

d. Imprecision due to a low number of participants.

e. Study limitations (risk of bias) mainly due to an unacceptable cohort recruitment, failure to report or account for confounding factors, and inappropriate follow-up of subjects (CASP cohort study checklist).

f. High risk of bias in measurement of the outcome (Cochrane RoB 2.0).

g. High risk of bias arising from the randomization process and in the measurement of the outcome (Cochrane RoB 2.0).

h. High risk of bias arising from the randomization process, in the measurement of the outcome, and due to missing outcome data (Cochrane RoB 2.0).

## Nutritional supplements

| **Summary of findings:** | | | |  |
| --- | --- | --- | --- | --- |
| **The effect of nutritional supplements in patients with persisting symptoms after COVID-19.** | | | |  |
| **Patient or population:** patients with persisting symptoms after COVID-19  **Setting:** ambulatory care  **Intervention:** nutritional supplements  **Comparison:** any or no | | | |  |
| **Palmitoylethanolamide and luteolin** | | | |  |
| Outcomes | Impact | № of participants (studies) | Certainty of the evidence (GRADE) |  |
|  |  |  |  |  |
| Sensory function assessed with: TDI score | One RCT showed an improved olfactory function after olfactory training combined with palmitoylethanolamide and luteolin (mean (SD) at 90 days follow-up: intervention group 29.8 (7.5), control group 19.5 (7.3); p<0.00001), while one RCT showed no difference (mean (SD) at 30 days follow-up: intervention group 25.2 (5.9), control group 31.1 (5.5); p=0.1). | 197 (2 RCTs) | ⨁◯◯◯ Very low^a,b,c^ |  |
| **Systemic enzymes (ImmunoSEB) and probiotics (ProbioSEB)** | | | |  |
| Outcomes | Impact | № of participants (studies) | Certainty of the evidence (GRADE) |  |
| Fatigue assessed with: CFQ-11 | One RCT showed reduced fatigue after taking systemic enzymes (ImmunoSEB) and probiotics (ProbioSEB) (mean at 14 days follow-up: intervention group 8.84, control group 19.91; p<0.001). | 200 (1 RCT) | ⨁◯◯◯ Very low^c,d^ |  |
| **Acetyl-carnitine** | | | |  |
| Outcomes | Impact | № of participants (studies) | Certainty of the evidence (GRADE) |  |
| Dyspnoea assessed with: NPI | One RCT showed no difference in dyspnoea after taking acetyl-carnitine in combination with a rehabilitation protocol (NPI mean (SD) one month after the end of the therapy intervention group 85.30 (6.02), control group 86.11 (4.96), p=0.79). | 60 (1 RCT) | ⨁◯◯◯ Very low^c,e,f^ |  |
| Muscle pain assessed with: NRS | One RCT showed reduced muscle pain after taking acetyl-carnitine in combination with a rehabilitation protocol (NRS mean (SD) one month after the end of the therapy intervention group 4.9 (0.75), control group 6.8 (0.92), p<0.05). | 60 (1 RCT) | ⨁◯◯◯ Very low^c,e,f^ |  |
| Psychological well-being assessed with: PHQ-9 | One RCT showed an improved psychological well-being after taking acetyl-carnitine in combination with a rehabilitation protocol (PHQ-9 mean (SD) one month after the end of the therapy intervention group 9.84 (3.02), control group 12.34 (2.89), p<0.05). | 60 (1 RCT) | ⨁◯◯◯ Very low^c,e,f^ |  |
| Quality of life assessed with: SF-12 | One study showed an improved quality of life after taking acetyl-carnitine in combination with a rehabilitation protocol (SF-12 mean (SD) one month after the end of the therapy intervention group 30.45 (2.49), control group 27.29 (3.16), p<0.05). | 60 (1 RCT) | ⨁◯◯◯ Very low^c,e,f^ |  |
| Functional capacity assessed with: FIQ | One RCT showed a significant improvement in functional capacity over time, but no significant difference between the groups (FIQ mean (SD) one month after the end of the therapy intervention group 41.96 (8.68), control group 43.03 (5.86), p=0.07). | 60 (1 RCT) | ⨁◯◯◯ Very low^c,e,f^ |  |
| **GRADE Working Group grades of evidence** **High certainty:** we are very confident that the true effect lies close to that of the estimate of the effect. **Moderate certainty:** we are moderately confident in the effect estimate: the true effect is likely to be close to the estimate of the effect, but there is a possibility that it is substantially different. **Low certainty:** our confidence in the effect estimate is limited: the true effect may be substantially different from the estimate of the effect. **Very low certainty:** we have very little confidence in the effect estimate: the true effect is likely to be substantially different from the estimate of effect. | | | |  |

Explanations

a. Both RCTs have a high risk of bias, due to the randomization process (D'Ascanio 2021), deviations from the intended interventions (D'Ascanio 2021), risk of bias in measurement of the outcome (Di Stadio 2022), and risk of bias in selection of the reported result (D'Ascanio 2021).

b. Indirectness because the intervention consists of a combination of nutritional supplements and olfactory training.

c. Imprecision due to a low number of participants.

d. Study limitations (some concerns) due to a possible conflict of interest.

e. High risk of bias in measurement of the outcome.

f. Indirectness because the intervention consists of a combination of nutritional supplements and a rehabilitation protocol.

## Olfactory training

| **Summary of findings:** | | | |  |
| --- | --- | --- | --- | --- |
| **The effect of olfactory training in patients with persisting symptoms after COVID-19.** | | | |  |
| **Patient or population:** patients with persisting symptoms after COVID-19  **Setting:** ambulatory care  **Intervention:** olfactory training  **Comparison:** any or no | | | |  |
| Outcomes | Impact | № of participants (studies) | Certainty of the evidence (GRADE) |  |
|  |  |  |  |  |
| Sensory function assessed with: VAS olfactory score, TDI score, discomfort experienced due to olfactory deficits, UPSIT, taste VAS. | Five studies reported beneficial effects on olfactory function (TDI, VAS olfactory score, VAS taste score, discomfort experienced due to olfactory deficits, UPSIT), while two studies reported no difference (TDI). | 799 (7 observational studies) | ⨁◯◯◯ Very low^a,b,c^ |  |
| Quality of life assessed with: Short-QOD-NS, SF-36. | One study reported beneficial effects on quality of life (SF-36, Short-QOD-NS). | 43 (1 observational study) | ⨁◯◯◯ Very low^d^ |  |
| **GRADE Working Group grades of evidence** **High certainty:** we are very confident that the true effect lies close to that of the estimate of the effect. **Moderate certainty:** we are moderately confident in the effect estimate: the true effect is likely to be close to the estimate of the effect, but there is a possibility that it is substantially different. **Low certainty:** our confidence in the effect estimate is limited: the true effect may be substantially different from the estimate of the effect. **Very low certainty:** we have very little confidence in the effect estimate: the true effect is likely to be substantially different from the estimate of effect. | | | |  |

Explanations

a. Study limitations (risk of bias) mainly due to failure to report confounding factors and unclear follow-up time (CASP cohort study checklist).

b. High risk of bias arising from the randomization process, due to deviations from the intended interventions, and in the measurement of the outcome (Cochrane RoB 2.0).

c. Inconsistency because different scales are used to measure the outcome.

d. Imprecision due to a low number of participants.

## Multidisciplinary treatment

| **Summary of findings:** | | | |  |
| --- | --- | --- | --- | --- |
| **The effect of multidisciplinairy treatment in patients with persisting symptoms after COVID-19.** | | | |  |
| **Patient or population:** patients with persisting symptoms after COVID-19  **Setting:** ambulatory care  **Intervention:** multidisciplinairy treatment  **Comparison:** any or no | | | |  |
| Outcomes | Impact | № of participants (studies) | Certainty of the evidence (GRADE) |  |
|  |  |  |  |  |
| Fatigue assessed with: MFIS, prevalence, BFI, FAS. | Three studies reported reduced fatigue levels (BFI, FAS, MFIS), while one study did not (prevalence). | 186 (4 observational studies) | ⨁◯◯◯ Very low^a,b^ |  |
| Dyspnoea assessed with: Borg, prevalence, VAS, mMRC. | Two studies reported reduced dyspnoea levels (Borg, mMRC, VAS), while one study reported mixed effects (improvement on mMRC, no improvement on Borg and prevalence). | 167 (3 observational studies) | ⨁◯◯◯ Very low^a,b^ |  |
| Physical capacity assessed with: Handgrip force, quadriceps force, SPPT, VO2, 6MWT, ESWT, STST, maximal workload. | All five studies reported beneficial effects on physical capacity (6MWT, ESWT, handgrip force, maximal workload, quadriceps force, SPPT, STST, VO2). | 232 (5 observational studies) | ⨁◯◯◯ Very low^a,b^ |  |
| Pulmonary function assessed with: MIP, FVC, VC. | All five studies reported beneficial effects on pulmonary function (FVC, MIP, VC). | 232 (5 observational studies) | ⨁◯◯◯ Very low^a,b^ |  |
| Cognitive function assessed with: Barcelona Test, Digit Span forward and backward, RAVLT, PMR task, MoCA, patient-reported cognitive deficit. | All three studies reported mixed effects on cognitive function. Significant improvements were observed for MoCA (by one study), verbal learning (RAVLT), long-term verbal memory (RAVLT), executive control (PMR task), and patient-reported cognitive deficit. No significant improvements were observed for MoCA (by one study), orientation in person, time, and space (Barcelona test), attention (Digit Span forward test), working memory (Digit Span backward test), and the prevalence of cognitive impairment. | 91 (3 observational studies) | ⨁◯◯◯ Very low^a,b^ |  |
| Psychological well-being assessed with: HADS, PHQ-9, GAD-7. | Two studies reported beneficial effects on psychological well-being (HADS, PHQ-9, GAD-7), while one study reported mixed effects (significant improvement in PHQ-9, but not in GAD-7) and one study reported no difference (HADS). | 144 (4 observational studies) | ⨁◯◯◯ Very low^a,b^ |  |
| Quality of life assessed with: WHOQOL-BREF, SF-36, EQ-5D, EQ-5D-5L | Two studies reported beneficial effects on quality of life (EQ-5D, EQ-5D-5L, WHOQOL-BREF), while two studies reported mixed effects (significant improvement on SF-36 mental component and EQ-5D VAS, but not on SF-36 physical component and EQ-5D index score). | 210 (4 observational studies) | ⨁◯◯◯ Very low^a,b^ |  |
| Return to normal daily life activities assessed with: N return to work. | One study reported beneficial effects on return to work. | 22 (1 observational study) | ⨁◯◯◯ Very low^b^ |  |
| Functional capacity assessed with: FIM, PCFS. | Two studies reported beneficial effects on functional capacity (FIM, PCFS). | 107 (2 observational studies) | ⨁◯◯◯ Very low^a,b^ |  |
| **GRADE Working Group grades of evidence** **High certainty:** we are very confident that the true effect lies close to that of the estimate of the effect. **Moderate certainty:** we are moderately confident in the effect estimate: the true effect is likely to be close to the estimate of the effect, but there is a possibility that it is substantially different. **Low certainty:** our confidence in the effect estimate is limited: the true effect may be substantially different from the estimate of the effect. **Very low certainty:** we have very little confidence in the effect estimate: the true effect is likely to be substantially different from the estimate of effect. | | | |  |

Explanations

a. Inconsistency because different scales are used to measure the outcome.

b. Imprecision due to a low number of participants.

## Other interventions

| **Summary of findings:** | | | |  |
| --- | --- | --- | --- | --- |
| **The effect of other interventions in patients with persisting symptoms after COVID-19.** | | | |  |
| **Patient or population:** patients with persisting symptoms after COVID-19  **Setting:** ambulatory care  **Intervention:** other interventions  **Comparison:** any or no | | | |  |
| **Narrative exposure therapy** | | | |  |
| Outcomes | Impact | № of participants (studies) | Certainty of the evidence (GRADE) |  |
|  |  |  |  |  |
| Psychological well-being assessed with: PCL-C, SDS, SAS | One RCT showed reduced PTSD at 6 month follow-up (PCL-C mean (SD) at 6 month follow-up intervention group 49.52 (7.32), control group 58.65 (7.48), p<0.001), but not for depression (SDS mean (SD) at 6 month follow-up intervention group 46.89 (8.95), control group 50.4 (8.98), p=0.127), nor for anxiety (SAS mean (SD) at 6 month follow-up intervention group 51.64 (9.5), control group 50.7 (10.23), p=0.146). PTSD, depression and anxiety symptoms significantly improved over time. | 111 (1 RCT) | ⨁◯◯◯ Very low^a,b^ |  |
| **Aromatherapy** | | | |  |
| Outcomes | Impact | № of participants (studies) | Certainty of the evidence (GRADE) |  |
| Fatigue assessed with: MFSI | One RCT showed reduced fatigue at 14 days follow-up (MFSI mean (SD) at day 14 intervention group 25.42 (3.10), control group 37.13 (3.10), p=0.020). | 44 (1 RCT) | ⨁⨁◯◯ Low^b^ |  |
| Psychological well-being assessed with: PHQ-9 | One RCT showed reduced depression symptoms at 14 days follow-up (PHQ-9 mean (SD) at day 14 intervention group 13.22 (0.70), control group 16.98 (0.70), p=0.002). | 44 (1 RCT) | ⨁⨁◯◯ Low^b^ |  |
| **Hydrogen inhalation** | | | |  |
| Outcomes | Impact | № of participants (studies) | Certainty of the evidence (GRADE) |  |
| Physical capacity assessed with: 6MWT | One RCT showed that, at day 10, the 6MWT was, on average, 30% higher in the intervention group than in the control group (p<0.0001). | 60 (1 RCT) | ⨁◯◯◯ Very low^b,c^ |  |
| **Massage techniques** | | | |  |
| Outcomes | Impact | № of participants (studies) | Certainty of the evidence (GRADE) |  |
| Fatigue assessed with: PFRS | One study reported reduced fatigue levels (PFRS). | 20 (1 observational study) | ⨁◯◯◯ Very low^b^ |  |
| Cognitive function assessed with: Stroop test, PFRS | Both studies showed an improved cognitive function (Stroop test, PFRS). | 80 (2 observational studies) | ⨁◯◯◯ Very low^b,d,e^ |  |
| Psychological well-being assessed with: Beck Depression Inventory, Spielberger-Khanin test, PFRS. | One study showed reduced depression (Beck Depression Inventory) and anxiety levels (Spielberger-Khanin test). The other study showed reduced emotional distress (PFRS). | 80 (2 observational studies) | ⨁◯◯◯ Very low^b,d,e^ |  |
| **Hyperbaric oxygen therapy** | | | |  |
| Outcomes | Impact | № of participants (studies) | Certainty of the evidence (GRADE) |  |
| Fatigue assessed with: Chalder fatigue scale | One study showed reduced fatigue levels (Chalder fatigue scale). | 10 (1 observational study) | ⨁◯◯◯ Very low^b,f^ |  |
| Cognitive function assessed with: NeuroTrax evaluation | One study showed a significant difference for global cognition, executive function, attention, information processing speed, and verbal function; but not for memory, visual-spatial and motor skills. | 10 (1 observational study) | ⨁◯◯◯ Very low^b,f^ |  |
| **Enhanced external counterpulsation** | | | |  |
| Outcomes | Impact | № of participants (studies) | Certainty of the evidence (GRADE) |  |
| Fatigue assessed with: PROMIS Fatigue Score | One study reported reduced fatigue levels (PROMIS Fatigue Score). | 16 (1 observational study) | ⨁◯◯◯ Very low^b,g^ |  |
| Dyspnoea assessed with: RDS | One study reported mixed effects on dyspnoea (RDS). | 16 (1 observational study) | ⨁◯◯◯ Very low^b,g^ |  |
| Chest pain assessed with: SAQ, CCS Angina Grade | One study reported mixed effects on chest pain (SAQ, CCS Angina Grade). | 16 (1 observational study) | ⨁◯◯◯ Very low^b,g^ |  |
| Physical capacity assessed with: 6MWT | One study reported beneficial effects on physical capacity (6MWT). | 16 (1 observational study) | ⨁◯◯◯ Very low^b,g^ |  |
| Cognitive function assessed with: prevalence of brain fog | One study reported reduced brain fog. | 16 (1 observational study) | ⨁◯◯◯ Very low^b,g^ |  |
| Psychological well-being assessed with: PHQ-2, PHQ-9 | One study reported reduced depression levels (PHQ-2, PHQ-9). | 16 (1 observational study) | ⨁◯◯◯ Very low^b,g^ |  |
| Return to normal daily life activities assessed with: return to work | One study reported that all six patients unable to work previously returned to work or school after the intervention. | 16 (1 observational study) | ⨁◯◯◯ Very low^b,g^ |  |
| Functional capacity assessed with: DASI | One study reported beneficial effects on functional capacity (DASI). | 16 (1 observational study) | ⨁◯◯◯ Very low^b,g^ |  |
| **GRADE Working Group grades of evidence** **High certainty:** we are very confident that the true effect lies close to that of the estimate of the effect. **Moderate certainty:** we are moderately confident in the effect estimate: the true effect is likely to be close to the estimate of the effect, but there is a possibility that it is substantially different. **Low certainty:** our confidence in the effect estimate is limited: the true effect may be substantially different from the estimate of the effect. **Very low certainty:** we have very little confidence in the effect estimate: the true effect is likely to be substantially different from the estimate of effect. | | | |  |

Explanations

a. Study limitations (some concerns) arising from the randomization process, in measurement of the outcome, and in selection of the reported result (Cochrane RoB 2.0).

b. Imprecision due to a low number of participants.

c. High risk of bias arising from the randomization process (Cochrane RoB 2.0).

d. Study limitations (risk of bias) because the follow-up of subjects was not long enough (CASP cohort study checklist).

e. Inconsistency because different scales are used to measure the outcome.

f. Study limitations (risk of bias) due to failure to account for confounding factors and because the follow-up of subjects was not long enough (CASP cohort study checklist).

g. Study limitations (risk of bias) in measurement of the exposure and because the follow-up of subjects was not long enough (CASP cohort study checklist).

## List of abbreviations

6MWT: 6-Minute Walking Test

ASS: Asthenic State Scale

BFI: Brief Fatigue Inventory

C19-YRS: COVID-19 Yorkshire Rehabilitation Scale

CI: confidence interval

CAT: COPD Assessment Test

CCS: Canadian Cardiovascular Society

DASI: Duke Activity Status Index

ESWT: Endurance Shuttle Walking Test

EQ-5D: EuroQol 5 dimensions

EQ-5D-5L: EuroQol 5 dimensions 5 levels

FACIT: Functional Assessment of Chronic Illness Therapy Fatigue Scale

FAS: Fatigue Assessment Scale

FIM: Functional Independence Measure

FIQ: Fibromyalgia Impact Questionnaire

FVC: forced vital capacity

GAD-7: General Anxiety Disorder 7 questionnaire

HADS: Hospital Anxiety and Depression Scale

ISWT: Incremental Shuttle Walking Test

l/min: liters per minute

MFI-20: Multidimensional Fatigue Inventory

MFIS: Modified Fatigue Impact Scale

MFSI: Multidimensional Fatigue Symptom Inventory

MIP: maximal inspiratory pressure

mMRC: (modified) Medical Research Council Dyspnoea

MoCA: Montreal Cognitive Assessment

MVV: maximum voluntary ventilation

N: number

NPI: Barthel Dyspnea Index

NRS: numeric rating scale

PCFS : Post-COVID-19 Functional Status

PCL-C: PTSD CheckList – Civilian Version

PFRS: profile of fatigue-related states

PHQ-2 / -9 : Patient Health Questionnaire 2 / 9

PMR task: a Spanish version of the FAS letter fluency task

PROMIS: Patient Reported Outcome Measurement Information System

PTSD: Post-traumatic stress disorder

RAVLT: Rey Auditory Verbal Learning Test

RCT: randomized controlled trial

RDS: Rose Dyspnea Scale

RR: risk ratio

SAQ: Seattle Angina Questionnaire

SarQoL: Sarcopenia and Quality of Life questionnaire

SAS: Self-rating Anxiety Scale

SD: standard deviation

SDS: Self-rating Depression Scale

SF-12/-36 (PCS) (MCS): 12-/36-Item Short Form Health Survey (Physical Component Score) (Mental Component Score)

Short-QOD-NS: short version of Questionnaire of Olfactory Disorders

SPPT: Short Physical Performance Test

STST: Sit-To-Stand Test

TDI: Threshold Discrimination Identification Sniffin’ Stick score

UPSIT: University of Pennsylvania Smell Identification Test

VAS: visual analogue scale

VC: vital capacity

VO2 (peak): (peak) aerobic exercise capacity

VQ-11: COPD-specific health-related quality of life

W: watt

WAM: self-assessment of Well-being, Activity and Mood

WHOQoL-BREF: World Health Organization Qualitiy of Life Questionnaire – BREF
